# Supplementary material for: Use of simulation training to teach the ABCDE primary assessment: an observational study in a Dutch University Hospital with a 3–4 months follow-up
Source: BMJ Open. 2020 Jul 6;10(7):e032023. doi: 10.1136/bmjopen-2019-032023 (PMC7342483; doi:10.1136/bmjopen-2019-032023)
Supplement: Author's manuscript [file bmjopen-2019-032023.draft_revisions.pdf]

BMJ Open is committed to open peer review. As part of this commitment we make the peer review history of every article we publish publicly available.

When an article is published we post the peer reviewers' comments and the authors' responses online. We also post the versions of the paper that were used during peer review. These are the versions that the peer review comments apply to.

The versions of the paper that follow are the versions that were submitted during the peer review process. They are not the versions of record or the final published versions. They should not be cited or distributed as the published version of this manuscript.

BMJ Open is an open access journal and the full, final, typeset and author-corrected version of record of the manuscript is available on our site with no access controls, subscription charges or pay-per-view fees (http://bmjopen.bmj.com).

If you have any questions on BMJ Open's open peer review process please email info.bmjopen@bmj.com

BMJ Open

# Simulation training is an effective educational tool to teach the ABCDE primary assessment: a blinded longitudinal intervention study

| | |
|---|---|
| Journal: | *BMJ Open* |
| Manuscript ID | bmjopen-2019-032023 |
| Article Type: | Research |
| Date Submitted by the Author: | 29-May-2019 |
| Complete List of Authors: | Drost- de Klerck, Amanda; Universitair Medisch Centrum Groningen, emergency department<br>Olgers, Tycho; University Medical Center Groningen, Internal medicine, Emergency dept.<br>van de Meeberg, Evelien; Universitair Medisch Centrum Groningen, emergency department<br>Schonrock-Adema, Johanna; Universitair Medisch Centrum Groningen, emergency department<br>ter Maaten, Jan; University Medical Center Groningen, Emergency Department, Department of internal medicine |
| Keywords: | ACCIDENT & EMERGENCY MEDICINE, EDUCATION & TRAINING (see Medical Education & Training), GENERAL MEDICINE (see Internal Medicine), MEDICAL EDUCATION & TRAINING |
| | |

SCHOLARONE™
Manuscripts

*I, the Submitting Author has the right to grant and does grant on behalf of all authors of the Work (as defined in the below author licence), an exclusive licence and/or a non-exclusive licence for contributions from authors who are: i) UK Crown employees; ii) where BMJ has agreed a CC-BY licence shall apply, and/or iii) in accordance with the terms applicable for US Federal Government officers or employees acting as part of their official duties; on a worldwide, perpetual, irrevocable, royalty-free basis to BMJ Publishing Group Ltd ("BMJ") its licensees and where the relevant Journal is co-owned by BMJ to the co-owners of the Journal, to publish the Work in this journal and any other BMJ products and to exploit all rights, as set out in our licence.*

*The Submitting Author accepts and understands that any supply made under these terms is made by BMJ to the Submitting Author unless you are acting as an employee on behalf of your employer or a postgraduate student of an affiliated institution which is paying any applicable article publishing charge ("APC") for Open Access articles. Where the Submitting Author wishes to make the Work available on an Open Access basis (and intends to pay the relevant APC), the terms of reuse of such Open Access shall be governed by a Creative Commons licence – details of these licences and which Creative Commons licence will apply to this Work are set out in our licence referred to above.*

*Other than as permitted in any relevant BMJ Author's Self Archiving Policies, I confirm this Work has not been accepted for publication elsewhere, is not being considered for publication elsewhere and does not duplicate material already published. I confirm all authors consent to publication of this Work and authorise the granting of this licence.*

For peer review only

# Simulation training is an effective educational tool to teach the ABCDE primary assessment: a blinded longitudinal intervention study.

*Amanda M Drost-de Klerck, Tycho J Olgers , Evelien K van de Meeberg, Johanna Schönrock-Adema , Jan C ter Maaten.*

*Emergency Department, University Medical Centre Groningen, Hanzeplein 1, 9713 GZ Groningen, The Netherlands, Amanda M Drost-de Klerck, Consultant Emergency Medicine. Emergency Department, University Medical Centre Groningen, Tycho J Olgers, Consultant Acute Internal Medicine. Emergency Department, University Medical Centre Groningen, Evelien K van de Meeberg, Consultant Emergency Medicine. University of Groningen, University Medical Centre Groningen, Institute for Medical Education, Johanna Schönrock-Adema, senior researcher. Emergency Department, University Medical Centre Groningen, Jan C ter Maaten, Full Professor Acute Internal Medicine.*

For peer review only

**Word count:** 2083

**Address for correspondence/request for reprints:**

A.M. Drost-de Klerck

Phone: +31503616161 / Fax: +31503611725

Email: a.drost@umcg.nl



For peer review only - http://bmjopen.bmj.com/site/about/guidelines.xhtml

**Contributors**

AMDK and JCM have conceived the study and created the assessment form. AMDK, TJO, EKM and JCM collected the data. AMDK is guarantor and did the literature search, managed the data and performed data analysis and drafted the manuscript. JSA advised regarding to the data analysis. All authors contributed to the final manuscript, revision of the manuscript and final approval of the manuscript. The corresponding author attests that all listed authors meet authorship criteria and that no others meeting the criteria have been omitted.

**Copyright/license for publication**

The corresponding author has the right to grant on behalf of all authors and does grant on behalf of all authors, a worldwide licence to the Publishers and its licensees in perpetuity, in all forms, formats and media (whether known now or created in the future), to i) publish, reproduce, distribute, display and store the Contribution, ii) translate the Contribution into other languages, create adaptations, reprints, include within collections and create summaries, extracts and/or, abstracts of the Contribution, iii) create any other derivative work(s) based on the Contribution, iv) to exploit all subsidiary rights in the Contribution, v) the inclusion of electronic links from the Contribution to third party material where-ever it may be located; and, vi) licence any third party to do any or all of the above."

**Competing interests**

All authors have completed the ICMJE uniform disclosure form at www.icmje.org/coi_disclosure.pdf and declare: no support from any organisation for the

For peer review only

submitted work; no financial relationships with any organisations that might have an interest in the submitted work in the previous three years; no other relationships or activities that could appear to have influenced the submitted work.

**Patient and public involvement**

Participants of this study were not involved in the development of the research question, design or outcome measures.  Some participants of the study encouraged others to participate, but they were all voluntarily included. The results of the study will be available for the participants on request.

**Ethics approval**

Ethics approval was not required because the study participants had signed up for a two day course,  the intervention,  before they were asked to participate in the study. So, the intervention was not on behalf of the study.

**Transparency**

We declare that the manuscript is honest, accurate and transparent. No important aspects of the study have been omitted and discrepancies from the study as originally planned have been explained.

**Funding**

The study was only funded by the Emergency Department of the University Medical Center Groningen.



For peer review only - http://bmjopen.bmj.com/site/about/guidelines.xhtml

## ABSTRACT

**Objectives** To analyse the short- and long-term effectiveness of simulation training to acquire a structured Airway Breathing Circulation Disability Exposure (ABCDE) approach for medical emergencies; and to analyse which skills were learned and maintained best.

**Design** a blinded longitudinal intervention study.

**Setting** The skills center of the University Medical Centre Groningen.

**Participants** Thirty voluntary participants of a simulation-based course were included in this study. Their mean age was 27 years. Twenty-one female, nine male.

**Intervention** The intervention consisted of a two day ABCDE-teaching course for residents and non-residents. The course encompasses 24 simulations with a patient simulator in which participants perform primary assessments of acute ill patients.

Video recordings were taken of each participant performing a primary assessment, before (T1), directly after (T2) and three months after the intervention (T3).

**Main outcome measures** The performance on the primary assessment was measured by the mean total rank score, based on video recordings that were taken before (T1), directly after (T2) and three months after the intervention (T3). Two observers scored the videos using an assessment form, in random order and blinded to the measurement moment.

**Results** The Wilcoxon signed rank test showed that the performance on the primary assessment was much better directly after the course (mean total rank score T2 vs T1 p 0.000) and this persisted till three months after the course for most skills. The skills that deteriorated 3 months after the course, remained significant better than before the course. Strikingly, most skills that decrease over time are skills related to communication and leadership.

**Conclusion**




For peer review only - http://bmjopen.bmj.com/site/about/guidelines.xhtml

Simulation training is an effective educational tool to teach doctors the ABCDE primary assessment. Communication skills decrease over time, so it could be useful to organize refresher courses, team training or another kind of training with a focus on communication and leadership.

**Strengths and limitations of this study**

**Strengths**

- This is a longitudinal intervention study using the same environment for intervention and study.
- The observers rated the videos in random order and were blinded to the measurement moment.

**Limitations**

- A few skills were often scored as "does not apply", which rendered the number of observations for those skills too few to analyse reliable differences. This was probably caused by the fact that scenarios contained a life threatening condition only in 3 of the 5 main items from the ABCDE. We think that this limitation wasn't of any influence on the results because when an item is scored often as "does not apply" it doesn't help to discriminate in quality of performance.
- The participants knew they were a study subject. We do not know if they have prepared for the study scenario. This might have influenced the study scores at T1 and T3.

For peer review only

| Key messages |
|---|
| **What is already known on this subject?**<br>➢ Using a structured “Airway-Breathing-Circulation-Disability-Exposure” (ABCDE) approach in the primary assessment for early recognition and immediate treatment of life threatening conditions has become the standard approach in Medical Emergencies.<br>➢ Competence in the ABCDE approach is mainly trained using simulation training, while little research has been done to analyse the effectiveness of simulation training in acquiring this structural approach.<br>**What this study adds?**<br>➢ This is the first study investigating the effectiveness of simulation training aimed at teaching doctors a systematic primary assessment using the ABCDE approach.<br>➢ This study demonstrated that simulation training is effective in teaching the ABCDE approach.<br>➢ Strikingly, analysis of all separate skills showed that communications kills deteriorate the most over time. |

For peer review only



For peer review only - http://bmjopen.bmj.com/site/about/guidelines.xhtml

# INTRODUCTION

## Background

In emergency medicine, assessing incoming patients in life threatening conditions following a structured approach is considered essential for successful resuscitation. A structured approach facilitates optimal use of time and resources for early recognition of deterioration, especially in the so-called 'golden hour', which refers to a time period of one hour or less following traumatic injury or medical emergency,[1, 2]. This golden hour during which resuscitation could be most beneficial has been recognized in several emergencies such as trauma, stroke, sepsis and shock,[3-5]. Using the structured approach “Airway-Breathing-Circulation-Disability-Exposure” (ABCDE) in the primary assessment for early recognition and immediate treatment of life threatening conditions has been the standard approach in trauma for decades and the use of the ABCDE primary assessment has also increased in other medical emergencies in recent years.

## Importance

Solid empirical evidence for the usefulness of the ABCDE approach and its clinical benefits to patients is limited,[5, 6]. Despite this, the Dutch inspection for healthcare (IGZ) requires that physicians treating non-trauma patients in the emergency department are ABCDE trained,[7].Therefore, completing an ABCDE course is mandatory for working on the ED. These courses usually contain lectures and simulation scenario training. Despite the wide use of simulation training for teaching the systematic ABCDE approach, little research has been done to analyse the effectiveness of simulation training in acquiring this structural approach. Simulation training has been proven to be effective for learning technical skills and maintaining skills that are not frequently used in daily practice, like airway management



For peer review only - http://bmjopen.bmj.com/site/about/guidelines.xhtml

and surgical skills,[8-10]. Simulation training can also improve communication, efficiency and safety during teamwork,[11-13]. A few studies based on self-perceptions showed that simulation training improved, participants confidence levels; they felt more competent in applying the ABCDE approach and several other skills,[14-16].

To our knowledge, it has not been investigated before whether simulation training actually improves the doctors' skills in performing this structured approach.

Our study focused on the effectiveness of simulation training for learning the systematic ABCDE approach. Our main goal was to analyse the short- and long-term effectiveness of simulation training to acquire a structured ABCDE approach. Furthermore, we wanted to analyse which skills and competences were acquired and maintained best.

## METHODS

### Study design

We conducted an interventional cohort study. The intervention consisted of a two day simulation-based ABCDE-teaching course for residents and non-residents. The measurements through video recordings were obtained before (T1), directly after (T2) and approximately three months after the intervention (T3).

Three simulation scenario's (I,II and III) with different medical emergencies were specifically designed for this study, but were of comparable difficulty as the course simulation scenario's. All simulations contained a life threatening condition in 3 of the 5 main items from the ABCDE to offer variable and realistic scenarios with the same degree of difficulty. The simulations were offered in random order, to prevent bias caused by the type or



For peer review only - http://bmjopen.bmj.com/site/about/guidelines.xhtml

difficulty of the simulation. This means that each participant received three different simulations and the three simulations were equally divided over the three different time measurements.

We developed an assessment form (Figure 1) to evaluate the participants' performance with regard to skills and competences essential to medical emergencies. The assessment form was divided in 5 categories according to the ABCDE structure. In each category, the skills or competences could be rated on a 2 (agree, not agree) or 5-point scale (agree, partially agree, partially not agree, not agree or does not apply).

**Intervention**

The ABCDE course is a  two-day course which exists for ten years now.  It includes mainly simulation training and two theoretical lectures about airway management and ALS. Previous to this course, the participants receive a book with mandatory chapters describing the ABCDE approach and various acute medical emergencies. The course encompasses 24 simulations with a patient simulator in which participants perform primary assessments of acute ill patients. In 8 scenarios, they participate in the role as a physician; in the other scenarios, they are in the role of "non-obstructing nurse" or observer.

The participants receive a certificate if they pass the theoretical test and if they are, according to the instructors, capable of performing a structured primary assessment of an acute ill patient, with recognition and resuscitation of life-threatening conditions and clear communication.

**Study setting and population**



For peer review only - http://bmjopen.bmj.com/site/about/guidelines.xhtml

We approached all participants of this two-day course to voluntarily participate in the study between August 2012 and March 2014.

The study participants were invited to act in a simulation as physician at three different moments, separate from the course. We instructed them to perform a primary assessment following the ABCDE approach.

This study used the patient simulator Laerdal Resusci Anne SkillTrainer with an upgrade Vitale Signs Sim Software Complete package, which is also used during the course. This simulator features heart and lung sounds, chest excursions, pulses and can show all vital signs on a separate monitor. With a separate computer, the sounds and vital signs can be changed during the scenario, to simulate several acute medical conditions.

During the video recordings, the simulator, materials and environment were the same as during the course.

**Study Protocol**

The first recording (T1) was taken 1-2 weeks prior to the course. The second recording (T2) was taken within 1 week after the course. The third and last recording (T3) was taken approximately three months after the course.

The research team consisted of 5 physicians, who were also course instructors. They were all instructed in detail to only facilitate the simulation and not help the participant in any way. Two emergency physicians, who were also course instructors but who were not involved in the recordings, independently rated the recorded primary assessments on the assessment form (Figure 1). The observers rated the videos in random order and were blinded to the measurement moment.



For peer review only - http://bmjopen.bmj.com/site/about/guidelines.xhtml

**Measurements**

The primary outcome of this study was the improvement in primary assessment scores of participants as a result of the ABCDE simulation training. We also investigated whether the skills acquired were maintained over a period of three months and which skills and competences were learned and maintained best.

**Data analysis**

To perform the statistical analysis, IBM SPPSS version 23.0 was used. In all analyses, a p-value of < 0.05 was regarded as significant.

Each skill or competence on the assessment form had a lowest score of 0 and a highest score of 1, so the weight of each item was the same, independent of the 2 or 5 point scale (agree=1, partially agree=0.67, partially not agree=0.33, not agree=0).

Because some skills or competences were marked as not applicable, we calculate mean scores in each category (A, B, C, D, E and remaining items). In each category the maximal score to obtain was 1. Therefore the maximal total score to obtain on the primary assessment was 6.

The interobserver reliability between the scores given by the two observers for the three different time measurements was calculated using the Spearman's rank correlation test (for T1, T2 and T3 (resp. R 0.81, 0.61, and 0.80). This interobserver reliability was generally high enough to average the mean scores of the two observers for each participant at T1, T2 and T3 for use in further analyses.



For peer review only - http://bmjopen.bmj.com/site/about/guidelines.xhtml

We used the Friedman test for three related samples to compare the mean rank total score of the whole group on the primary assessment at T1, T2 and T3.

We used the Wilcoxon signed rank test to compare the mean rank total scores on the primary assessment and to compare each scored item separately between T1 and T2, between T2 and T3 and between T1 and T3, based on the performance of each individual participant.

**RESULTS**

**Characteristics of study subjects**

We enrolled thirty participants in this study. Twenty-one were female, nine were male. Their mean age was 27 years (range 24-35, SD 2.77), their mean work experience was 11 months (range 0-48, SD14.4).

The video recording of T3 of one participant was last due to technical problems.

**Main results**

The mean total scores on the primary assessment of the whole group at T1, T2 and T3 were 2.90, 5.06, and 4.67 respectively (Table 1 and Figure 2).

Table 1: Total scores on the primary assessment of the whole group at T1, T2 and T3

| Time | Mean | SD | Median |
|---|---|---|---|
| 1 | 2.90 | 0.88 | 2.79 |
| 2 | 5.06 | 0.48 | 5.22 |
| 3 | 4.67 | 0.75 | 4.70 |



For peer review only - http://bmjopen.bmj.com/site/about/guidelines.xhtml

Friedmans test showed that the mean rank total scores of the whole group on the primary assessment at T1, T2 en T3 were 1.14, 2.62 and 2.24, respectively. Wilcoxon signed rank test showed that the mean rank total score on the primary assessment at T2 (directly after the course) was significantly higher than the mean rank total score at T1 (before the course, table 2). The mean rank total score on the primary assessment at T3 (three months after the course) was significantly lower than the mean rank total score at T2, but remained significantly higher than the mean rank total score at T1 (Table 2).

Table 2: Wilcoxon rank test for the mean rank total score on the primary assessment

| Difference | Significance |
|---|---|
| T1 < T2 | P = 0.000 |
| T2 > T3 | P = 0.035 |
| T3 > T1 | P = 0.000 |

The separate skills or competences were almost all scored significantly higher at T2 than at T1 (36 out of 41). Only the score on “resuscitates adequately in the E” did not differ significantly between T1 and T2. The other 4 skills could not be analysed as they were scored too often as “does not apply” (Table 3).

The separate skills most often showed no significant difference between T2 and T3 (30 out of 41). The skills (7 out of 41), “mentions deviating findings in the C”, “recognizes life threatening conditions in the C”, “ mentions conclusions in the C”, “examines D completely”, “communicates clearly”, “shows confidence” and “shows good leadership” were scored



For peer review only - http://bmjopen.bmj.com/site/about/guidelines.xhtml

significantly lower at T3 than at T2, but significant higher than at T1 (Table 4). Again, the other 4 skills could not be analysed as they were scored too often as "does not apply" (Table 3).

Table 3: Skills and competences that were not possible to analyse due to low numbers

| Skill/Competence |
|---|
| recognizes life-threatening conditions in the B |
| resuscitate adequately in the B |
| orders right additional diagnostics in the D |
| orders right additional diagnostics in the E |

Table 4: Wilcoxon signed rank test for separate skills and competences that decreased between T2 and T3

| Skill/Competence | Difference | Significance |
|---|---|---|
| mentions deviating findings in the C | T2 > T3 and T1 < T3 | P = 0.004 and P = 0.003 |
| recognizes live-threatening conditions in the C | T2 > T3 and T1 < T3 | P = 0.035 and P = 0.005 |
| mentions conclusions in the C | T2 > T3 and T1 < T3 | P = 0.044 and P = 0.002 |
| examines the D completely | T2 > T3 and T1 < T3 | P = 0.001 and P = 0.000 |
| communicates clearly | T2 > T3 and T1 < T3 | P = 0.010 and P = 0.000 |
| shows confidence | T2 > T3 and T1 < T3 | P = 0.019 and P = 0.000 |
| shows good leadership | T2 > T3 and T1 < T3 | P = 0.049 and P = 0.000 |



For peer review only - http://bmjopen.bmj.com/site/about/guidelines.xhtml

## DISCUSSION

Our study is the first to demonstrate the positive effect of teaching the ABCDE approach for medically ill patients using simulation. This effect persists even 3 months after completing the course although some decline in participant performance on the primary assessment was seen.

We found that most skills and competences are learned and maintained very well. Strikingly, most skills (five out of seven) that decrease over time are skills related to communication and leadership (Table 4). This is illustrated by a decrease in time of "recognition of life threatening conditions in the C", while the scores on the resuscitation skills did not decline. It is possible that the lower score reflect 'not thinking out loud' rather than failing to recognize a life threatening condition.

This decrease in communication suggests that focusing on communication and leadership by refresher courses or team training, after completing a simulation course, may be an important topic for physicians to maintain their skills. The positive effect of team training for these non-technical skills has already been shown,[11-13].

Another skill that does not yield scores as high as most other skills after three months, is a complete examination of the Disability. A possible explanation for this finding is that the participants decide on the level of consciousness of the patient, determined by the EMV (Glascow coma score), whether it is necessary to examine certain components of the Disability. The performance of the EMV does not decrease over time. This finding is in line



For peer review only - http://bmjopen.bmj.com/site/about/guidelines.xhtml

with previous research from our group on primary assessment completeness showing that during the primary assessment in the emergency department, residents and experienced staff have equal, but not maximum ABCDE completeness scores (83 instead of 100),[17]. This outcome may reflect that a score of 100 is not necessary to exclude potential life threatening diseases or stabilize the patients.

**Limitations**

It was not possible to assess all specific skills in each simulation due to reality of the scenario. A few skills were often scored as “does not apply”, which rendered the number of observations for those skills too few to analyse reliable differences. This was probably caused by the fact that scenarios contained a life threatening condition only in 3 of the 5 main items from the ABCDE.

We think that this limitation wasn’t of any influence on the results because when an item is scored often as “does not apply” it doesn’t help to discriminate in quality of performance. Finally, the participants knew they were a study subject. We do not know if they have prepared for the study scenario. This might have influenced the study scores at T1 and T3.

**Conclusion**

Simulation training is an effective educational tool to teach doctors to perform a structured primary assessment using the ABCDE. Communication skills tend to decrease over time, so it will be useful to organize refresher courses, team training or another kind of training with a focus on communication and leadership.

For peer review only



For peer review only - http://bmjopen.bmj.com/site/about/guidelines.xhtml

**Acknowledgements**

We want to thank Kinge van der Heide for her help with the recordings. We want to thank Mirjam Doff-Holman and Martine Oosterloo for the assessment of all recordings.

For peer review only

**REFERENCES**

1. Franklin C, Mathew J. Developing strategies to prevent inhospital cardiac arrest: analyzing responses of physicians and nurses in the hours before the event. *Crit Care Med* 1994;22:244-7.

2. McQuillan P, Pilkington S, Allan A, Taylor B, Short A, Morgan G, et al. Confidential inquiry into quality of care before admission to intensive care. *BMJ* 1998;316:1853-8.

3. Kumar A, Roberts D, Wood KE, Light B, Parrillo JE, Sharma S, et al. Duration of hypotension before initiation of effective antimicrobial therapy is the critical determinant of survival in human septic shock. *Crit Care Med* 2006;34:1589-96.

4. National Institute of Neurological Disorders and Stroke rt-PA Stroke Study Group. Tissue plasminogen activator for acute ischemic stroke. *N Engl J Med* 1995;333:1581-7.

5. Soreide K. Three decades (1978-2008) of Advanced Trauma Life Support (ATLS) practice revised and evidence revisited. *Scand J Trauma Resusc Emerg Med* 2008;16:19,7241-16-19.

6. Jayaraman S, Sethi D, Chinnock P, Wong R. Advanced trauma life support training for hospital staff. *Cochrane Database Syst Rev* 2014;(8):CD004173. doi:CD004173.



For peer review only - http://bmjopen.bmj.com/site/about/guidelines.xhtml

7. Spoedeisende hulp: vanuit een stevige basis. Rapportage werkgroep levelindeling SEH. 2009 https://www.netwerkacutezorgnoordwest.nl/app/uploads/2017/01/200910-Rapport-SEH-Vanuit-een-stevig-basis.pdf.

8. Ryu WHA, Dharampal N, Mostafa AE, Sharlin E, Kopp G, Jacobs WB, et al. Systematic Review of Patient-Specific Surgical Simulation: Toward Advancing Medical Education. *J Surg Educ* 2017.

9. Price J, Naik V, Boodhwani M, Brandys T, Hendry P, Lam BK. A randomized evaluation of simulation training on performance of vascular anastomosis on a high-fidelity in vivo model: the role of deliberate practice. *J Thorac Cardiovasc Surg* 2011;142:496-503.

10. Kory PD, Eisen LA, Adachi M, Ribaudo VA, Rosenthal ME, Mayo PH. Initial airway management skills of senior residents: simulation training compared with traditional training. *Chest* 2007;132:1927-31.

11. Buljac-Samardzic M, Dekker-van Doorn CM, van Wijngaarden JD, van Wijk KP. Interventions to improve team effectiveness: a systematic review. *Health Policy* 2010;94:183-95.

12. Hesselink G, Berben S, Beune T, Schoonhoven L. Improving the governance of patient safety in emergency care: a systematic review of interventions. *BMJ Open* 2016;6:e009837,2015-009837.

13. Shapiro MJ, Morey JC, Small SD, Langford V, Kaylor CJ, Jagminas L, et al. Simulation based teamwork training for emergency department staff: does it improve clinical team performance when added to an existing didactic teamwork curriculum?. *Qual Saf Health Care* 2004;13:417-21.



For peer review only - http://bmjopen.bmj.com/site/about/guidelines.xhtml

14. Willett TG, Kirlew M, Cardinal P, Karas P. An evaluation of the Acute Critical Events Simulation (ACES) course for family medicine residents. *Can J Rural Med* 2011;16:89-95.

15. Carling J. Are graduate doctors adequately prepared to manage acutely unwell patients?. *Clin Teach* 2010;7:102-5.

16. Pantelidis P, Staikoglou N, Paparoidamis G, Drosos C, Karamaroudis S, Samara A, et al. Medical students' satisfaction with the Applied Basic Clinical Seminar with Scenarios for Students, a novel simulation-based learning method in Greece. *J Educ Eval Health Prof* 2016;13:13.

17. Olgers TJ, Dijkstra RS, Drost-de Klerck AM, Ter Maaten JC. The ABCDE primary assessment in the emergency department in medically ill patients: an observational pilot study. *Neth J Med* 2017;75:106-11.

For peer review only

# Figure 1: ABCDE Assessment Form

| Primary assessment | | |
|---|---|---|
| A | - examines the airway<br>- mentions threatened airway<br>- applies airway maneuvers<br>- applied oxygen | agree / disagree<br>agree / disagree/ d.n.a.<br>agree / partially agree / partially disagree / disagree / d.n.a.<br>agree / disagree/ d.n.a. |
| B | - examines B completely (color, trachea, resp. rate, excursions, accessory muscle, percussion, auscultation, saturation<br>- gives nurse the right orders<br>- mentions deviating findings<br>- recognizes life-threatening conditions<br>- orders right additional diagnostics<br>- mentions conclusions/interpretation<br>- resuscitate adequately | 1 - 2 - 3 - 4 - 5 - 6 - 7 – 8<br>agree / partially agree / partially disagree / disagree / d.n.a.<br>agree / partially agree / partially disagree / disagree / d.n.a.<br>agree / disagree / d.n.a.<br>agree / disagree / d.n.a.<br>agree / partially agree / partially disagree / disagree<br>agree / partially agree / partially disagree / disagree / d.n.a. |
| C | - examines C completely (acra, central pulse, heartrate, bloodpressure, cap.refill, CVD, heartsounds)<br>- gives nurse the right orders<br>- mentions deviating findings<br>- recognizes life-threatening conditions<br>- orders right additional diagnostics<br>- mentions conclusions/interpretation<br>- resuscitate adequately | 1 - 2 - 3 - 4 - 5 - 6 - 7<br>agree / partially agree / partially disagree / disagree<br>agree / partially agree / partially disagree / disagree<br>agree / disagree / d.n.a.<br>agree / disagree / d.n.a.<br>agree / partially agree / partially disagree / disagree<br>agree / partially agree / partially disagree / disagree / d.n.a. |
| D | - examines D completely (EMV, pupils, meningitis, glucose)<br>- applies EMC correctly<br>- mentions deviating findings<br>- recognizes life-threatening conditions<br>- orders right additional diagnostics<br>- mentions conclusions/interpretation<br>- resuscitate adequately | 1 - 2 - 3 – 4<br>agree / partially agree / partially disagree / disagree<br>agree / partially agree / partially disagree / disagree/ d.n.a.<br>agree / disagree / d.n.a.<br>agree / disagree / d.n.a.<br>agree / partially agree / partially disagree / disagree<br>agree / partially agree / partially disagree / disagree / d.n.a. |
| E | - examines E completely (temperature, head-to toe)<br>- gives nurse the right orders<br>- mentions deviating findings<br>- orders right additional diagnostics<br>- mentions conclusions/interpretation<br>- resuscitate adequately | 1 – 2<br>agree / partially agree / partially disagree / disagree/ d.n.a.<br>agree / partially agree / partially disagree / disagree/ d.n.a.<br>agree / disagree / d.n.a.<br>agree / partially agree / partially disagree / disagree<br>agree / partially agree / partially disagree / disagree / d.n.a. |
| R<br>E<br>M<br>A<br>I<br>N<br>I<br>N<br>G | - adequately asks for help<br>- communicates clearly<br>- summarizes adequately<br>- draws the right conclusions<br>- clinical reasoning is adequately<br>- works structured<br>- stays calm<br>- shows confidence<br>- shows good leadership | agree / partially agree / partially disagree / disagree<br>agree / partially agree / partially disagree / disagree<br>agree / partially agree / partially disagree / disagree<br>agree / partially agree / partially disagree / disagree<br>agree / partially agree / partially disagree / disagree<br>agree / partially agree / partially disagree / disagree<br>agree / partially agree / partially disagree / disagree<br>agree / partially agree / partially disagree / disagree<br>agree / partially agree / partially disagree / disagree |

For peer review only

Figure 2: Boxplot comparing Total score on primary assessment at T1, T2 and T3.

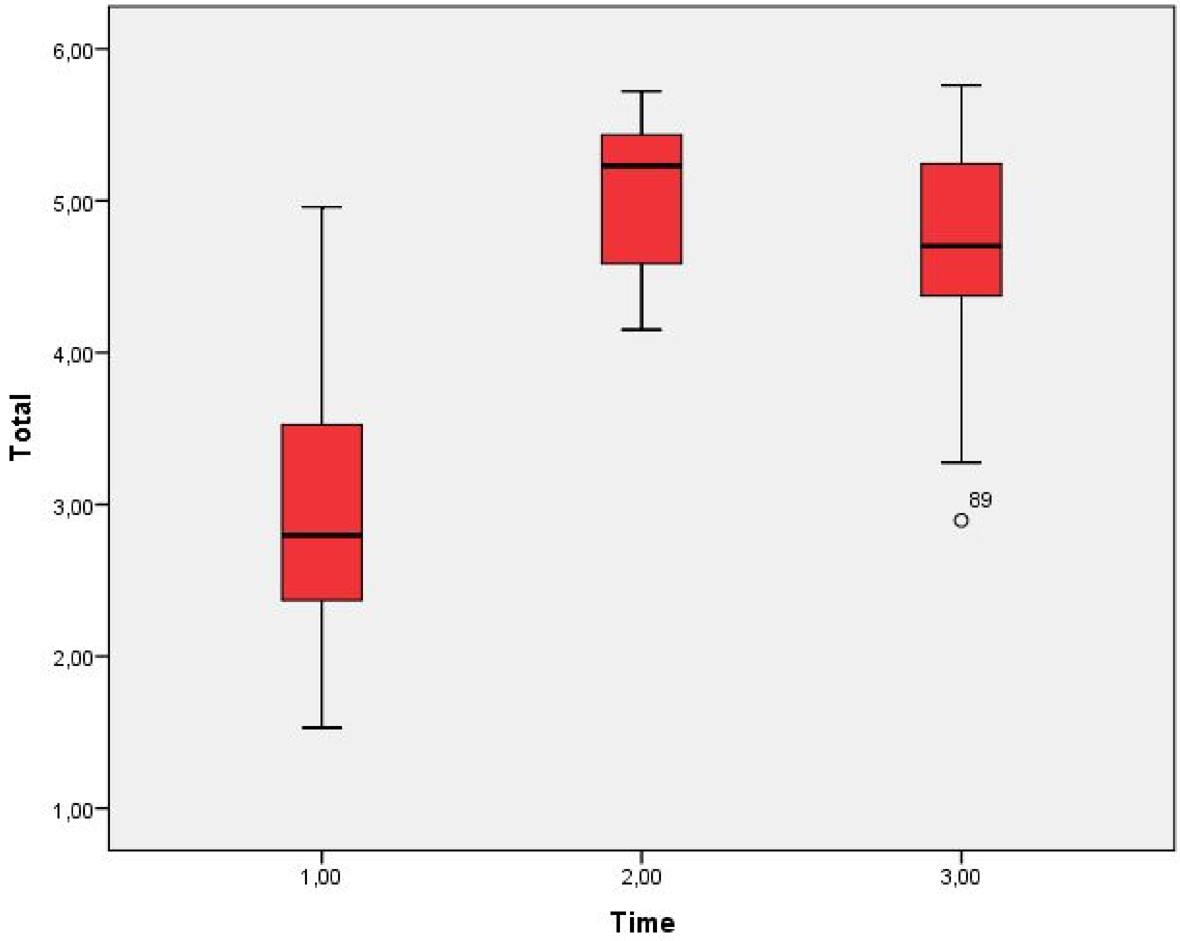


w only

Supplemental file: Wilcoxon signed rank test for all separate skills and competences

| Wilcoxon | | |
|---|---|---|
| Skill/Competence | Difference | Significance |
| examines the airway | T1<T2, T2=T3 and T1=T3 | p=0.034,p=0.157 and p=0.206 |
| mentions threatened airway | T1<T2, T2=T3 and T1<T3 | p=0.008, p=1.000 and p=0.007 |
| applies airway maneuvers | T1<T2, T2=T3 and T1<T3 | p=0.000, p=0.281 and p=0.001 |
| applies oxygen | T1<T2, T2=T3 and T1<T3 | p=0.006, p=0.739 and p=0.001 |
| examines B completely | T1<T2, T2=T3 and T1<T3 | p=0.001, p=0.210 and p=0.015 |
| gives nurse the right orders in the B | T1<T2, T2=T3 and T1<T3 | p=0.003, p=0.317 and p=0.003 |
| mentions deviating findings in the B | T1<T2, T2=T3 and T1<T3 | p=0.000, p=0.119 and p=0.002 |
| recognizes life-threatening conditions in B | T1<T2, T2=T3 and T1<T3 | N= to low |
| orders right additional diagnostics in the B | T1<T2, T2=T3 and T1<T3 | p=0.020 (n=15), p=1.000 (N=13) and p=0.031 N=14 |
| mentions conclusions/interpretation in the B | T1<T2, T2=T3 and T1<T3 | p=0.000, p=0.192 and p=0.000 |
| resuscitate adequately in the B | T1<T2, T2=T3 and T1<T3 | N= to low |
| examines C completely | T1<T2, T2=T3 and T1<T3 | p=0.000, p=0.774 and p=0.001 |
| gives nurse the right orders in the C | T1<T2, T2=T3 and T1<T3 | p=0.000, p=0.381 and p=0.000 |
| mentions deviating findings in the C | T1<T2, T2>T3 and T1<T3 | p=0.000, p=0.004 and p=0.003 |
| recognizes life-threatening conditions in C | T1<T2, T2>T3 and T1<T3 | p=0.000, p=0.035 and p=0.005 |
| orders right additional diagnostics in the C | T1<T2, T2=T3 and T1=T3 | p=0.016, p=0.083 and p=0.052 |
| mentions conclusions/interpretation in the C | T1<T2, T2>T3 and T1<T3 | p=0.000, p=0.044 and p=0.002 |
| resuscitate adequately in the C | T1<T2, T2=T3 and T1<T3 | p=0.000, p=0.637 and p=0.002 |
| examines D completely | T1<T2, T2>T3 and T1<T3 | p=0.000, p=0.001 and p=0.000 |
| applies EMV (Glascow coma score) correctly | T1<T2, T2=T3 and T1<T3 | p=0.000, p=0.200 and p=0.021 |
| mentions deviating findings in the D | T1<T2, T2=T3 and T1<T3 | p=0.000, p=0.117 and p=0.001 |
| recognizes life-threatening conditions in D | T1<T2, T2=T3 and T1<T3 | p=0.000, p=0.108 and p=0.008 |
| orders right additional diagnostics in the D | T1<T2, T2=T3 and T1<T3 | N=to low |
| mentions conclusions/interpretation in the D | T1<T2, T2=T3 and T1<T3 | p=0.000, p=0.141 and p=0.000 |
| resuscitate adequately in the D | T1<T2, T2=T3 and T1<T3 | p=0.000, p=0.230 and p=0.001 |
| examines E completely | T1<T2, T2=T3 and T1<T3 | p=0.003,p=0.465 and p=0.015 |
| gives nurse the right orders in the E | T1<T2, T2=T3 and T1<T3 | p=0.000,p=0.059 and p=0.000 |
| mentions deviating findings in the E | T1<T2, T2=T3 and T1<T3 | p=0.000, p=0.360 and p=0.000 |
| orders right additional diagnostics in the E | T1<T2, T2=T3 and T1<T3 | p=0.564 (N=5), N= to low |

For peer review only

| | | |
|---|---|---|
| mentions conclusions/interpretation in the E | T1<T2, T2=T3 and T1<T3 | p=0.000, p=0.245 and p=0.000 |
| resuscitate adequately in the E | T1<T2, T2=T3 and T1<T3 | p=0.10 (N=13), p=0.139 (N=11), p=0.324 (N=13) |
| adequately asks for help | T1<T2, T2=T3 and T1<T3 | p=0.005, p=0.768 and p=0.039 |
| communicates clearly | T1<T2, T2>T3 and T1<T3 | p=0.000, p=0.010 and p=0.000 |
| summarizes adequately | T1<T2, T2=T3 and T1<T3 | p=0.000, p=0.062 and p=0.000 |
| draws the right conclusions | T1<T2, T2=T3 and T1<T3 | p=0.000, p=0.527 and p=0.000 |
| clinical reasoning is adequately | T1<T2, T2=T3 and T1<T3 | p=0.000, p=0.398 and p=0.000 |
| works structured | T1<T2, T2=T3 and T1<T3 | p=0.000, p=0.490 and p=0.000 |
| stays calm | T1<T2, T2=T3 and T1<T3 | p=0.000, p=1.000 and p=0.000 |
| shows confidence | T1<T2, T2>T3 and T1<T3 | p=0.000, p=0.010 and p=0.000 |
| shows good leadership | T1<T2, T2>T3 and T1<T3 | p=0.000, p=0.019 and p=0.000 |

For peer review only

STROBE Statement—checklist of items that should be included in reports of observational studies

| | Item No | Recommendation |
|---|---|---|
| **Title and abstract** | 1 | (*a*) Indicate the study's design with a commonly used term in the title or the abstract **Page 1** |
| | | (*b*) Provide in the abstract an informative and balanced summary of what was done and what was found **Page 4** |
| **Introduction** | | |
| Background/rationale | 2 | Explain the scientific background and rationale for the investigation being reported **Page 7 and 8** |
| Objectives | 3 | State specific objectives, including any prespecified hypotheses **Page 8** |
| **Methods** | | |
| Study design | 4 | Present key elements of study design early in the paper **Page 8-9** |
| Setting | 5 | Describe the setting, locations, and relevant dates, including periods of recruitment, exposure, follow-up, and data collection **Page 9-10** |
| Participants | 6 | (*a*) *Cohort study*—Give the eligibility criteria, and the sources and methods of selection of participants. Describe methods of follow-up **Page 9**<br>*Case-control study*—Give the eligibility criteria, and the sources and methods of case ascertainment and control selection. Give the rationale for the choice of cases and controls<br>*Cross-sectional study*—Give the eligibility criteria, and the sources and methods of selection of participants |
| | | (*b*) *Cohort study*—For matched studies, give matching criteria and number of exposed and unexposed<br>*Case-control study*—For matched studies, give matching criteria and the number of controls per case |
| Variables | 7 | Clearly define all outcomes, exposures, predictors, potential confounders, and effect modifiers. Give diagnostic criteria, if applicable **Page 8-10** |
| Data sources/ measurement | 8* | For each variable of interest, give sources of data and details of methods of assessment (measurement). Describe comparability of assessment methods if there is more than one group **Page 11-12** |
| Bias | 9 | Describe any efforts to address potential sources of bias **Page 8-11** |
| Study size | 10 | Explain how the study size was arrived at **(no effectsize known)** |
| Quantitative variables | 11 | Explain how quantitative variables were handled in the analyses. If applicable, describe which groupings were chosen and why |
| Statistical methods | 12 | (*a*) Describe all statistical methods, including those used to control for confounding **Page 11-12** |
| | | (*b*) Describe any methods used to examine subgroups and interactions |
| | | (*c*) Explain how missing data were addressed **Page 11** |
| | | (*d*) *Cohort study*—If applicable, explain how loss to follow-up was addressed<br>*Case-control study*—If applicable, explain how matching of cases and controls was addressed<br>*Cross-sectional study*—If applicable, describe analytical methods taking account of sampling strategy |

For peer review only

(*e*) Describe any sensitivity analyses **Page 11 (interobserver reliability)**

For peer review only



For peer review only - http://bmjopen.bmj.com/site/about/guidelines.xhtml

| | | |
|---|---|---|
| **Results** | | |
| Participants | 13* | (a) Report numbers of individuals at each stage of study—eg numbers potentially eligible, examined for eligibility, confirmed eligible, included in the study, completing follow-up, and analysed **Page 12** |
| | | (b) Give reasons for non-participation at each stage |
| | | (c) Consider use of a flow diagram |
| Descriptive data | 14* | (a) Give characteristics of study participants (eg demographic, clinical, social) and information on exposures and potential confounders **Page 11** |
| | | (b) Indicate number of participants with missing data for each variable of interest **Page 12 and supplemental file** |
| | | (c) *Cohort study*—Summarise follow-up time (eg, average and total amount) |
| Outcome data | 15* | *Cohort study*—Report numbers of outcome events or summary measures over time **Page 12-14** |
| | | *Case-control study*—Report numbers in each exposure category, or summary measures of exposure |
| | | *Cross-sectional study*—Report numbers of outcome events or summary measures |
| Main results | 16 | (*a*) Give unadjusted estimates and, if applicable, confounder-adjusted estimates and their precision (eg, 95% confidence interval). Make clear which confounders were adjusted for and why they were included **Page 12-14, Table 1-4** |
| | | (*b*) Report category boundaries when continuous variables were categorized |
| | | (*c*) If relevant, consider translating estimates of relative risk into absolute risk for a meaningful time period |
| Other analyses | 17 | Report other analyses done—eg analyses of subgroups and interactions, and sensitivity analyses **Page 11 (interobserver reliability)** |
| **Discussion** | | |
| Key results | 18 | Summarise key results with reference to study objectives **Page 15-16** |
| Limitations | 19 | Discuss limitations of the study, taking into account sources of potential bias or imprecision. Discuss both direction and magnitude of any potential bias **Page 16** |
| Interpretation | 20 | Give a cautious overall interpretation of results considering objectives, limitations, multiplicity of analyses, results from similar studies, and other relevant evidence **Page 15-16** |
| Generalisability | 21 | Discuss the generalisability (external validity) of the study results **Page 16** |
| **Other information** | | |
| Funding | 22 | Give the source of funding and the role of the funders for the present study and, if applicable, for the original study on which the present article is based **Page 3** |

*Give information separately for cases and controls in case-control studies and, if applicable, for exposed and unexposed groups in cohort and cross-sectional studies.



For peer review only - http://bmjopen.bmj.com/site/about/guidelines.xhtml

**Note:** An Explanation and Elaboration article discusses each checklist item and gives methodological background and published examples of transparent reporting. The STROBE checklist is best used in conjunction with this article (freely available on the Web sites of PLoS Medicine at http://www.plosmedicine.org/, Annals of Internal Medicine at http://www.annals.org/, and Epidemiology at http://www.epidem.com/). Information on the STROBE Initiative is available at www.strobe-statement.org.

For peer review only


For peer review only - http://bmjopen.bmj.com/site/about/guidelines.xhtml

# Use of simulation training to teach the ABCDE primary assessment: an observational study in a Dutch University Hospital with a 3-months follow up.


| | |
|---|---|
| Journal: | *BMJ Open* |
| Manuscript ID | bmjopen-2019-032023.R1 |
| Article Type: | Original research |
| Date Submitted by the Author: | 18-Sep-2019 |


| | |
|---|---|
| Complete List of Authors: | Drost- de Klerck, Amanda; Universitair Medisch Centrum Groningen, emergency department<br>Olgers, Tycho; University Medical Center Groningen, Internal medicine, Emergency dept.<br>van de Meeberg, Evelien; Universitair Medisch Centrum Groningen, emergency department<br>Schonrock-Adema, Johanna; Universitair Medisch Centrum Groningen, emergency department<br>ter Maaten, Jan; University Medical Center Groningen, Emergency Department, Department of internal medicine |


| | |
|---|---|
| <b>Primary Subject Heading</b>: | Medical education and training |
| Secondary Subject Heading: | Emergency medicine |
| Keywords: | ACCIDENT & EMERGENCY MEDICINE, EDUCATION & TRAINING (see Medical Education & Training), GENERAL MEDICINE (see Internal Medicine), MEDICAL EDUCATION & TRAINING |
| | |

*I, the Submitting Author has the right to grant and does grant on behalf of all authors of the Work (as defined in the below author licence), an exclusive licence and/or a non-exclusive licence for contributions from authors who are: i) UK Crown employees; ii) where BMJ has agreed a CC-BY licence shall apply, and/or iii) in accordance with the terms applicable for US Federal Government officers or employees acting as part of their official duties; on a worldwide, perpetual, irrevocable, royalty-free basis to BMJ Publishing Group Ltd ("BMJ") its licensees and where the relevant Journal is co-owned by BMJ to the co-owners of the Journal, to publish the Work in this journal and any other BMJ products and to exploit all rights, as set out in our licence.*

*The Submitting Author accepts and understands that any supply made under these terms is made by BMJ to the Submitting Author unless you are acting as an employee on behalf of your employer or a postgraduate student of an affiliated institution which is paying any applicable article publishing charge ("APC") for Open Access articles. Where the Submitting Author wishes to make the Work available on an Open Access basis (and intends to pay the relevant APC), the terms of reuse of such Open Access shall be governed by a Creative Commons licence – details of these licences and which Creative Commons licence will apply to this Work are set out in our licence referred to above.*

*Other than as permitted in any relevant BMJ Author's Self Archiving Policies, I confirm this Work has not been accepted for publication elsewhere, is not being considered for publication elsewhere and does not duplicate material already published. I confirm all authors consent to publication of this Work and authorise the granting of this licence.*

For peer review only

# Use of simulation training to teach the ABCDE primary assessment: an observational study in a Dutch University Hospital with a 3-months follow up.

*Amanda M Drost-de Klerck, Tycho J Olgers, Evelien K van de Meeberg, Johanna Schönrock-Adema, Jan C ter Maaten.*

*Emergency Department, University Medical Center Groningen, Hanzeplein 1, 9713 GZ Groningen, The Netherlands, Amanda M Drost-de Klerck, Consultant Emergency Medicine. Emergency Department, University Medical Center Groningen, Tycho J Olgers, Consultant Acute Internal Medicine. Emergency Department, University Medical Center Groningen, Evelien K van de Meeberg, Consultant Emergency Medicine. University of Groningen and University Medical Center Groningen, Institute for Medical Education, Johanna Schönrock-Adema, senior researcher. Emergency Department, University Medical Center Groningen, Jan C ter Maaten, Full Professor Acute Internal Medicine.*

**Word count:** 3116

**Address for correspondence/request for reprints:**

A.M. Drost-de Klerck

Phone: +31503616161 / Fax: +31503611725

Email: a.drost@umcg.nl

For peer review only

**Contributors**

AMDK and JCM have conceived the study and created the assessment form. AMDK, TJO, EKM and JCM collected the data. AMDK is guarantor and did the literature search, managed the data and performed data analysis and drafted the manuscript. JSA advised regarding to the data analysis. All authors contributed to the final manuscript, revision of the manuscript and final approval of the manuscript. The corresponding author attests that all listed authors meet authorship criteria and that no others meeting the criteria have been omitted.

**Copyright/license for publication**

The corresponding author has the right to grant on behalf of all authors and does grant on behalf of all authors, a worldwide licence to the Publishers and its licensees in perpetuity, in all forms, formats and media (whether known now or created in the future), to i) publish, reproduce, distribute, display and store the Contribution, ii) translate the Contribution into other languages, create adaptations, reprints, include within collections and create summaries, extracts and/or, abstracts of the Contribution, iii) create any other derivative work(s) based on the Contribution, iv) to exploit all subsidiary rights in the Contribution, v) the inclusion of electronic links from the Contribution to third party material where-ever it may be located; and, vi) licence any third party to do any or all of the above."

**Competing interests**

All authors have completed the ICMJE uniform disclosure form at www.icmje.org/coi_disclosure.pdf and declare: no support from any organisation for the

For peer review only

submitted work; no financial relationships with any organisations that might have an interest in the submitted work in the previous three years; no other relationships or activities that could appear to have influenced the submitted work.

**Ethics approval**

Ethics approval was not required because the study participants had signed up for a two-day course, the intervention, before they were asked to participate in the study. So, the intervention was not on behalf of the study.

**Transparency**

We declare that the manuscript is honest, accurate and transparent. No important aspects of the study have been omitted and discrepancies from the study as originally planned have been explained.

**Funding**

The study was only funded by the Emergency Department of the University Medical Center Groningen.

For peer review only

## ABSTRACT

**Objectives** To investigate short- and long-term effectiveness of simulation training to acquire a structured Airway Breathing Circulation Disability Exposure (ABCDE) approach for medical emergencies; and to examine which skills were learned and maintained best.

**Design** an observational intervention study with a 3-months follow-up.

**Setting** Skills centre of the University Medical Center Groningen.

**Participants** Thirty voluntary participants (mean age 27 years, 21 females, 9 males) of a simulation-based course.

**Intervention** A two-day ABCDE-teaching course for residents and non-residents. The course encompasses 24 simulations in which participants perform primary assessments of acute ill patients. Video recordings were taken of each participant performing a primary assessment, before (T1), directly after (T2) and three months after the intervention (T3).

**Main outcome measures** Physicians' performance in the ABCDE primary assessment at T1, T2 and T3. Two observers scored the primary assessments, blinded to measurement moment, using an assessment form to evaluate the performance with regard to skills essential for a structured ABCDE approach. The Friedman and Wilcoxon signed-rank test were used to compare physicians' performances on the subsequent measurement moments.

**Results** The mean rank scores on the primary assessment at T1, T2 and T3 were 1.14, 2.62 and 2.24, respectively (Table 1), and were significantly different, ($p< 0.001$).

The mean rank scores on the total primary assessment directly after the course (T2 vs T1 $p<0.001$) and three months after the course (T3 vs T1 $p<0.001$) were significantly better than before the course. Certain skills deteriorated during the three months follow-up. Strikingly, most skills that decrease over time are Crew Resources Management (CRM)-skills.


For peer review only

## Conclusion

A course using simulation training is an effective educational tool to teach physicians the ABCDE primary assessment. Certain CRM-skills decrease over time, so we recommend organizing refresher courses, simulation team training or another kind of simulation training with a focus on CRM-skills.

**Strengths and limitations of this study**

**Strengths**

- This is an observational intervention study measuring the 3-months effect of a simulation course.
- This study used the same environment for intervention and study.
- The observers were blinded to measurement moment during this study on participants' performance on the primary assessment using the ABCDE approach.

**Limitations**

- The fact that the observers might have been also the instructor of some study participants, may have influenced their ratings for a few participants, but potential bias was minimized by offering the videos in random order and blinded to measurement moment and our study focused on the outcomes at group level and not individual outcomes.
- There might be bias in the fact that we do not know if the participants have prepared for the study scenario at T1 and T3 (the Hawthorne effect), whereas preparation for T3 might result in an overestimation of the actual course effectiveness at follow-up, preparation at T1 might result in an underestimation of the effectiveness of the course on both T2 and T3.

For peer review only

## INTRODUCTION

### Background

In emergency medicine, assessing incoming patients in life-threatening conditions according to a structured approach is considered essential for successful resuscitation. The most widely used structured approach for early recognition and immediate treatment of life-threatening conditions is the "Airway-Breathing-Circulation-Disability-Exposure" (ABCDE) approach. The ABCDE approach is taught in the Advanced Trauma Life Support (ATLS) since 1978 and has been the standard approach in trauma since [1-3].

The use of the ABCDE primary assessment has also increased in other medical emergencies in recent years [4-7].

Using the ABCDE approach likely improves outcomes by helping health care professionals focus on the most life-threatening clinical problems and perform immediate resuscitation. Although solid empirical evidence for the usefulness of the ABCDE approach and its clinical benefits to patients is limited[1, 2], the importance of early treatment in the so-called 'golden hour' – which refers to a time period of one hour or less following traumatic injury or medical emergency during which resuscitation could be most beneficial – has been recognized in several emergencies such as trauma, stroke, sepsis and shock [1-5, 8-11].

### Importance

The Dutch inspection for healthcare (IGZ) requires that physicians treating non-trauma patients in the emergency department are ABCDE qualified [12]. Therefore, completing an ABCDE course is mandatory for physicians who work at the Emergency Department (ED). These courses usually contain lectures and simulation scenario training. Despite the wide use of simulation training for teaching the systematic ABCDE approach, little research has



For peer review only - http://bmjopen.bmj.com/site/about/guidelines.xhtml

been done to analyse the effectiveness of simulation training in acquiring this structural approach. Simulation training has been proven to be effective for learning technical skills and maintaining skills that are not frequently used in daily practice, like airway management and surgical skills [13-15]. Simulation training can also improve communication, efficiency and safety during teamwork [16-18]. A few studies based on self-perceptions showed that simulation training improved participants' confidence levels; they felt more competent in applying the ABCDE approach and several other skills [3, 19-21].

To our knowledge, it has not been investigated before whether simulation training actually improves physicians' skills in performing the structured ABCDE approach.

Our study focused on the effectiveness of simulation training to acquire a structured ABCDE approach. Our main goal was to analyse the short- and long-term effectiveness of simulation training to acquire a structured ABCDE approach. We analysed the improvement in physicians' primary assessment scores as a result of the ABCDE simulation training.

We also investigated whether the skills acquired were maintained over a period of three months and which skills and competences were learned and maintained best.

## METHODS

### Study design

We conducted an observational intervention study. The intervention consisted of a two-day simulation-based ABCDE teaching course. The measurements through video recordings were obtained before (T1), directly after (T2) and approximately three months after the intervention (T3).



For peer review only - http://bmjopen.bmj.com/site/about/guidelines.xhtml

Three simulation scenarios (I, II and III) with different medical emergencies were specifically designed for this study. Scenario I was a case with pneumosepsis and hypoglycaemia, a partially obstructed airway due to low consciousness and shock. Scenario II concerned a case with obstructive shock caused by pulmonary embolism and an opioid overdose with altered consciousness. Scenario III was a case with meningococcal sepsis with a partially obstructed airway due to low consciousness, bronchospasm and shock. We have designed three different and realistic scenarios with comparable difficulty by creating a life-threatening condition which needs resuscitation in three of the five main items from the ABCDE.

To prevent bias caused by the type or difficulty of the simulation, we varied the order in which participants had to complete the three simulation scenarios in such a way that the different scenario sequences were equally divided over T1, T2 and T3 (participant 1: T1 scenario A, T2 scenario B, T3 scenario C; participant 2: T1 scenario B, T2 scenario C, T3 scenario A; participant 3: T1 scenario C, T2 scenario A, T3 scenario B, etcetera). We made a schedule in which the order of the scenarios was prescribed for each participant and participants were divided over the schedule in order of inclusion.

We developed an assessment form (Figure 1) to evaluate the participants' performance regarding skills and competences essential to assess medical emergencies. The assessment form was divided in six categories; five concerned the ABCDE structure and the sixth one contained remaining items. The remaining items focus on some Crew Resources Management (CRM) skills, like collaboration, communication, acknowledge own boundaries, and leadership. In each category, the skills or competences could be rated on a two- (agree, not agree, does not apply) or four-point scale (agree, partially agree, partially not agree, not agree or does not apply). We have added the option “does not apply”, because some skills were not required in some simulation scenarios. In the categories B, C, D and E also the



For peer review only - http://bmjopen.bmj.com/site/about/guidelines.xhtml

number of examined items during the physical examination were scored. The following items could be scored; in the B: skin color, trachea position, respiratory rate, thorax excursions, breathing effort, lung percussion, lung auscultation and saturation; in the C: circulation of extremities, central pulse, heart rate, blood pressure, capillary refill, central venous pressure, heart sounds; in the D: Glasgow Coma Scale (EMV), pupils, neck stiffness, glucose; in the E: temperature, head to toe examination (Figure 1).

**Intervention**

The ABCDE course is a two-day course for non-residents and first year residents which exists for ten years now. For most participants, it is a mandatory course that they need to pass before they are allowed to work in the ED. The course consists mainly of simulation training and two theoretical lectures about airway management and Advanced Life Support (ALS). Previous to this course, the participants receive a book with chapters describing the ABCDE approach and various acute medical emergencies.

The course focuses on learning to recognize and treat life-threatening conditions, but also pays attention to some CRM-skills necessary for an efficient ABCDE approach.

This course is given in the skills centre in a room similar to a resuscitation room in the ED. The patient simulator used is a Laerdal Resusci Anne SkillTrainer with an upgrade Vitale Signs Sim Software Complete package. This simulator features heart and lung sounds, chest excursions, pulse and can show all vital signs on a separate monitor. With a separate computer, the sounds and vital signs can be changed during the scenario, to simulate several acute medical conditions.

For peer review only

Each course group consists of six participants and two instructors. During the simulation rounds the group is split in half and two scenarios are run simultaneously in two separate rooms.

The course encompasses a total of 24 simulations with a patient simulator in which participants perform the primary assessment of acute ill patients. In each scenario, the role of physician, “non-obstructive nurse” and observer are assigned to the three participants. One of the instructors operates the simulator and leads the debriefing afterwards.

In eight scenarios, the participants fulfil the role of physician; in the other scenarios, they take up the role of “non-obstructing nurse” or observer.

The participants receive a certificate if they pass the theoretical test and if they are, according to the instructors, capable of performing a structured primary assessment of an acute ill patient, with recognition and resuscitation of life-threatening conditions and adequate CRM-skills.

All course instructors are experts in the field of acute medicine, and experienced and certified course instructors.

**Study setting and population**

This study was conducted in the same skills centre as were the course took place. During the video recordings, the simulator, materials and environment were also the same as during the course.

We approached all participants prior to this two-day course by e-mail and invited them to participate voluntarily in the study between August 2012 and December 2013. We endeavoured to achieve a save response environment by a statement in the invitation e-mail that declining to participate in the study would not influence their course results.

For peer review only



For peer review only - http://bmjopen.bmj.com/site/about/guidelines.xhtml

The three measurement moments were scheduled in consultation with the participants, separate from the course. For each measurement moment, study participants were instructed to act in a simulation scenario as physician and to perform a primary assessment according to the ABCDE approach. One of the researchers participated as “non-obstructive” nurse and one researcher operated the simulator and computer.

**Patient and public involvement**

Participants of this study were not involved in the development of the research question, design or outcome measures. All participants of the study participated voluntarily.

**Study Protocol**

The first recording (T1) took place one to two weeks prior to the course. The second recording (T2) took place within one week after the course. The third recording (T3) took place between three to four months after the course.

The research team consisted of five physicians, who were also course instructors. They were all instructed in detail to only facilitate the simulation and not help the participant in any way.

The observers were two emergency physicians, who were also course instructors, but who were not part of the research team and therefore not involved in the recordings. The observers received specific instructions how to score each item on the assessment form. They independently rated the recorded primary assessments in random order and were blinded to the measurement moment.

For peer review only

**Measurements**

Each skill or competence on the assessment form had a lowest score of 0 and a highest score of 1, so the weight of each item was the same, independent of the two- (0 = not agree, 1 = agree, not applicable = missing value) or four-point scale (agree=1, partially agree=0.67, partially not agree=0.33, not agree=0, not applicable = missing value) or the number of examined items during the physical examination.

Because some skills or competences were marked as not applicable, we calculated mean scores in each category (A, B, C, D, E and remaining items) based on the skills and competences which actually were applicable. In each category the maximal score to obtain was 1. Therefore, the maximal total score to obtain on the primary assessment for each scenario was 6 and the minimal score was 0.

**Data analysis**

To perform the statistical analysis, IBM SPPSS version 23.0 was used. In all analyses, a p-value of < 0.05 was regarded as significant.

The inter-observer reliability between the scores given by the two observers for the three different time measurements was calculated using the Spearman's rank correlation test (for T1, T2 and T3 (resp. R 0.81, 0.61, and 0.80). This inter-observer reliability was generally high enough to average the mean scores of the two observers for each participant at T1, T2 and T3 for use in further analyses.

We used the Friedman test for three related samples to analyse whether the total primary assessment scores of the entire group of participants differed between the three measurement moments. The Friedman test compares the mean rank scores at T1, T2 and

For peer review only



For peer review only - http://bmjopen.bmj.com/site/about/guidelines.xhtml

T3. The mean rank score is calculated by ranking the score of each participant on T1, T2 and T3 and then calculating the mean rank of the entire group on T1, T2 and T3.

We used the Wilcoxon signed-rank test for two related samples to analyse whether the total primary assessment scores of the entire group of participants differed between two measurement moments and whether each skill or competence differed between two measurement moments. The Wilcoxon signed-rank test also uses the mean rank scores.

For peer review only

## RESULTS

### Characteristics of study subjects

Between August 2012 and December 2013, 27 courses were given to six participants each. From the total of 162 course participants 30 participants volunteered for this study. 21 were female, nine were male. Their mean age was 27 years (range 24-35, SD 2.77), their mean work experience was 11 months (range 0-48, SD14.4). Most participants did not have any experience with simulation training at all (18 out of 30), some participants had done some training in their own department, like ALS or Basic Life Support (BLS) (7 out of 30), from five participants we do not know whether they had any experience with simulation training.

The video recording of T3 of one participant was lost due to technical problems.

### Main results

The mean scores on the total primary assessments were 2.90, 5.06, and 4.67 at T1, T2 and T3 respectively (Table 1 and Figure 2). The maximal score possible was 6.

The mean rank scores of the entire group on the total primary assessments at T1, T2 en T3 were 1.14, 2.62 and 2.24, respectively (Table 1), and they were significantly different, ($p< 0.001$).

The mean rank score on the total primary assessment at T2 (directly after the course) was significantly higher than the mean rank score at T1 (before the course, table 1). The mean rank score on the total primary assessment at T3 (three months after the course) was significantly lower than the mean rank score at T2, but remained significantly higher than the mean rank score at T1 (Table 1).

Table 1: Scores on the total primary assessment of the whole group at T1, T2 and T3

| Time | N | Mean | SD | Median | Mean rank score | Wilcoxon signed-rank test |
|---|---|---|---|---|---|---|
| 1 | 30 | 2.90 | 0.88 | 2.79 | 1.14 | T1 < T2, $p<0.001$ |
| 2 | 30 | 5.06 | 0.48 | 5.22 | 2.62 | T2 > T3, $p<0.05$ |
| 3 | 29 | 4.67 | 0.75 | 4.70 | 2.24 | T3 > T1, $p<0.001$ |

For peer review only

The separate skills or competences were almost all scored significantly higher at T2 than at T1 (36 out of 41). With respect to the remaining skills, four could not be included in our analyses as they were scored too often as “does not apply”, which rendered the number of observations for those skills < N=10, which was too low to ascertain differences in a reliable way. For only one skill – “resuscitates adequately in the E” – we did not find a significant difference between T1 and T2.

Most of the separate skills did not show significant differences between T2 and T3 (30 out of 41). The skills (7 out of 41), “mentions abnormal findings in the C”, “recognizes life threatening conditions in the C”, “mentions conclusions in the C”, “examines D completely”, “communicates clearly”, “shows confidence” and “shows good leadership” were scored significantly lower at T3 than at T2, but significantly higher than at T1 (Table 2).

Table 2: Outcomes for separate skills and competences that decreased between T2 and T3

| Skill/Competence | Mean | | | SD | | | Wilcoxon signed-rank test | | |
|---|---|---|---|---|---|---|---|---|---|
| | T1 | T2 | T3 | T1 | T2 | T3 | N= | | |
| mentions abnormal findings in the C | 0.52 | 0.94 | 0.82 | 0.33 | 0.11 | 0.18 | 29 | T2 > T3<br>T1 < T3 | p<0.01<br>p<0.01 |
| recognizes live-threatening conditions in the C | 0.50 | 0.95 | 0.84 | 0.42 | 0.15 | 0.31 | 28 | T2 > T3<br>T1 < T3 | p<0.05<br>p<0.01 |
| mentions conclusions in the C | 0.27 | 0.78 | 0.64 | 0.29 | 0.23 | 0.35 | 28 | T2 > T3<br>T1 < T3 | p<0.05<br>p<0.01 |
| examines the D completely | 0.36 | 0.87 | 0.70 | 0.34 | 0.17 | 0.26 | 29 | T2 > T3<br>T1 < T3 | p<0.01<br>p<0.001 |
| communicates clearly | 0.52 | 0.87 | 0.78 | 0.19 | 0.14 | 0.15 | 29 | T2 > T3<br>T1 < T3 | p<0.05<br>p<0.001 |
| shows confidence | 0.43 | 0.86 | 0.75 | 0.23 | 0.14 | 0.15 | 29 | T2 > T3<br>T1 < T3 | p<0.05<br>p<0.001 |
| shows good leadership | 0.54 | 0.89 | 0.82 | 0.22 | 0.13 | 0.13 | 29 | T2 > T3<br>T1 < T3 | p<0.05<br>p<0.001 |

For peer review only

**DISCUSSION**

Our study is the first to show the effectiveness of a course using simulation to teach physicians the ABCDE approach for the assessment of medically ill patients. We found that the positive effect on performing a primary assessment according the ABCDE approach persisted even three months after completing the course.

Our findings corroborate outcomes of other studies showing that simulation training in health professions education was consistently associated with large effects on knowledge, skills and behaviour [[22, 23]]. Our findings are also in line with previous research showing that simulation training establishes, corrects, and confirms knowledge and skills of the ABCDE

approach and afterwards participants felt more competent in applying the ABCDE approach [3, 19-21].

In the follow-up we found decline in participant performance on some skills of the primary assessment. Strikingly, most skills that decrease over time are CRM-skills (Table 2). This is illustrated by a decrease in time of “recognition of life-threatening conditions in the C”, while the scores on the resuscitation skills did not decline. It is possible that this lower score reflects ‘not thinking out loud’ rather than failing to recognize a life-threatening condition. This decrease in CRM-skills suggests that focusing on CRM-skills by refresher courses or team training, after completing a simulation course, may be an important topic for physicians to maintain their skills. The positive effect of team training for these non-technical skills has already been shown [16-18].

Another skill that does not yield scores as high as most other skills after three months, is a complete examination of the Disability. A possible explanation for this finding is that the participants decide on the level of consciousness of the patient, determined by the EMV, whether it is necessary to examine certain components of the Disability. The performance of the EMV does not decrease over time. This finding is in line with previous research from our group on primary assessment completeness showing that during the primary assessment in the emergency department, residents and experienced staff have equal, but not maximum ABCDE completeness scores (83 instead of 100) [7]. Fernandez et al. also showed that professional lifeguards failed to fully perform the ABCDE sequence after simulation training and spend more time in the Circulation step, because they spent more time in steps considered most important [5].

These outcomes may reflect that a score of 100 on the ABCDE approach is not necessary to exclude potential life-threatening diseases or stabilize the patients.



For peer review only - http://bmjopen.bmj.com/site/about/guidelines.xhtml

**Limitations**

It is not possible to define the impact of the book and the lectures that are also part of the course, on the measured improvements in performance on the primary assessment. Outcomes from the regular course evaluation – not part of this study – indicated that the simulation training was the most powerful educational tool and accounted for most of the improvements. This feedback is in line with previous research showing that adding simulation training to a curriculum with lectures of medical students is associated with higher oral exam scores and higher overall course grades [[22]].

This study evaluated a course with instructors who are experts in the field of acute medicine, and experienced and certified course instructors. It is known that simulation-based education is most effective if guided by a safe and efficient debriefing and that debriefing can be challenging [[23, 24]]. We do not know if simulation training with debriefing by less experienced instructors may have less effect.

Another limitation of this study is that it was not possible to assess all specific skills in each simulation scenario, because they were often scored as “does not apply”. This limitation probably did not influence the results because items scored often as “does not apply” do not impact on discriminating in quality of performance.

The observers may have been the instructor during the course of some study participants and we cannot exclude that this may have influenced their ratings for some of them. This potential bias was minimized by offering the videos in random order and blinding the observers to measurement moment. Also, our study focused on the outcomes at group level and not individual outcomes.

For peer review only

Finally, the participants knew they were a study subject and when the recordings were scheduled. We do not know if they prepared for the study scenarios. Modifying behaviour in response to the awareness of being observed is known as the Hawthorne effect. This may have influenced the study scores at T1 and T3, whereas preparation for T3 might result in an overestimation of the actual course effectiveness at follow-up, preparation at T1 might result in an underestimation of the effectiveness of the course on both T2 and T3.

## Conclusion

A course with simulation training is an effective educational tool to teach physicians to perform a structured primary assessment using the ABCDE. This competence is largely remained after three months. CRM-skills tend to decrease over time, so we recommend organizing refresher courses, simulation team training or another kind of simulation training with a focus on CRM-skills.

## Figure legend

Figure 1 Assessment form used by the observers.

Figure 2 Boxplot comparing mean score on primary assessment at T1, T2 and T3.

## Data sharing statement

Unpublished statistical data will be available on request to the corresponding author.

## Acknowledgements

We want to thank Kinge van der Heide for her help with the recordings. We want to thank Mirjam Doff-Holman and Martine Oosterloo for the assessment of all recordings.

For peer review only

## REFERENCES

1 Soreide K. Three decades (1978-2008) of Advanced Trauma Life Support (ATLS) practice revised and evidence revisited. *Scand J Trauma Resusc Emerg Med* 2008;16:19,7241-16-19 doi:10.1186/1757-7241-16-19 [doi].

2 Jayaraman S, Sethi D, Chinnock P, et al. Advanced trauma life support training for hospital staff. *Cochrane Database Syst Rev* 2014;(8):CD004173. doi:CD004173 doi:10.1002/14651858.CD004173.pub4 [doi].

3 Abelsson A, Rystedt I, Suserud B, et al. Learning High-Energy Trauma Care Through Simulation. *CLIN SIMULATION NURS* 2018;17:1-6 doi:10.1016/j.ecns.2017.11.009.

4 Thim T, Krarup NHV, Grove EL, et al. Initial assessment and treatment with the Airway, Breathing, Circulation, Disability, Exposure (ABCDE) approach. *Int J Gen Med* 2012;5:117-21 doi:10.2147/IJGM.S28478.

5 Fernández-Méndez F, Otero-Agra M, Abelairas-Gómez C, et al. ABCDE approach to victims by lifeguards: How do they manage a critical patient? A cross sectional simulation study. *PLoS ONE* 2019;14 doi:10.1371/journal.pone.0212080.

6 Ilgen JS, Sherbino J, Cook DA. Technology-enhanced simulation in emergency medicine: a systematic review and meta-analysis. *Acad Emerg Med* 2013;20:117-27 doi:10.1111/acem.12076 [doi].

7 Olgers TJ, Dijkstra RS, Drost-de Klerck AM, et al. The ABCDE primary assessment in the emergency department in medically ill patients: an observational pilot study. *Neth J Med* 2017;75:106-11.

8 Franklin C, Mathew J. Developing strategies to prevent inhospital cardiac arrest: analyzing responses of physicians and nurses in the hours before the event. *Crit Care Med* 1994;22:244-7.

9 McQuillan P, Pilkington S, Allan A, et al. Confidential inquiry into quality of care before admission to intensive care. *BMJ* 1998;316:1853-8.

For peer review only

10 Kumar A, Roberts D, Wood KE, et al. Duration of hypotension before initiation of effective antimicrobial therapy is the critical determinant of survival in human septic shock. *Crit Care Med* 2006;34:1589-96 doi:10.1097/01.CCM.0000217961.75225.E9 [doi].

11 National Institute of Neurological Disorders and Stroke rt-PA Stroke Study Group. Tissue plasminogen activator for acute ischemic stroke. *N Engl J Med* 1995;333:1581-7 doi:10.1056/NEJM199512143332401 [doi].

12 Spoedeisende hulp: vanuit een stevige basis. Rapportage werkgroep levelindeling SEH. 2009 https://www.netwerkacutezorgnoordwest.nl/app/uploads/2017/01/200910-Rapport-SEH-Vanuit-een-stevig-basis.pdf.

13 Ryu WHA, Dharampal N, Mostafa AE, et al. Systematic Review of Patient-Specific Surgical Simulation: Toward Advancing Medical Education. *J Surg Educ* 2017 doi:S1931-7204(16)30269-0 [pii].

14 Price J, Naik V, Boodhwani M, et al. A randomized evaluation of simulation training on performance of vascular anastomosis on a high-fidelity in vivo model: the role of deliberate practice. *J Thorac Cardiovasc Surg* 2011;142:496-503 doi:10.1016/j.jtcvs.2011.05.015 [doi].

15 Kory PD, Eisen LA, Adachi M, et al. Initial airway management skills of senior residents: simulation training compared with traditional training. *Chest* 2007;132:1927-31 doi:S0012-3692(15)52468-9 [pii].

16 Buljac-Samardzic M, Dekker-van Doorn CM, van Wijngaarden JD, et al. Interventions to improve team effectiveness: a systematic review. *Health Policy* 2010;94:183-95 doi:10.1016/j.healthpol.2009.09.015 [doi].

17 Hesselink G, Berben S, Beune T, et al. Improving the governance of patient safety in emergency care: a systematic review of interventions. *BMJ Open* 2016;6:e009837,2015-009837 doi:10.1136/bmjopen-2015-009837 [doi].

18 Shapiro MJ, Morey JC, Small SD, et al. Simulation based teamwork training for emergency department staff: does it improve clinical team performance when added to an existing didactic teamwork curriculum?. *Qual Saf Health Care* 2004;13:417-21 doi:13/6/417 [pii].

19 Willett TG, Kirlew M, Cardinal P, et al. An evaluation of the Acute Critical Events Simulation (ACES) course for family medicine residents. *Can J Rural Med* 2011;16:89-95.

For peer review only



For peer review only - http://bmjopen.bmj.com/site/about/guidelines.xhtml

20 Carling J. Are graduate doctors adequately prepared to manage acutely unwell patients?. *Clin Teach* 2010;7:102-5 doi:10.1111/j.1743-498X.2010.00341.x [doi].

21 Pantelidis P, Staikoglou N, Paparoidamis G, et al. Medical students' satisfaction with the Applied Basic Clinical Seminar with Scenarios for Students, a novel simulation-based learning method in Greece. *J Educ Eval Health Prof* 2016;13:13 doi:10.3352/jeehp.2016.13.13 [doi].

22 Frallicciardi A, Fried J, Regan T, et al. A comparison of traditional lecture to simulation based cases for emergency medicine education among fourth year medical students. *Acad Emerg Med* 2012;19:S386 doi:10.1111/j.1553-2712.2012.01332.x.

23 Cook DA, Hatala R, Brydges R, et al. Technology-enhanced simulation for health professions education: a systematic review and meta-analysis. *JAMA* 2011;306:978-88 doi:10.1001/jama.2011.1234 [doi].

24 Grant VJ, Robinson T, Catena H, et al. Difficult debriefing situations: A toolbox for simulation educators. *Med Teach* 2018;40:703-12 doi:10.1080/0142159X.2018.1468558.

For peer review only

# Figure 1 Assessment form used by the observers.

| Primary assessment | | |
|---|---|---|
| A | - examines the airway<br>- mentions obstructed airway<br>- applies airway maneuvers<br>- applies oxygen | agree / disagree<br>agree / disagree/ d.n.a.<br>agree / partially agree / partially disagree / disagree / d.n.a.<br>agree / disagree/ d.n.a. |
| B | - examines B completely (color, trachea, resp. rate, excursions, breathing effort, percussion, auscultation, saturation)<br>- gives nurse the right orders<br>- mentions abnormal findings<br>- recognizes life-threatening conditions<br>- orders right additional diagnostic tests<br>- mentions conclusions/interpretation<br>- resuscitates adequately | 1 - 2 - 3 - 4 - 5 - 6 - 7 – 8<br>agree / partially agree / partially disagree / disagree / d.n.a.<br>agree / partially agree / partially disagree / disagree / d.n.a.<br>agree / disagree / d.n.a.<br>agree / disagree / d.n.a.<br>agree / partially agree / partially disagree / disagree<br>agree / partially agree / partially disagree / disagree / d.n.a. |
| C | - examines C completely (circulation of extremities, central pulse, heart rate, blood pressure, cap.refill, CVP, heart sounds)<br>- gives nurse the right orders<br>- mentions abnormal findings<br>- recognizes life-threatening conditions<br>- orders right additional diagnostic tests<br>- mentions conclusions/interpretation<br>- resuscitates adequately | 1 - 2 - 3 - 4 - 5 - 6 - 7<br>agree / partially agree / partially disagree / disagree<br>agree / partially agree / partially disagree / disagree<br>agree / disagree / d.n.a.<br>agree / disagree / d.n.a.<br>agree / partially agree / partially disagree / disagree<br>agree / partially agree / partially disagree / disagree / d.n.a. |
| D | - examines D completely (Glasgow Coma Scale, pupils, neck stiffness, glucose)<br>- applies EMC correctly<br>- mentions abnormal findings<br>- recognizes life-threatening conditions<br>- orders right additional diagnostic tests<br>- mentions conclusions/interpretation<br>- resuscitates adequately | 1 - 2 - 3 – 4<br>agree / partially agree / partially disagree / disagree<br>agree / partially agree / partially disagree / disagree/ d.n.a.<br>agree / disagree / d.n.a.<br>agree / disagree / d.n.a.<br>agree / partially agree / partially disagree / disagree<br>agree / partially agree / partially disagree / disagree / d.n.a. |
| E | - examines E completely (temperature, head to toe)<br>- gives nurse the right orders<br>- mentions abnormal findings<br>- orders right additional diagnostic tests<br>- mentions conclusions/interpretation<br>- resuscitates adequately | 1 – 2<br>agree / partially agree / partially disagree / disagree/ d.n.a.<br>agree / partially agree / partially disagree / disagree/ d.n.a.<br>agree / disagree / d.n.a.<br>agree / partially agree / partially disagree / disagree<br>agree / partially agree / partially disagree / disagree / d.n.a. |
| REMAINING | - asks for help adequately<br>- communicates clearly<br>- summarizes adequately<br>- draws the right conclusions<br>- clinical reasoning is adequate<br>- works structured<br>- stays calm<br>- shows confidence<br>- shows good leadership | agree / partially agree / partially disagree / disagree<br>agree / partially agree / partially disagree / disagree<br>agree / partially agree / partially disagree / disagree<br>agree / partially agree / partially disagree / disagree<br>agree / partially agree / partially disagree / disagree<br>agree / partially agree / partially disagree / disagree<br>agree / partially agree / partially disagree / disagree<br>agree / partially agree / partially disagree / disagree<br>agree / partially agree / partially disagree / disagree |

For peer review only

Figure 2 Boxplot comparing mean score on total primary assessment at T1, T2 and T3.

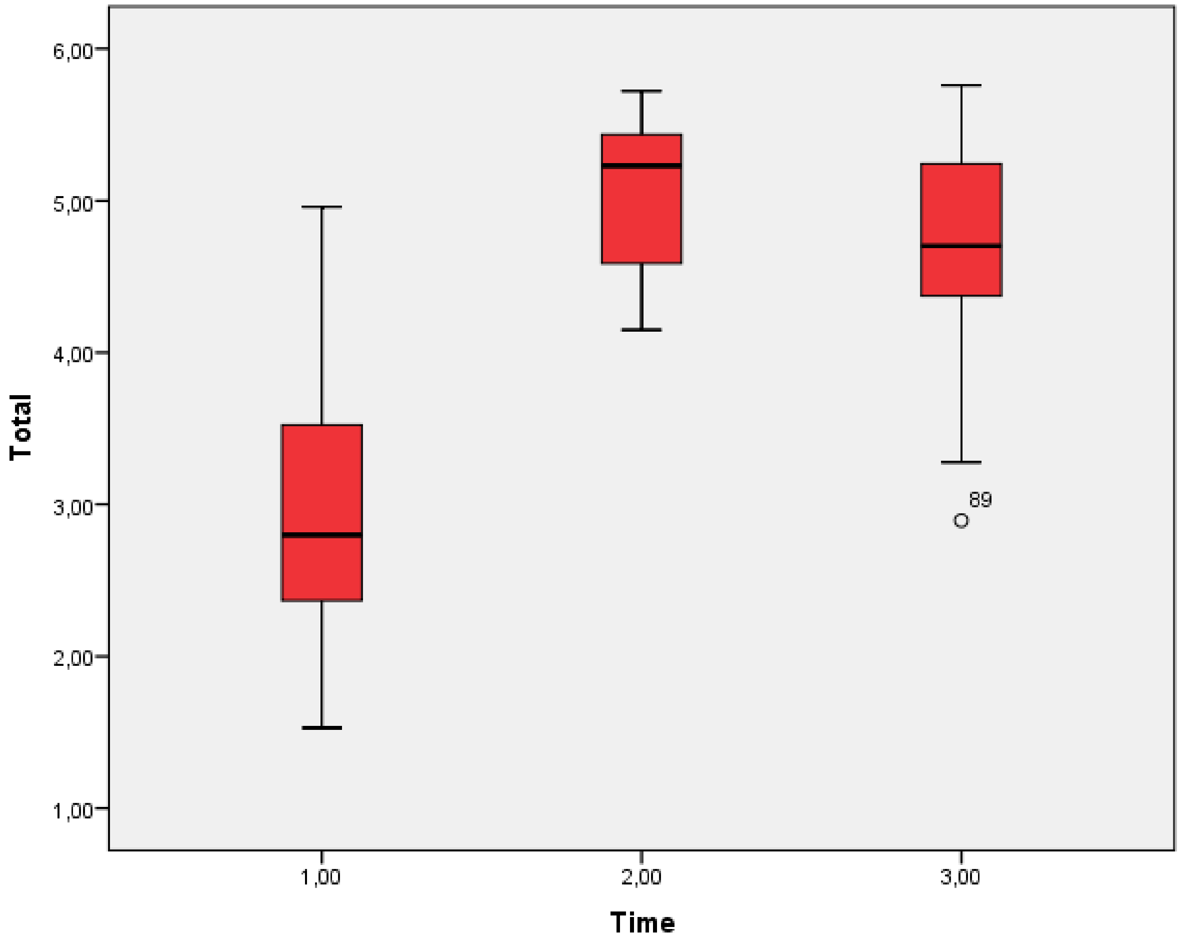


w only

STROBE Statement—checklist of items that should be included in reports of observational studies

| | Item No | Recommendation |
|---|---|---|
| **Title and abstract** | 1 | (*a*) Indicate the study's design with a commonly used term in the title or the abstract **Page 1** |
| | | (*b*) Provide in the abstract an informative and balanced summary of what was done and what was found **Page 4** |
| **Introduction** | | |
| Background/rationale | 2 | Explain the scientific background and rationale for the investigation being reported **Page 6 and 7** |
| Objectives | 3 | State specific objectives, including any prespecified hypotheses **Page 7** |
| **Methods** | | |
| Study design | 4 | Present key elements of study design early in the paper **Page 7-9** |
| Setting | 5 | Describe the setting, locations, and relevant dates, including periods of recruitment, exposure, follow-up, and data collection **Page 9-10** |
| Participants | 6 | (*a*) *Cohort study*—Give the eligibility criteria, and the sources and methods of selection of participants. Describe methods of follow-up **Page 7-11**<br>*Case-control study*—Give the eligibility criteria, and the sources and methods of case ascertainment and control selection. Give the rationale for the choice of cases and controls<br>*Cross-sectional study*—Give the eligibility criteria, and the sources and methods of selection of participants |
| | | (*b*) *Cohort study*—For matched studies, give matching criteria and number of exposed and unexposed<br>*Case-control study*—For matched studies, give matching criteria and the number of controls per case |
| Variables | 7 | Clearly define all outcomes, exposures, predictors, potential confounders, and effect modifiers. Give diagnostic criteria, if applicable **Page 7-12** |
| Data sources/ measurement | 8* | For each variable of interest, give sources of data and details of methods of assessment (measurement). Describe comparability of assessment methods if there is more than one group **Page 7-12** |
| Bias | 9 | Describe any efforts to address potential sources of bias **Page 7-12** |
| Study size | 10 | Explain how the study size was arrived at **(no effectsize known)** |
| Quantitative variables | 11 | Explain how quantitative variables were handled in the analyses. If applicable, describe which groupings were chosen and why |
| Statistical methods | 12 | (*a*) Describe all statistical methods, including those used to control for confounding **Page 12-13** |
| | | (*b*) Describe any methods used to examine subgroups and interactions |
| | | (*c*) Explain how missing data were addressed **Page 12** |
| | | (*d*) *Cohort study*—If applicable, explain how loss to follow-up was addressed<br>*Case-control study*—If applicable, explain how matching of cases and controls was addressed |

For peer review only

*Cross-sectional study*—If applicable, describe analytical methods taking account of sampling strategy

(*e*) Describe any sensitivity analyses **Page 12 (interobserver reliability)**

For peer review only



For peer review only - http://bmjopen.bmj.com/site/about/guidelines.xhtml

| | | |
|---|---|---|
| **Results** | | |
| Participants | 13* | (a) Report numbers of individuals at each stage of study—eg numbers potentially eligible, examined for eligibility, confirmed eligible, included in the study, completing follow-up, and analysed **Page 13** |
| | | (b) Give reasons for non-participation at each stage |
| | | (c) Consider use of a flow diagram |
| Descriptive data | 14* | (a) Give characteristics of study participants (eg demographic, clinical, social) and information on exposures and potential confounders **Page 13** |
| | | (b) Indicate number of participants with missing data for each variable of interest **Page 13 and supplemental file Outcome** |
| | | (c) *Cohort study*—Summarise follow-up time (eg, average and total amount) |
| Outcome data | 15* | *Cohort study*—Report numbers of outcome events or summary measures over time **Page 13-15** |
| | | *Case-control study*—Report numbers in each exposure category, or summary measures of exposure |
| | | *Cross-sectional study*—Report numbers of outcome events or summary measures |
| Main results | 16 | (*a*) Give unadjusted estimates and, if applicable, confounder-adjusted estimates and their precision (eg, 95% confidence interval). Make clear which confounders were adjusted for and why they were included **Page 13-15, Table 1 and 2** |
| | | (*b*) Report category boundaries when continuous variables were categorized |
| | | (*c*) If relevant, consider translating estimates of relative risk into absolute risk for a meaningful time period |
| Other analyses | 17 | Report other analyses done—eg analyses of subgroups and interactions, and sensitivity analyses **Page 12 (interobserver reliability)** |
| **Discussion** | | |
| Key results | 18 | Summarise key results with reference to study objectives **Page 15-16** |
| Limitations | 19 | Discuss limitations of the study, taking into account sources of potential bias or imprecision. Discuss both direction and magnitude of any potential bias **Page 17-18** |
| Interpretation | 20 | Give a cautious overall interpretation of results considering objectives, limitations, multiplicity of analyses, results from similar studies, and other relevant evidence **Page 15-18** |
| Generalisability | 21 | Discuss the generalisability (external validity) of the study results **Page 15-18** |
| **Other information** | | |
| Funding | 22 | Give the source of funding and the role of the funders for the present study and, if applicable, for the original study on which the present article is based **Page 3** |

*Give information separately for cases and controls in case-control studies and, if applicable, for exposed and unexposed groups in cohort and cross-sectional studies.

For peer review only

**Note:** An Explanation and Elaboration article discusses each checklist item and gives methodological background and published examples of transparent reporting. The STROBE checklist is best used in conjunction with this article (freely available on the Web sites of PLoS Medicine at http://www.plosmedicine.org/, Annals of Internal Medicine at http://www.annals.org/, and Epidemiology at http://www.epidem.com/). Information on the STROBE Initiative is available at www.strobe-statement.org.

For peer review only


For peer review only - http://bmjopen.bmj.com/site/about/guidelines.xhtml

BMJ Open

# Use of simulation training to teach the ABCDE primary assessment: an observational study in a Dutch University Hospital with a 3-months follow up.

| | |
|---|---|
| Journal: | *BMJ Open* |
| Manuscript ID | bmjopen-2019-032023.R2 |
| Article Type: | Original research |
| Date Submitted by the Author: | 28-Dec-2019 |
| Complete List of Authors: | Drost- de Klerck, Amanda; Universitair Medisch Centrum Groningen, emergency department<br>Olgers, Tycho; University Medical Center Groningen, Internal medicine, Emergency dept.<br>van de Meeberg, Evelien; Universitair Medisch Centrum Groningen, emergency department<br>Schonrock-Adema, Johanna; Universitair Medisch Centrum Groningen, emergency department<br>ter Maaten, Jan; University Medical Center Groningen, Emergency Department, Department of internal medicine |
| <b>Primary Subject Heading</b>: | Medical education and training |
| Secondary Subject Heading: | Emergency medicine |
| Keywords: | ACCIDENT & EMERGENCY MEDICINE, EDUCATION & TRAINING (see Medical Education & Training), GENERAL MEDICINE (see Internal Medicine), MEDICAL EDUCATION & TRAINING |
| | |

SCHOLARONE™
Manuscripts

I, the Submitting Author has the right to grant and does grant on behalf of all authors of the Work (as defined in the below author licence), an exclusive licence and/or a non-exclusive licence for contributions from authors who are: i) UK Crown employees; ii) where BMJ has agreed a CC-BY licence shall apply, and/or iii) in accordance with the terms applicable for US Federal Government officers or employees acting as part of their official duties; on a worldwide, perpetual, irrevocable, royalty-free basis to BMJ Publishing Group Ltd ("BMJ") its licensees and where the relevant Journal is co-owned by BMJ to the co-owners of the Journal, to publish the Work in this journal and any other BMJ products and to exploit all rights, as set out in our licence.

The Submitting Author accepts and understands that any supply made under these terms is made by BMJ to the Submitting Author unless you are acting as an employee on behalf of your employer or a postgraduate student of an affiliated institution which is paying any applicable article publishing charge ("APC") for Open Access articles. Where the Submitting Author wishes to make the Work available on an Open Access basis (and intends to pay the relevant APC), the terms of reuse of such Open Access shall be governed by a Creative Commons licence – details of these licences and which Creative Commons licence will apply to this Work are set out in our licence referred to above.

Other than as permitted in any relevant BMJ Author's Self Archiving Policies, I confirm this Work has not been accepted for publication elsewhere, is not being considered for publication elsewhere and does not duplicate material already published. I confirm all authors consent to publication of this Work and authorise the granting of this licence.

For peer review only

# Use of simulation training to teach the ABCDE primary assessment: an observational study in a Dutch University Hospital with a 3-months follow up.

*Amanda M Drost-de Klerck, Tycho J Olgers, Evelien K van de Meeberg, Johanna Schönrock-Adema, Jan C ter Maaten.*

*Emergency Department, University Medical Center Groningen, Hanzeplein 1, 9713 GZ Groningen, The Netherlands, Amanda M Drost-de Klerck, Consultant Emergency Medicine. Emergency Department, University Medical Center Groningen, Tycho J Olgers, Consultant Acute Internal Medicine. Emergency Department, University Medical Center Groningen, Evelien K van de Meeberg, Consultant Emergency Medicine. University of Groningen and University Medical Center Groningen, Institute for Medical Education, Johanna Schönrock-Adema, senior researcher. Emergency Department, University Medical Center Groningen, Jan C ter Maaten, Full Professor Acute Internal Medicine.*

**Word count:** 3414

**Address for correspondence/request for reprints:**

A.M. Drost-de Klerck

Phone: +31503616161 / Fax: +31503611725

Email: a.drost@umcg.nl

For peer review only

**Contributors**

AMDK and JCM have conceived the study and created the assessment form. AMDK, TJO, EKM and JCM collected the data. AMDK is guarantor and did the literature search, managed the data and performed data analysis and drafted the manuscript. JSA advised regarding to the data analysis. All authors contributed to the final manuscript, revision of the manuscript and final approval of the manuscript. The corresponding author attests that all listed authors meet authorship criteria and that no others meeting the criteria have been omitted.

For peer review only

**Copyright/license for publication**

The corresponding author has the right to grant on behalf of all authors and does grant on behalf of all authors, a worldwide licence to the Publishers and its licensees in perpetuity, in all forms, formats and media (whether known now or created in the future), to i) publish, reproduce, distribute, display and store the Contribution, ii) translate the Contribution into other languages, create adaptations, reprints, include within collections and create summaries, extracts and/or, abstracts of the Contribution, iii) create any other derivative work(s) based on the Contribution, iv) to exploit all subsidiary rights in the Contribution, v) the inclusion of electronic links from the Contribution to third party material where-ever it may be located; and, vi) licence any third party to do any or all of the above."

**Competing interests**

All authors have completed the ICMJE uniform disclosure form at www.icmje.org/coi_disclosure.pdf and declare: no support from any organisation for the

submitted work; no financial relationships with any organisations that might have an interest in the submitted work in the previous three years; no other relationships or activities that could appear to have influenced the submitted work.

**Ethics approval**

Ethical approval was waived by our medical ethics committee (METc UMC Groningen) as this research is educational research. We have received an independent review board declaration from our medical ethics committee, which declares that this study fulfills all the requirements for patient anonymity and is in agreement with regulations of our University Hospital.

**Transparency**

We declare that the manuscript is honest, accurate and transparent. No important aspects of the study have been omitted and discrepancies from the study as originally planned have been explained.

**Funding**

The study was only funded by the Emergency Department of the University Medical Center Groningen.

For peer review only

**ABSTRACT**

**Objectives** To investigate short- and long-term effectiveness of simulation training to acquire a structured Airway Breathing Circulation Disability Exposure (ABCDE) approach for medical emergencies; and to examine which skills were learned and maintained best.

**Design** an observational study with a 3-months follow-up.

**Setting** Skills centre of the University Medical Center Groningen.

**Participants** Thirty voluntary participants (21 females & 9 males; 27±2.77 years) of a simulation-based course.

**Intervention** A two-day ABCDE-teaching course for residents and non-residents. The course encompasses 24 simulations in which participants perform primary assessments of acute ill patients. Video recordings were taken of each participant performing a primary assessment, before (T1), directly after (T2) and three months after the intervention (T3).

**Main outcome measures** Physicians' performance in the ABCDE primary assessment at T1, T2 and T3. Two observers scored the primary assessments, blinded to measurement moment, using an assessment form to evaluate the performance with regard to skills essential for a structured ABCDE approach. The Friedman and Wilcoxon signed-rank test were used to compare physicians' performances on the subsequent measurement moments.

**Results** The mean rank scores on the total primary assessment at T1, T2 and T3 were 1.14, 2.62 and 2.24, respectively, and were significantly different, ($p < 0.001$).

The mean rank scores on the total primary assessment directly after the course (T2 vs. T1 $p<0.001$) and three to four months after the course (T3 vs. T1 $p<0.001$) were significantly better than before the course. Certain skills deteriorated during the follow-up. Strikingly, most skills that decrease over time are Crew Resources Management (CRM)-skills.


For peer review only

## Conclusion

A course using simulation training is an effective educational tool to teach physicians the ABCDE primary assessment. Certain CRM-skills decrease over time, so we recommend organizing refresher courses, simulation team training or another kind of simulation training with a focus on CRM-skills.

| Strengths and limitations of this study |
|---|
| **Strengths**<br>➢ This is an observational intervention study measuring the 3-months effect of a simulation course.<br>➢ This study used the same environment for intervention and study.<br>➢ The observers were blinded to measurement moment during this study on participants' performance on the primary assessment using the ABCDE approach.<br>**Limitations**<br>➢ The fact that the observers might have been also the instructor of some study participants, may have influenced their ratings for a few participants, but potential bias was minimized by offering the videos in random order and blinded to measurement moment, analysis showed a generally high inter-observer reliability and our study focused on the outcomes at group level and not individual outcomes.<br>➢ There might be bias in the fact that we do not know if the participants have prepared for the study scenario at T1 and T3 (the Hawthorne effect), whereas preparation for T3 might result in an overestimation of the actual course effectiveness at follow-up, preparation at T1 might result in an underestimation of the effectiveness of the course on both T2 and T3. |

For peer review only

## INTRODUCTION

### Background

In emergency medicine, assessing incoming patients in life-threatening conditions according to a structured approach is considered essential for successful resuscitation. The most widely used structured approach for early recognition and immediate treatment of life-threatening conditions is the “Airway-Breathing-Circulation-Disability-Exposure” (ABCDE) approach. The ABCDE approach is taught in the Advanced Trauma Life Support (ATLS) since 1978 and has been the standard approach in trauma since [1-3].

The use of the ABCDE primary assessment has also increased in other medical emergencies in recent years [4-7].

Using the ABCDE approach likely improves outcomes by helping health care professionals focus on the most life-threatening clinical problems and perform immediate resuscitation. Although solid empirical evidence for the usefulness of the ABCDE approach and its clinical benefits to patients is limited[1, 2], the importance of early treatment has been recognized in several emergencies such as trauma, stroke, sepsis and shock [1-5, 8-11].

### Importance

The Dutch inspection for healthcare requires that physicians treating non-trauma patients in the emergency department are ABCDE qualified [12]. Therefore, completing an ABCDE course is mandatory for physicians who work at the Emergency Department (ED). These courses usually contain lectures and simulation scenario training. Despite the wide use of simulation training for teaching the systematic ABCDE approach, little research has been done to analyse the effectiveness of simulation training in acquiring this structural approach.

Simulation training has been proven to be effective for learning technical skills and

For peer review only

maintaining skills that are not frequently used in daily practice, like airway management and surgical skills [13-15]. Simulation training can also improve communication, efficiency and safety during teamwork [16-18]. A few studies based on self-perceptions showed that simulation training improved participants' confidence levels; they felt more competent in applying the ABCDE approach and several other skills [3, 19-21].

To our knowledge, it has not been investigated before whether simulation training actually improves physicians' skills in performing the structured ABCDE approach.

Our study focused on the effectiveness of simulation training to acquire a structured ABCDE approach. Our main goal was to analyse the short- and long-term effectiveness of simulation training to acquire a structured ABCDE approach. We analysed the improvement in physicians' primary assessment scores as a result of the ABCDE simulation training.

We also investigated whether the skills acquired were maintained over a period of three months and which skills and competences were learned and maintained best.

## METHODS

### Study design

We conducted an observational intervention study. The intervention consisted of a two-day simulation-based ABCDE teaching course. The measurements through video recordings were obtained before (T1), directly after (T2) and 3-4 months after the intervention (T3).

Three simulation scenarios (A, B and C) with different medical emergencies were specifically designed for this study. Scenario A was a case with pneumosepsis and hypoglycaemia, a partially obstructed airway due to low consciousness and shock. Scenario B concerned a case with obstructive shock caused by pulmonary embolism and an opioid overdose with altered

For peer review only

consciousness. Scenario C was a case with meningococcal sepsis with a partially obstructed airway due to low consciousness, bronchospasm and shock. We have designed three different and realistic scenarios with comparable difficulty by creating a life-threatening condition which needs resuscitation in three of the five main items from the ABCDE.

To prevent bias caused by the type or difficulty of the simulation, we varied the order in which participants had to complete the three simulation scenarios in such a way that the different scenario sequences were equally divided over T1, T2 and T3 (participant 1: T1 scenario A, T2 scenario B, T3 scenario C; participant 2: T1 scenario B, T2 scenario C, T3 scenario A; participant 3: T1 scenario C, T2 scenario A, T3 scenario B, etcetera). We made a schedule in which the order of the scenarios was prescribed for each participant and participants were divided over the schedule in order of inclusion.

We developed an assessment form (Figure 1) to evaluate the participants' performance regarding skills and competences essential to assess medical emergencies. The assessment form was divided in six categories; five concerned the ABCDE structure and the sixth one contained remaining items. The remaining items focus on some Crew Resources Management (CRM) skills, like collaboration, communication, acknowledge own boundaries, and leadership. In each category, the skills or competences could be rated on a two- (agree, not agree, does not apply) or four-point scale (agree, partially agree, partially not agree, not agree or does not apply). We have added the option “does not apply”, because some skills were not required in some simulation scenarios. In the categories B, C, D and E also the scored. The following items could be scored; in the B: skin color, trachea position, respiratory rate, thorax excursions, breathing effort, lung percussion, lung auscultation and saturation; in the C: circulation of extremities, central pulse, heart rate, blood pressure,

For peer review only

capillary refill, central venous pressure, heart sounds; in the D: Glasgow Coma Scale (EMV), pupils, neck stiffness, glucose; in the E: temperature, head to toe examination (Figure 1).

**Intervention**

The ABCDE course is a two-day course for non-residents and first year residents which exists for ten years now. For most participants, it was a mandatory course that they need to pass before they were allowed to work in the ED. The course consisted mainly of simulation training and two theoretical lectures about airway management and Advanced Life Support (ALS). Previous to this course, the participants received a book with chapters describing the ABCDE approach and various acute medical emergencies.

The course focused on learning to recognize and treat life-threatening conditions, but also paid attention to some CRM-skills necessary for an efficient ABCDE approach.

This course was given in the skills centre in a room similar to a resuscitation room in the ED. The patient simulator used was a Laerdal Resusci Anne SkillTrainer with an upgrade Vitale Signs Sim Software Complete package. This simulator features heart and lung sounds, chest excursions, pulse and can show all vital signs on a separate monitor. With a separate computer, the sounds and vital signs can be changed during the scenario, to simulate several acute medical conditions.

Each course group consisted of six participants and two instructors. During the simulation rounds the group was split in half and two scenarios were run simultaneously in two separate rooms.

The course encompassed a total of 24 simulations with a patient simulator in which participants perform the primary assessment of acute ill patients. In each scenario, the role

For peer review only

of physician, “non-obstructive nurse” and observer were assigned to the three participants. One of the instructors operated the simulator and led the debriefing afterwards.

In eight scenarios, the participants fulfilled the role of physician; in the other scenarios, they carried out the role of “non-obstructing nurse” or observer.

The participants received a certificate if they passed the theoretical test and if they were, according to the instructors, capable of performing a structured primary assessment of an acute ill patient, with recognition and resuscitation of life-threatening conditions and adequate CRM-skills.

All course instructors have to follow a formalized educational program to become an instructor: First they have to pass the course as participant and have to work in the field of emergency medicine or acute care. Second they need to follow a two-day generic instructor course specifically developed for simulation training. Then they have to act as assistant-trainer for at least two courses and they need to write a report reflecting on their own role as instructor. Finally they are observed by an experienced instructor to become certified. As instructor, they have to teach the course least twice a year to stay competent and they need to follow the course-specific instructors day each year.

For peer review only

**Study setting and population**

This study was conducted in the same skills centre as were the course took place. During the video recordings, the simulator, materials and environment were also the same as during the course.

We approached all participants prior to this two-day course by e-mail and invited them to participate voluntarily in the study between August 2012 and December 2013. We



For peer review only - http://bmjopen.bmj.com/site/about/guidelines.xhtml

endeavoured to achieve a save response environment by a statement in the invitation e-mail that declining to participate in the study would not influence their course results.

The three measurement moments were scheduled in consultation with the participants, separate from the course. For each measurement moment, study participants were instructed to act in a simulation scenario as physician and to perform a primary assessment according to the ABCDE approach. One of the researchers participated as “non-obstructive” nurse and one researcher operated the simulator and computer.

**Patient and public involvement**

Participants of this study were not involved in the development of the research question, design or outcome measures. All participants of the study participated voluntarily they knew all information about the investigation and they could withdraw from the study at any moment, all provided verbal consent.

**Study Protocol**

The first recording (T1) took place one to two weeks prior to the course. The second recording (T2) took place within one week after the course. The third recording (T3) took place between three to four months after the course.

The research team consisted of five physicians, who were also course instructors. They were all instructed in detail to only facilitate the simulation and not help the participant in any way.

The observers were two emergency physicians, who were also course instructors, but who were not part of the research team and therefore not involved in the recordings. The observers received specific instructions how to score each item on the assessment form.



For peer review only - http://bmjopen.bmj.com/site/about/guidelines.xhtml

They independently rated the recorded primary assessments in random order and were blinded to the measurement moment.

**Measurements**

Each skill or competence on the assessment form had a lowest score of 0 and a highest score of 1, so the weight of each item was the same, independent of the two- (0 = not agree, 1 = agree, not applicable = missing value) or four-point scale (agree=1, partially agree=0.67, partially not agree=0.33, not agree=0, not applicable = missing value). This was the same for the number of examined items during the physical examination

Because some skills or competences were marked as not applicable, we calculated mean scores in each category (A, B, C, D, E and remaining items) based on the skills and competences which actually were applicable. In each category the maximal score to obtain was 1. Therefore, the maximal total score to obtain on the primary assessment for each scenario was 6 and the minimal score was 0.

**Data analysis**

To perform the statistical analysis, IBM SPPSS version 23.0 was used. In all analyses, a p-value of < 0.05 was regarded as significant.

The inter-observer reliability between the scores given by the two observers for the three different time measurements was calculated using the Spearman's rank correlation test (for T1, T2 and T3 (resp. R 0.81, 0.61, and 0.80). This inter-observer reliability was generally high enough to average the mean scores of the two observers for each participant at T1, T2 and T3 for use in further analyses.

For peer review only

We used the Friedman test for three related samples to analyse whether the total primary assessment scores of the entire group of participants differed between the three measurement moments. The Friedman test compares the mean rank scores at T1, T2 and T3. The mean rank score is calculated by ranking the score of each participant on T1, T2 and T3 and then calculating the mean rank of the entire group on T1, T2 and T3.

We used the Wilcoxon signed-rank test for two related samples to analyse whether the total primary assessment scores of the entire group of participants differed between two measurement moments and whether each skill or competence differed between two measurement moments. The Wilcoxon signed-rank test also uses the mean rank scores. Finally, we applied the Holm correction to reduce the possibility of getting a statistically significant result (Type I error) when performing multiple tests.

## RESULTS

### Characteristics of study subjects

Between August 2012 and December 2013, 27 courses were given to six participants each. From the total of 162 course participants 30 participants volunteered for this study. 21 were female, nine were male. Their mean age was 27 years (range 24-35, SD 2.77), their mean work experience was 11 months (range 0-48, SD14.4). Most participants did not have any experience with simulation training at all (18 out of 30), some participants had done some training in their own department, like ALS or Basic Life Support (BLS) (7 out of 30), from five participants we do not know whether they had any experience with simulation training. The video recording of T3 of one participant was lost due to technical problems.

### Main results



For peer review only - http://bmjopen.bmj.com/site/about/guidelines.xhtml

The mean rank scores of the entire group on the total primary assessments at T1, T2 en T3 were 1.14, 2.62 and 2.24, respectively (Table 1), and they were significantly different, (p< 0.001).

The mean rank score on the total primary assessment at T2 (directly after the course) was significantly higher than the mean rank score at T1 (before the course, table 1). The mean rank score on the total primary assessment at T3 (three months after the course) was significantly lower than the mean rank score at T2, but remained significantly higher than the mean rank score at T1 (Table 1).

Table 1: Scores on the total primary assessment of the whole group at T1, T2 and T3

| Time | N | Median | Mean rank score | Wilcoxon signed-rank test |
|---|---|---|---|---|
| 1 | 30 | 2.79 | 1.14 | T1 < T2, p<0.001 |
| 2 | 30 | 5.22 | 2.62 | T2 > T3, p<0.05 |
| 3 | 29 | 4.70 | 2.24 | T3 > T1, p<0.001 |

The separate skills or competences were almost all scored significantly higher at T2 than at T1 (34 out of 41). With respect to the remaining skills, four could not be included in our analyses as they were scored too often as "does not apply", which rendered the number of observations for those skills < N=10, which was too low to ascertain differences in a reliable way. For only three skills – "examines the airway", "orders additional diagnostics in the B" and "resuscitates adequately in the E"– we did not find a significant difference between T1 and T2.

Most of the separate skills did not show significant differences between T2 and T3 (30 out of 41). Some skills (7 out of 41) were scored significantly lower at T3 than at T2, but significantly higher than at T1 (Table 2).



For peer review only - http://bmjopen.bmj.com/site/about/guidelines.xhtml

Table 2: Outcomes for separate skills and competences that decreased between T2 and T3

| Skill/Competence | Wilcoxon signed-rank test | | |
|---|---|---|---|
| | N= | | |
| mentions abnormal findings in the C | 29 | T2 > T3<br>T1 < T3 | p<0.01<br>p<0.01 |
| recognizes live-threatening conditions in the C | 28 | T2 > T3<br>T1 < T3 | p<0.05<br>p<0.01 |
| mentions conclusions in the C | 28 | T2 > T3<br>T1 < T3 | p<0.05<br>p<0.01 |
| examines the D completely | 29 | T2 > T3<br>T1 < T3 | p<0.01<br>p<0.001 |
| communicates clearly | 29 | T2 > T3<br>T1 < T3 | p<0.05<br>p<0.001 |
| shows confidence | 29 | T2 > T3<br>T1 < T3 | p<0.05<br>p<0.001 |
| shows good leadership | 29 | T2 > T3<br>T1 < T3 | p<0.05<br>p<0.001 |

For peer review only

**DISCUSSION**

Our study is the first to show the effectiveness of a course using simulation to teach physicians the ABCDE approach for the assessment of medically ill patients. We found that the positive effect on performing a primary assessment according the ABCDE approach persisted even 3-4 months after completing the course.

Our findings corroborate outcomes of other studies showing that simulation training in health professions education was consistently associated with large effects on knowledge, skills and behaviour [[22, 23]]. Our findings are also in line with previous research showing that



For peer review only - http://bmjopen.bmj.com/site/about/guidelines.xhtml

simulation training establishes, corrects, and confirms knowledge and skills of the ABCDE approach and afterwards participants felt more competent in applying the ABCDE approach [3, 19-21].

In the follow-up we found decline in participant performance on some skills of the primary assessment. Strikingly, most skills that decrease over time are CRM-skills (Table 2). This is illustrated by a decrease in time of “recognition of life-threatening conditions in the C”, while the scores on the resuscitation skills did not decline. It is possible that this lower score reflects ‘not thinking out loud’ rather than failing to recognize a life-threatening condition. This decrease in CRM-skills suggests that focusing on CRM-skills by refresher courses or team training, after completing a simulation course, may be an important topic for physicians to maintain their skills. The positive effect of team training for these non-technical skills has already been shown [16-18].

Another skill that does not yield scores as high as most other skills after three months, is a complete examination of the Disability. A possible explanation for this finding is that the participants decide on the level of consciousness of the patient, determined by the EMV, whether it is necessary to examine certain components of the Disability. The performance of the EMV does not decrease over time. This finding is in line with previous research from our group on primary assessment completeness showing that during the primary assessment in the emergency department, residents and experienced staff have equal, but not maximum ABCDE completeness scores (83 instead of 100) [7]. Fernandez et al. also showed that professional lifeguards failed to fully perform the ABCDE sequence and spend more time in the Circulation step, because they spent more time in steps considered most important [5]. These outcomes may reflect that a score of 100 on the ABCDE approach is not necessary to exclude potential life-threatening diseases or stabilize the patients.



For peer review only - http://bmjopen.bmj.com/site/about/guidelines.xhtml

**Limitations**

It is not possible to define the impact of the book and the lectures that are also part of the course, on the measured improvements in performance on the primary assessment. Outcomes from the regular course evaluation – not part of this study – indicated that the simulation training was the most powerful educational tool and accounted for most of the improvements. This feedback is in line with previous research showing that adding simulation training to a curriculum with lectures of medical students is associated with higher oral exam scores and higher overall course grades [[22]].

This study evaluated a course with instructors who are experts in the field of acute medicine, and experienced and certified course instructors. It is known that simulation-based education is most effective if guided by a safe and efficient debriefing and that debriefing can be challenging [[23, 24]]. We do not know if simulation training with debriefing by less experienced instructors may have less effect.

During the study we have deliberately chosen for a researcher participating as “non-obstructive nurse” in the measurement to minimize potential bias caused by help form the “non-obstructive nurse”. The researcher knew the research questions and was instructed in detail to only follow instructions from the participant and not help in any way.

We did not schedule the researchers and operators with an equal distribution over the measurement moments, but all five researchers rotated between roles of the nurse and operator on own initiative. We therefor think that the bias of the non-obstructive nurse influencing the participant is negligible.

Another limitation of this study is that it was not possible to assess all specific skills in each simulation scenario, because they were often scored as “does not apply”. The amount of not

For peer review only

applicable rated items was between 0-3 in ten items, between 3-10 in five items, between 10-20 and in four items the not applicable rated items was > 20. This limitation probably did not influence the results because items often scored as “does not apply” do not impact discriminating in quality of performance.

The sample size was chosen without power analysis, because we didn’t know the expected effect. This relative small sample size of 30 participants already showed large significant differences. In our statistical analysis we accounted for a small sample size by using the Wilcoxon signed-rank test.

The observers may have been the instructor during the course of some study participants and we cannot exclude that this may have influenced their ratings for some of them. This potential bias was minimized by offering the videos in random order and blinding the observers to the measurement moment. Also, our study focused on the outcomes at group level and not individual outcomes and the inter-observer reliability was generally high.

The measurement moment of T3 varied between 3-4 months after T2. We do not know if this range of one month between T2 and T3 have caused a variation in performance at T3.

Some participants had experience with simulation training. These participants might have had a higher score on T1 what might have caused an underestimation of the difference between T1 and T2.

Finally, the participants knew they were a study subject and when the recordings were scheduled. We do not know if they prepared for the study scenarios. Modifying behaviour in response to the awareness of being observed is known as the Hawthorne effect. This may have influenced the study scores at T1 and T3, whereas preparation for T3 might result in an overestimation of the actual course effectiveness at follow-up, preparation at T1 might result in an underestimation of the effectiveness of the course on both T2 and T3.

For peer review only

## Conclusion

A course with simulation training is an effective educational tool to teach physicians to perform a structured primary assessment using the ABCDE. This competence is largely remained after three months. CRM-skills tend to decrease over time, so we recommend organizing refresher courses, simulation team training or another kind of simulation training with a focus on CRM-skills.

For peer review only

## Figure legend

Figure 1 Assessment form used by the observers.

Figure 2 Boxplot comparing mean score on primary assessment at T1, T2 and T3.

## Data sharing statement

Unpublished statistical data will be available on request to the corresponding author.

## Acknowledgements

We want to thank Kinge van der Heide for her help with the recordings. We want to thank Mirjam Doff-Holman and Martine Oosterloo for the assessment of all recordings.

## REFERENCES

1 Soreide K. Three decades (1978-2008) of Advanced Trauma Life Support (ATLS) practice revised and evidence revisited. *Scand J Trauma Resusc Emerg Med* 2008;16:19,7241-16-19 doi:10.1186/1757-7241-16-19 [doi].

2 Jayaraman S, Sethi D, Chinnock P, et al. Advanced trauma life support training for hospital staff. *Cochrane Database Syst Rev* 2014;(8):CD004173. doi:CD004173 doi:10.1002/14651858.CD004173.pub4 [doi].

3 Abelsson A, Rystedt I, Suserud B, et al. Learning High-Energy Trauma Care Through Simulation. *CLIN SIMULATION NURS* 2018;17:1-6 doi:10.1016/j.ecns.2017.11.009.

4 Thim T, Krarup NHV, Grove EL, et al. Initial assessment and treatment with the Airway, Breathing, Circulation, Disability, Exposure (ABCDE) approach. *Int J Gen Med* 2012;5:117-21 doi:10.2147/IJGM.S28478.

5 Fernández-Méndez F, Otero-Agra M, Abelairas-Gómez C, et al. ABCDE approach to victims by lifeguards: How do they manage a critical patient? A cross sectional simulation study. *PLoS ONE* 2019;14 doi:10.1371/journal.pone.0212080.

6 Ilgen JS, Sherbino J, Cook DA. Technology-enhanced simulation in emergency medicine: a systematic review and meta-analysis. *Acad Emerg Med* 2013;20:117-27 doi:10.1111/acem.12076 [doi].

7 Olgers TJ, Dijkstra RS, Drost-de Klerck AM, et al. The ABCDE primary assessment in the emergency department in medically ill patients: an observational pilot study. *Neth J Med* 2017;75:106-11.

8 Franklin C, Mathew J. Developing strategies to prevent inhospital cardiac arrest: analyzing responses of physicians and nurses in the hours before the event. *Crit Care Med* 1994;22:244-7.

9 McQuillan P, Pilkington S, Allan A, et al. Confidential inquiry into quality of care before admission to intensive care. *BMJ* 1998;316:1853-8.

10 Kumar A, Roberts D, Wood KE, et al. Duration of hypotension before initiation of effective antimicrobial therapy is the critical determinant of survival in human septic shock. *Crit Care Med* 2006;34:1589-96 doi:10.1097/01.CCM.0000217961.75225.E9 [doi].

11 National Institute of Neurological Disorders and Stroke rt-PA Stroke Study Group. Tissue plasminogen activator for acute ischemic stroke. *N Engl J Med* 1995;333:1581-7 doi:10.1056/NEJM199512143332401 [doi].

12 Spoedeisende hulp: vanuit een stevige basis. Rapportage werkgroep levelindeling SEH. 2009 https://www.netwerkacutezorgnoordwest.nl/app/uploads/2017/01/200910-Rapport-SEH-Vanuit-een-stevig-basis.pdf.

For peer review only

13 Ryu WHA, Dharampal N, Mostafa AE, et al. Systematic Review of Patient-Specific Surgical Simulation: Toward Advancing Medical Education. *J Surg Educ* 2017 doi:S1931-7204(16)30269-0 [pii].

14 Price J, Naik V, Boodhwani M, et al. A randomized evaluation of simulation training on performance of vascular anastomosis on a high-fidelity in vivo model: the role of deliberate practice. *J Thorac Cardiovasc Surg* 2011;142:496-503 doi:10.1016/j.jtcvs.2011.05.015 [doi].

15 Kory PD, Eisen LA, Adachi M, et al. Initial airway management skills of senior residents: simulation training compared with traditional training. *Chest* 2007;132:1927-31 doi:S0012-3692(15)52468-9 [pii].

16 Buljac-Samardzic M, Dekker-van Doorn CM, van Wijngaarden JD, et al. Interventions to improve team effectiveness: a systematic review. *Health Policy* 2010;94:183-95 doi:10.1016/j.healthpol.2009.09.015 [doi].

17 Hesselink G, Berben S, Beune T, et al. Improving the governance of patient safety in emergency care: a systematic review of interventions. *BMJ Open* 2016;6:e009837,2015-009837 doi:10.1136/bmjopen-2015-009837 [doi].

18 Shapiro MJ, Morey JC, Small SD, et al. Simulation based teamwork training for emergency department staff: does it improve clinical team performance when added to an existing didactic teamwork curriculum?. *Qual Saf Health Care* 2004;13:417-21 doi:13/6/417 [pii].

19 Willett TG, Kirlew M, Cardinal P, et al. An evaluation of the Acute Critical Events Simulation (ACES) course for family medicine residents. *Can J Rural Med* 2011;16:89-95.

20 Carling J. Are graduate doctors adequately prepared to manage acutely unwell patients?. *Clin Teach* 2010;7:102-5 doi:10.1111/j.1743-498X.2010.00341.x [doi].

21 Pantelidis P, Staikoglou N, Paparoidamis G, et al. Medical students' satisfaction with the Applied Basic Clinical Seminar with Scenarios for Students, a novel simulation-based learning method in Greece. *J Educ Eval Health Prof* 2016;13:13 doi:10.3352/jeehp.2016.13.13 [doi].

22 Frallicciardi A, Fried J, Regan T, et al. A comparison of traditional lecture to simulation based cases for emergency medicine education among fourth year medical students. *Acad Emerg Med* 2012;19:S386 doi:10.1111/j.1553-2712.2012.01332.x.

For peer review only

23 Cook DA, Hatala R, Brydges R, et al. Technology-enhanced simulation for health professions education: a systematic review and meta-analysis. *JAMA* 2011;306:978-88 doi:10.1001/jama.2011.1234 [doi].

24 Grant VJ, Robinson T, Catena H, et al. Difficult debriefing situations: A toolbox for simulation educators. *Med Teach* 2018;40:703-12 doi:10.1080/0142159X.2018.1468558.

For peer review only

# Figure 1 Assessment form used by the observers.

| Primary assessment | | |
|---|---|---|
| A | - examines the airway<br>- mentions obstructed airway<br>- applies airway maneuvers<br>- applies oxygen | agree / disagree<br>agree / disagree/ d.n.a.<br>agree / partially agree / partially disagree / disagree / d.n.a.<br>agree / disagree/ d.n.a. |
| B | - examines B completely (color, trachea, resp. rate, excursions, breathing effort, percussion, auscultation, saturation)<br>- gives nurse the right orders<br>- mentions abnormal findings<br>- recognizes life-threatening conditions<br>- orders right additional diagnostic tests<br>- mentions conclusions/interpretation<br>- resuscitates adequately | 1 - 2 - 3 - 4 - 5 - 6 - 7 – 8<br>agree / partially agree / partially disagree / disagree / d.n.a.<br>agree / partially agree / partially disagree / disagree / d.n.a.<br>agree / disagree / d.n.a.<br>agree / disagree / d.n.a.<br>agree / partially agree / partially disagree / disagree<br>agree / partially agree / partially disagree / disagree / d.n.a. |
| C | - examines C completely (circulation of extremities, central pulse, heart rate, blood pressure, cap.refill, CVP, heart sounds)<br>- gives nurse the right orders<br>- mentions abnormal findings<br>- recognizes life-threatening conditions<br>- orders right additional diagnostic tests<br>- mentions conclusions/interpretation<br>- resuscitates adequately | 1 - 2 - 3 - 4 - 5 - 6 - 7<br>agree / partially agree / partially disagree / disagree / d.n.a.<br>agree / partially agree / partially disagree / disagree / d.n.a.<br>agree / disagree / d.n.a.<br>agree / disagree / d.n.a.<br>agree / partially agree / partially disagree / disagree<br>agree / partially agree / partially disagree / disagree / d.n.a. |
| D | - examines D completely (Glasgow Coma Scale, pupils, neck stiffness, glucose)<br>- applies EMC correctly<br>- mentions abnormal findings<br>- recognizes life-threatening conditions<br>- orders right additional diagnostic tests<br>- mentions conclusions/interpretation<br>- resuscitates adequately | 1 - 2 - 3 – 4<br>agree / partially agree / partially disagree / disagree<br>agree / partially agree / partially disagree / disagree/ d.n.a.<br>agree / disagree / d.n.a.<br>agree / disagree / d.n.a.<br>agree / partially agree / partially disagree / disagree<br>agree / partially agree / partially disagree / disagree / d.n.a. |
| E | - examines E completely (temperature, head to toe)<br>- gives nurse the right orders<br>- mentions abnormal findings<br>- orders right additional diagnostic tests<br>- mentions conclusions/interpretation<br>- resuscitates adequately | 1 – 2<br>agree / partially agree / partially disagree / disagree/ d.n.a.<br>agree / partially agree / partially disagree / disagree/ d.n.a.<br>agree / disagree / d.n.a.<br>agree / partially agree / partially disagree / disagree<br>agree / partially agree / partially disagree / disagree / d.n.a. |
| R<br>E<br>M<br>A<br>I<br>N<br>I<br>N<br>G | - asks for help adequately<br>- communicates clearly<br>- summarizes adequately<br>- draws the right conclusions<br>- clinical reasoning is adequate<br>- works structured<br>- stays calm<br>- shows confidence<br>- shows good leadership | agree / partially agree / partially disagree / disagree<br>agree / partially agree / partially disagree / disagree<br>agree / partially agree / partially disagree / disagree<br>agree / partially agree / partially disagree / disagree<br>agree / partially agree / partially disagree / disagree<br>agree / partially agree / partially disagree / disagree<br>agree / partially agree / partially disagree / disagree<br>agree / partially agree / partially disagree / disagree<br>agree / partially agree / partially disagree / disagree |

For peer review only

Figure 2 Boxplot comparing mean score on total primary assessment at T1, T2 and T3.

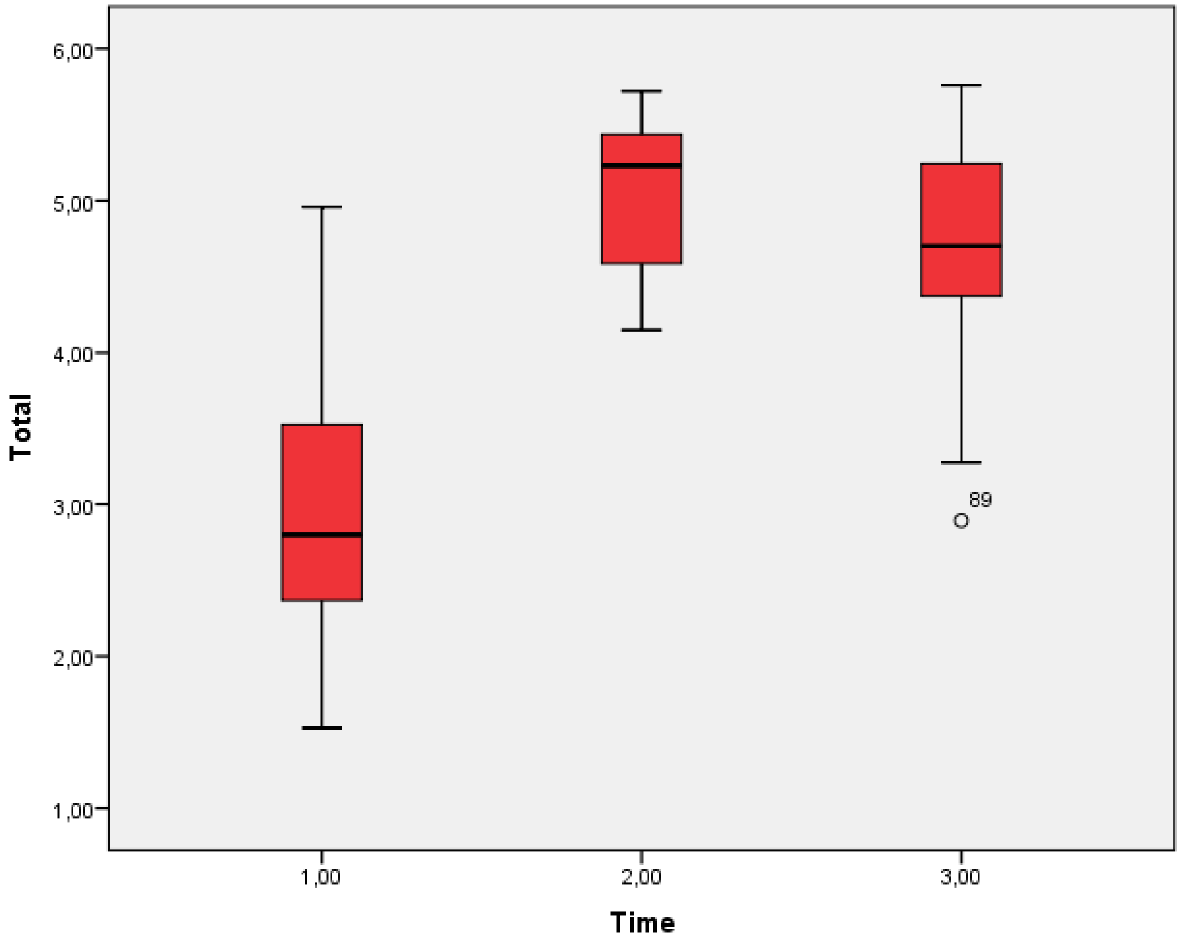


w only

STROBE Statement—checklist of items that should be included in reports of observational studies

| | Item No | Recommendation |
|---|---|---|
| **Title and abstract** | 1 | (*a*) Indicate the study's design with a commonly used term in the title or the abstract **Page 1** |
| | | (*b*) Provide in the abstract an informative and balanced summary of what was done and what was found **Page 4** |
| **Introduction** | | |
| Background/rationale | 2 | Explain the scientific background and rationale for the investigation being reported **Page 6 and 7** |
| Objectives | 3 | State specific objectives, including any prespecified hypotheses **Page 7** |
| **Methods** | | |
| Study design | 4 | Present key elements of study design early in the paper **Page 7-9** |
| Setting | 5 | Describe the setting, locations, and relevant dates, including periods of recruitment, exposure, follow-up, and data collection **Page 9-10** |
| Participants | 6 | (*a*) *Cohort study*—Give the eligibility criteria, and the sources and methods of selection of participants. Describe methods of follow-up **Page 7-11**<br>*Case-control study*—Give the eligibility criteria, and the sources and methods of case ascertainment and control selection. Give the rationale for the choice of cases and controls<br>*Cross-sectional study*—Give the eligibility criteria, and the sources and methods of selection of participants |
| | | (*b*) *Cohort study*—For matched studies, give matching criteria and number of exposed and unexposed<br>*Case-control study*—For matched studies, give matching criteria and the number of controls per case |
| Variables | 7 | Clearly define all outcomes, exposures, predictors, potential confounders, and effect modifiers. Give diagnostic criteria, if applicable **Page 7-12** |
| Data sources/ measurement | 8* | For each variable of interest, give sources of data and details of methods of assessment (measurement). Describe comparability of assessment methods if there is more than one group **Page 7-12** |
| Bias | 9 | Describe any efforts to address potential sources of bias **Page 7-12** |
| Study size | 10 | Explain how the study size was arrived at **(no effectsize known)** |
| Quantitative variables | 11 | Explain how quantitative variables were handled in the analyses. If applicable, describe which groupings were chosen and why |
| Statistical methods | 12 | (*a*) Describe all statistical methods, including those used to control for confounding **Page 12-13** |
| | | (*b*) Describe any methods used to examine subgroups and interactions |
| | | (*c*) Explain how missing data were addressed **Page 12** |
| | | (*d*) *Cohort study*—If applicable, explain how loss to follow-up was addressed<br>*Case-control study*—If applicable, explain how matching of cases and controls was addressed |



For peer review only - http://bmjopen.bmj.com/site/about/guidelines.xhtml

*Cross-sectional study*—If applicable, describe analytical methods taking account of sampling strategy

(*e*) Describe any sensitivity analyses **Page 12 (interobserver reliability)**

For peer review only


For peer review only - http://bmjopen.bmj.com/site/about/guidelines.xhtml

| | | |
|---|---|---|
| **Results** | | |
| Participants | 13* | (a) Report numbers of individuals at each stage of study—eg numbers potentially eligible, examined for eligibility, confirmed eligible, included in the study, completing follow-up, and analysed **Page 13** |
| | | (b) Give reasons for non-participation at each stage |
| | | (c) Consider use of a flow diagram |
| Descriptive data | 14* | (a) Give characteristics of study participants (eg demographic, clinical, social) and information on exposures and potential confounders **Page 13** |
| | | (b) Indicate number of participants with missing data for each variable of interest **Page 13 and supplemental file Outcome** |
| | | (c) *Cohort study*—Summarise follow-up time (eg, average and total amount) |
| Outcome data | 15* | *Cohort study*—Report numbers of outcome events or summary measures over time **Page 13-15** |
| | | *Case-control study*—Report numbers in each exposure category, or summary measures of exposure |
| | | *Cross-sectional study*—Report numbers of outcome events or summary measures |
| Main results | 16 | (*a*) Give unadjusted estimates and, if applicable, confounder-adjusted estimates and their precision (eg, 95% confidence interval). Make clear which confounders were adjusted for and why they were included **Page 13-15, Table 1 and 2** |
| | | (*b*) Report category boundaries when continuous variables were categorized |
| | | (*c*) If relevant, consider translating estimates of relative risk into absolute risk for a meaningful time period |
| Other analyses | 17 | Report other analyses done—eg analyses of subgroups and interactions, and sensitivity analyses **Page 12 (interobserver reliability)** |
| **Discussion** | | |
| Key results | 18 | Summarise key results with reference to study objectives **Page 15-16** |
| Limitations | 19 | Discuss limitations of the study, taking into account sources of potential bias or imprecision. Discuss both direction and magnitude of any potential bias **Page 17-18** |
| Interpretation | 20 | Give a cautious overall interpretation of results considering objectives, limitations, multiplicity of analyses, results from similar studies, and other relevant evidence **Page 15-18** |
| Generalisability | 21 | Discuss the generalisability (external validity) of the study results **Page 15-18** |
| **Other information** | | |
| Funding | 22 | Give the source of funding and the role of the funders for the present study and, if applicable, for the original study on which the present article is based **Page 3** |

*Give information separately for cases and controls in case-control studies and, if applicable, for exposed and unexposed groups in cohort and cross-sectional studies.



For peer review only - http://bmjopen.bmj.com/site/about/guidelines.xhtml

For peer review only

**Note:** An Explanation and Elaboration article discusses each checklist item and gives methodological background and published examples of transparent reporting. The STROBE checklist is best used in conjunction with this article (freely available on the Web sites of PLoS Medicine at http://www.plosmedicine.org/, Annals of Internal Medicine at http://www.annals.org/, and Epidemiology at http://www.epidem.com/). Information on the STROBE Initiative is available at www.strobe-statement.org.

For peer review only



For peer review only - http://bmjopen.bmj.com/site/about/guidelines.xhtml

BMJ Open

# Use of simulation training to teach the ABCDE primary assessment: an observational study in a Dutch University Hospital with a 3-4 months follow up.

| | |
|---|---|
| Journal: | *BMJ Open* |
| Manuscript ID | bmjopen-2019-032023.R3 |
| Article Type: | Original research |
| Date Submitted by the Author: | 04-Feb-2020 |
| Complete List of Authors: | Drost- de Klerck, Amanda; Universitair Medisch Centrum Groningen, emergency department<br>Olgers, Tycho; University Medical Center Groningen, Internal medicine, Emergency dept.<br>van de Meeberg, Evelien; Universitair Medisch Centrum Groningen, emergency department<br>Schonrock-Adema, Johanna; Universitair Medisch Centrum Groningen, emergency department<br>ter Maaten, Jan; University Medical Center Groningen, Emergency Department, Department of internal medicine |
| <b>Primary Subject Heading</b>: | Medical education and training |
| Secondary Subject Heading: | Emergency medicine |
| Keywords: | ACCIDENT & EMERGENCY MEDICINE, EDUCATION & TRAINING (see Medical Education & Training), GENERAL MEDICINE (see Internal Medicine), MEDICAL EDUCATION & TRAINING |
| | |

SCHOLARONE™
Manuscripts

*I, the Submitting Author has the right to grant and does grant on behalf of all authors of the Work (as defined in the below author licence), an exclusive licence and/or a non-exclusive licence for contributions from authors who are: i) UK Crown employees; ii) where BMJ has agreed a CC-BY licence shall apply, and/or iii) in accordance with the terms applicable for US Federal Government officers or employees acting as part of their official duties; on a worldwide, perpetual, irrevocable, royalty-free basis to BMJ Publishing Group Ltd ("BMJ") its licensees and where the relevant Journal is co-owned by BMJ to the co-owners of the Journal, to publish the Work in this journal and any other BMJ products and to exploit all rights, as set out in our licence.*

*The Submitting Author accepts and understands that any supply made under these terms is made by BMJ to the Submitting Author unless you are acting as an employee on behalf of your employer or a postgraduate student of an affiliated institution which is paying any applicable article publishing charge ("APC") for Open Access articles. Where the Submitting Author wishes to make the Work available on an Open Access basis (and intends to pay the relevant APC), the terms of reuse of such Open Access shall be governed by a Creative Commons licence – details of these licences and which Creative Commons licence will apply to this Work are set out in our licence referred to above.*

*Other than as permitted in any relevant BMJ Author's Self Archiving Policies, I confirm this Work has not been accepted for publication elsewhere, is not being considered for publication elsewhere and does not duplicate material already published. I confirm all authors consent to publication of this Work and authorise the granting of this licence.*

For peer review only

# Use of simulation training to teach the ABCDE primary assessment: an observational study in a Dutch University Hospital with a 3-4 months follow up.

*Amanda M Drost-de Klerck, Tycho J Olgers, Evelien K van de Meeberg, Johanna Schönrock-Adema, Jan C ter Maaten.*

*Emergency Department, University Medical Center Groningen, Hanzeplein 1, 9713 GZ Groningen, The Netherlands, Amanda M Drost-de Klerck, Consultant Emergency Medicine. Emergency Department, University Medical Center Groningen, Tycho J Olgers, Consultant Acute Internal Medicine. Emergency Department, University Medical Center Groningen, Evelien K van de Meeberg, Consultant Emergency Medicine. University of Groningen and University Medical Center Groningen, Institute for Medical Education, Johanna Schönrock-Adema, senior researcher. Emergency Department, University Medical Center Groningen, Jan C ter Maaten, Full Professor Acute Internal Medicine.*

**Word count:** 3537

**Address for correspondence/request for reprints:**

A.M. Drost-de Klerck

Phone: +31503616161 / Fax: +31503611725

Email: a.drost@umcg.nl

For peer review only

## ABSTRACT

**Objectives** To investigate short- and long-term effectiveness of simulation training to acquire a structured Airway Breathing Circulation Disability Exposure (ABCDE) approach for medical emergencies; and to examine which skills were learned and maintained best.

**Design** an observational study with a 3-4 months follow-up.

**Setting** Skills centre of the University Medical Center Groningen.

**Participants** Thirty voluntary participants (21 females & 9 males; 27±2.77 years) of a simulation-based course.

**Intervention** A two-day ABCDE-teaching course for residents and non-residents. The course encompasses 24 simulations in which participants perform primary assessments of acute ill patients. Video recordings were taken of each participant performing a primary assessment, before (T1), directly after (T2) and three to four months after the intervention (T3).

**Main outcome measures** Physicians' performance in the ABCDE primary assessment at T1, T2 and T3. Two observers scored the primary assessments, blinded to measurement moment, using an assessment form to evaluate the performance with regard to skills essential for a structured ABCDE approach. The Friedman and Wilcoxon signed-rank test were used to compare physicians' performances on the subsequent measurement moments.

**Results** The mean ranks on the total primary assessment at T1, T2 and T3 were 1.14, 2.62 and 2.24, respectively, and were significantly different, ($p < 0.001$).

The mean ranks on the total primary assessment directly after the course (T2 vs. T1 $p<0.001$) and three to four months after the course (T3 vs. T1 $p<0.001$) were significantly better than before the course. Certain skills deteriorated during the follow-up. Strikingly, most skills that decrease over time are Crew Resources Management (CRM)-skills.


For peer review only

## Conclusion

A course using simulation training is an effective educational tool to teach physicians the ABCDE primary assessment. Certain CRM-skills decrease over time, so we recommend organizing refresher courses, simulation team training or another kind of simulation training with a focus on CRM-skills.

| **Strengths and limitations of this study** |
|---|
| **Strengths**<br>➢ This is an observational intervention study measuring the 3-months effect of a simulation course.<br>➢ This study used the same environment for intervention and study.<br>➢ The observers were blinded to measurement moment during this study on participants' performance on the primary assessment using the ABCDE approach.<br>**Limitations**<br>➢ The fact that the observers might have been also the instructor of some study participants, may have influenced their ratings for a few participants, but potential bias was minimized by offering the videos in random order and blinded to measurement moment, analysis showed a moderate to high inter-observer reliability and our study focused on the outcomes at group level and not individual outcomes.<br>➢ There might be bias in the fact that we do not know if the participants have prepared for the study scenario at T1 and T3 (the Hawthorne effect), whereas preparation for T3 might result in an overestimation of the actual course effectiveness at follow-up, preparation at T1 might result in an underestimation of the effectiveness of the course on both T2 and T3. |

For peer review only

## INTRODUCTION

### Background

In emergency medicine, assessing incoming patients in life-threatening conditions according to a structured approach is considered essential for successful resuscitation. The most widely used structured approach for early recognition and immediate treatment of life-threatening conditions is the "Airway-Breathing-Circulation-Disability-Exposure" (ABCDE) approach. The ABCDE approach is taught in the Advanced Trauma Life Support (ATLS) since 1978 and has been the standard approach in trauma since [1-3].

The use of the ABCDE primary assessment has also increased in other medical emergencies in recent years [4-7].

Using the ABCDE approach likely improves outcomes by helping health care professionals focus on the most life-threatening clinical problems and perform immediate resuscitation. Although solid empirical evidence for the usefulness of the ABCDE approach and its clinical benefits to patients is limited[1, 2], the importance of early treatment has been recognized in several emergencies such as trauma, stroke, sepsis and shock [1-5, 8-11].

### Importance

The Dutch inspection for healthcare requires that physicians treating non-trauma patients in the emergency department are ABCDE qualified [12]. Therefore, completing an ABCDE course is mandatory for physicians who work at the Emergency Department (ED). These courses usually contain lectures and simulation scenario training. Despite the wide use of simulation training for teaching the systematic ABCDE approach, little research has been done to analyse the effectiveness of simulation training in acquiring this structural approach. Simulation training has been proven to be effective for learning technical skills and

For peer review only



For peer review only - http://bmjopen.bmj.com/site/about/guidelines.xhtml

maintaining skills that are not frequently used in daily practice, like airway management and surgical skills [13-15]. Simulation training can also improve communication, efficiency and safety during teamwork [16-18]. A few studies based on self-perceptions showed that simulation training improved participants' confidence levels; they felt more competent in applying the ABCDE approach and several other skills [3, 19-21].

To our knowledge, it has not been investigated before whether simulation training actually improves physicians' skills in performing the structured ABCDE approach.

Our study focused on the effectiveness of simulation training to acquire a structured ABCDE approach. Our main goal was to analyse the short- and long-term effectiveness of simulation training to acquire a structured ABCDE approach. We analysed the improvement in physicians' primary assessment scores as a result of the ABCDE simulation training.

We also investigated whether the skills acquired were maintained over a period of three to four months and which skills and competences were learned and maintained best.

## METHODS

### Study design

We conducted an observational intervention study. The intervention consisted of a two-day simulation-based ABCDE teaching course. The measurements through video recordings were obtained before (T1), directly after (T2) and 3-4 months after the intervention (T3).

Three simulation scenarios (A, B and C) with different medical emergencies were specifically designed for this study. Scenario A was a case with pneumosepsis and hypoglycaemia, a partially obstructed airway due to low consciousness and shock. Scenario B concerned a case with obstructive shock caused by pulmonary embolism and an opioid overdose with altered

For peer review only

consciousness. Scenario C was a case with meningococcal sepsis with a partially obstructed airway due to low consciousness, bronchospasm and shock. We have designed three different and realistic scenarios with comparable difficulty by creating a life-threatening condition which needs resuscitation in three of the five main items from the ABCDE.

To prevent bias caused by the type or difficulty of the simulation, we varied the order in which participants had to complete the three simulation scenarios in such a way that the different scenario sequences were equally divided over T1, T2 and T3 (participant 1: T1 scenario A, T2 scenario B, T3 scenario C; participant 2: T1 scenario B, T2 scenario C, T3 scenario A; participant 3: T1 scenario C, T2 scenario A, T3 scenario B, etcetera). We made a schedule in which the order of the scenarios was prescribed for each participant and participants were divided over the schedule in order of inclusion.

We developed an assessment form (Figure 1) to evaluate the participants' performance regarding skills and competences essential to assess medical emergencies. The assessment form was divided in six categories; five concerned the ABCDE structure and the sixth contained remaining items. The remaining items focus on some Crew Resources Management (CRM) skills, like collaboration, communication, acknowledge own boundaries, and leadership. In each category, the skills or competences could be rated on a two- (agree, not agree, does not apply) or four-point scale (agree, partially agree, partially not agree, not agree or does not apply). We have added the option “does not apply”, because some skills were not required in some simulation scenarios. In the categories B, C, D and E also the number of examined items during the physical examination was scored. The following items could be scored; in the B: skin color, trachea position, respiratory rate, thorax excursions, breathing effort, lung percussion, lung auscultation and saturation; in the C: circulation of extremities, central pulse, heart rate, blood pressure, capillary refill, central venous

For peer review only

pressure, heart sounds; in the D: Glasgow Coma Scale (EMV), pupils, neck stiffness, glucose; in the E: temperature, head to toe examination (Figure 1).

**Intervention**

The ABCDE course is a two-day course for non-residents and first year residents which exists for ten years now. For most participants, it was a mandatory course that they need to pass before they were allowed to work in the ED. The course consisted mainly of simulation training and two theoretical lectures about airway management and Advanced Life Support (ALS). Previous to this course, the participants received a book with chapters describing the ABCDE approach and various acute medical emergencies.

The course focused on learning to recognize and treat life-threatening conditions, but also paid attention to some CRM-skills necessary for an efficient ABCDE approach.

This course was given in the skills centre in a room similar to a resuscitation room in the ED. The patient simulator used was a Laerdal Resusci Anne SkillTrainer with an upgrade Vitale Signs Sim Software Complete package. This simulator features heart and lung sounds, chest excursions, pulse and can show all vital signs on a separate monitor. With a separate computer, the sounds and vital signs can be changed during the scenario, to simulate several acute medical conditions.

Each course group consisted of six participants and two instructors. During the simulation rounds the group was split in half and two scenarios were run simultaneously in two separate rooms.

The course encompassed a total of 24 simulations with a patient simulator in which participants perform the primary assessment of acute ill patients. In each scenario, the role

For peer review only

of physician, “non-obstructive nurse” and observer were assigned to the three participants. One of the instructors operated the simulator and led the debriefing afterwards.

In eight scenarios, the participants fulfilled the role of physician; in the other scenarios, they carried out the role of “non-obstructing nurse” or observer.

The participants received a certificate if they passed the theoretical test and if they were, according to the instructors, capable of performing a structured primary assessment of an acute ill patient, with recognition and resuscitation of life-threatening conditions and adequate CRM-skills.

All course instructors have to follow a formalized educational program to become an instructor: First they have to pass the course as participant and have to work in the field of emergency medicine or acute care. Second they need to follow a two-day generic instructor course specifically developed for simulation training. Then they have to act as assistant-trainer for at least two courses and they need to write a report reflecting on their own role as instructor. Finally they are observed by an experienced instructor to become certified. As instructor, they have to teach the course least twice a year to stay competent and they need to follow the course-specific instructors day each year.

**Study setting and population**

This study was conducted in the same skills centre as were the course took place. During the video recordings, the simulator, materials and environment were also the same as during the course.

We approached all participants prior to this two-day course by e-mail and invited them to participate voluntarily in the study between August 2012 and December 2013. We

For peer review only

endeavoured to achieve a save response environment by a statement in the invitation e-mail that declining to participate in the study would not influence their course results.

The three measurement moments were scheduled in consultation with the participants, separate from the course. For each measurement moment, study participants were instructed to act in a simulation scenario as physician and to perform a primary assessment according to the ABCDE approach. One of the researchers participated as "non-obstructive" nurse and one researcher operated the simulator and computer.

**Patient and public involvement**

Participants of this study were not involved in the development of the research question, design or outcome measures. All participants of the study participated voluntarily, they knew all information about the investigation and they could withdraw from the study at any moment, all provided verbal consent.

**Study Protocol**

The first recording (T1) took place one to two weeks prior to the course. The second recording (T2) took place within one week after the course. The third recording (T3) took place between three to four months after the course.

The research team consisted of five physicians, who were also course instructors. They were all instructed in detail to only facilitate the simulation and not help the participant in any way.

The observers were two emergency physicians, who were also course instructors, but who were not part of the research team and therefore not involved in the recordings. The observers received specific instructions how to score each item on the assessment form.

For peer review only

They independently rated the recorded primary assessments in random order and were blinded to the measurement moment.

## Measurements

Each skill or competence on the assessment form had a lowest score of 0 and a highest score of 1, so the weight of each item was the same, independent of the two- (0 = not agree, 1 = agree, not applicable = missing value) or four-point scale (agree=1, partially agree=0.67, partially not agree=0.33, not agree=0, not applicable = missing value). This was the same for the number of examined items during the physical examination. For example in the B there was a maximum of 8 items to examine during physical examination. If one item was examined the score was 1/8 =0.125, if two items were examined the score was 2/8=0.25, if three items were examined, the score was 3/8=0.375, etc. So, the highest possible score on complete examination in the B was 8/8= 1.

Because some skills or competences were marked as not applicable, we calculated mean scores in each category (A, B, C, D, E and remaining items) based on the skills and competences which actually were applicable. In each category the maximal score to obtain was 1. Therefore, the maximal total score to obtain on the primary assessment for each scenario was 6 and the minimal score was 0.

## Data analysis

To perform the statistical analysis, IBM SPPSS version 23.0 was used. In all analyses, a p-value of < 0.05 was regarded as significant.

The inter-observer reliability between the scores given by the two observers for the three different time measurements was calculated using the Spearman's rank correlation test (for



For peer review only - http://bmjopen.bmj.com/site/about/guidelines.xhtml

T1, T2 and T3 (resp. R 0.81, 0.61, and 0.80). This inter-observer reliability was generally high enough to average the scores of the two observers for use in further analyses, as a correlation coefficient lower than 0.5 is considered as weak correlation, a correlation coefficient between 0.5 and 0.7 is considered as moderate correlation, a correlation coefficient between 0.7 and 0.9 is considered as high correlation and a correlation coefficient between 0.9 and 1 is considered as very high correlation.

We used the Friedman test for three related samples to analyse whether the total primary assessment scores of the entire group of participants differed between the three measurement moments. The Friedman test calculates and compares the mean ranks at T1, T2 and T3. The mean rank is calculated by ranking the score of each participant on T1, T2 and T3 and then calculating the mean rank of the entire group on T1, T2 and T3.

We used the Wilcoxon signed-rank test for two related samples to analyse whether the total primary assessment scores of the entire group of participants differed between two measurement moments and whether each skill or competence differed between two measurement moments. The Wilcoxon signed-rank test also uses the mean ranks.

Finally, we applied the Holm correction to reduce the possibility of getting a statistically significant result (Type I error) when performing multiple tests.

For peer review only

## RESULTS

### Characteristics of study subjects

Between August 2012 and December 2013, 27 courses were given to six participants each. From the total of 162 course participants 30 participants volunteered for this study. 21 were female, nine were male. Their mean age was 27 years (range 24-35, SD 2.77), their mean work experience was 11 months (range 0-48, SD14.4). Most participants did not have any



For peer review only - http://bmjopen.bmj.com/site/about/guidelines.xhtml

experience with simulation training at all (18 out of 30), some participants had done some training in their own department, like ALS or Basic Life Support (BLS) (7 out of 30), from five participants we do not know whether they had any experience with simulation training. The video recording of T3 of one participant was lost due to technical problems.

**Main results**

The mean ranks of the entire group on the total primary assessments at T1, T2 en T3 were 1.14, 2.62 and 2.24, respectively (Table 1), and they were significantly different, ($p < 0.001$). The mean rank on the total primary assessment at T2 (directly after the course) was significantly higher than the mean rank at T1 (before the course, table 1). The mean rank on the total primary assessment at T3 (3-4 months after the course) was significantly lower than the mean rank at T2, but remained significantly higher than the mean rank at T1 (Table 1 and Figure 2 ).

Table 1: Scores on the total primary assessment of the whole group at T1, T2 and T3

| Time | N | Median | Mean rank | Wilcoxon signed-rank test |
|---|---|---|---|---|
| 1 | 30 | 2.79 | 1.14 | T1 < T2, p<0.001 |
| 2 | 30 | 5.22 | 2.62 | T2 > T3, p<0.05 |
| 3 | 29 | 4.70 | 2.24 | T3 > T1, p<0.001 |

The mean ranks of the separate skills or competences were almost all significantly higher at T2 than at T1 (34 out of 40). With respect to the remaining skills, four could not be included in our analyses as they were scored too often as “does not apply”, which rendered the number of observations for those skills < N=10, which was too low to ascertain differences in a reliable way. For only three skills – “examines the airway”, “orders additional diagnostics in

For peer review only

the B" and "resuscitates adequately in the E"– we did not find a significant difference between T1 and T2.

Most of the separate skills did not show significant differences between mean rank at T2 and T3 (30 out of 40). Some skills (7 out of 40) had a significantly lower mean rank at T3 than at T2, but significantly higher than at T1 (Table 2).

Table 2: Outcomes for separate skills and competences that decreased between T2 and T3

| Skill/Competence | Wilcoxon signed-rank test | | |
|---|---|---|---|
| | N= | | |
| mentions abnormal findings in the C | 29 | T2 > T3<br>T1 < T3 | p<0.01<br>p<0.01 |
| recognizes live-threatening conditions in the C | 28 | T2 > T3<br>T1 < T3 | p<0.05<br>p<0.01 |
| mentions conclusions in the C | 28 | T2 > T3<br>T1 < T3 | p<0.05<br>p<0.01 |
| examines the D completely | 29 | T2 > T3<br>T1 < T3 | p<0.01<br>p<0.001 |
| communicates clearly | 29 | T2 > T3<br>T1 < T3 | p<0.05<br>p<0.001 |
| shows confidence | 29 | T2 > T3<br>T1 < T3 | p<0.05<br>p<0.001 |
| shows good leadership | 29 | T2 > T3<br>T1 < T3 | p<0.05<br>p<0.001 |

For peer review only

## DISCUSSION

Our study is the first to show the effectiveness of a course using simulation to teach physicians the ABCDE approach for the assessment of medically ill patients. We found that the positive effect on performing a primary assessment according the ABCDE approach persisted even 3-4 months after completing the course.

Our findings corroborate outcomes of other studies showing that simulation training in health professions education was consistently associated with large effects on knowledge, skills and behaviour [22, 23]. Our findings are also in line with previous research showing that simulation training establishes, corrects, and confirms knowledge and skills of the ABCDE approach and afterwards participants felt more competent in applying the ABCDE approach [3, 19-21].

In the follow-up we found decline in participant performance on some skills of the primary assessment. Strikingly, most skills that decrease over time are CRM-skills (Table 2). This is illustrated by a decrease in time of “recognition of life-threatening conditions in the C”, while the scores on the resuscitation skills did not decline. It is possible that this lower score reflects ‘not thinking out loud’ rather than failing to recognize a life-threatening condition. This decrease in CRM-skills suggests that focusing on CRM-skills by refresher courses or team training, after completing a simulation course, may be an important topic for physicians to maintain their skills. The positive effect of team training for these non-technical skills has already been shown [16-18].

Another skill that does not yield scores as high as most other skills after three to four months, is a complete examination of the Disability. A possible explanation for this finding is that the participants decide on the level of consciousness of the patient, determined by the EMV, whether it is necessary to examine certain components of the Disability. The

For peer review only

performance of the EMV does not decrease over time. This finding is in line with previous research from our group on primary assessment completeness showing that during the primary assessment in the emergency department, residents and experienced staff have equal, but not maximum ABCDE completeness scores (83 instead of 100) [7]. Fernandez et al. also showed that professional lifeguards failed to fully perform the ABCDE sequence and spend more time in the Circulation step, because they spent more time in steps considered most important [5].

These outcomes may reflect that a score of 100 on the ABCDE approach is not necessary to exclude potential life-threatening diseases or stabilize the patients.

**Limitations**

It is not possible to define the impact of the book and the lectures that are also part of the course, on the measured improvements in performance on the primary assessment. Outcomes from the regular course evaluation – not part of this study – indicated that the simulation training was the most powerful educational tool and accounted for most of the improvements. This feedback is in line with previous research showing that adding simulation training to a curriculum with lectures of medical students is associated with higher oral exam scores and higher overall course grades [22].

This study evaluated a course with instructors who are experts in the field of acute medicine, and experienced and certified course instructors. It is known that simulation-based education is most effective if guided by a safe and efficient debriefing and that debriefing can be challenging [23, 24]. We do not know if simulation training with debriefing by less experienced instructors may have less effect.

For peer review only

During the study we have deliberately chosen for a researcher participating as "non-obstructive nurse" in the measurement to minimize potential bias caused by help form the "non-obstructive nurse". The researcher knew the research questions and was instructed in detail to only follow instructions from the participant and not help in any way.

We did not schedule the researchers and operators with an equal distribution over the measurement moments, but all five researchers rotated between roles of the nurse and operator on own initiative. We therefor think that the bias of the non-obstructive nurse influencing the participant is negligible.

Another limitation of this study is that it was not possible to assess all specific skills in each simulation scenario, because they were often scored as "does not apply". The amount of not applicable rated items was between 0-3 in ten items, between 3-10 in five items, between 10-20 in two items and in four items the not applicable rated items was > 20. This limitation probably did not influence the results because items often scored as "does not apply" do not impact discriminating in quality of performance.

The sample size was chosen without power analysis, because we didn't know the expected effect. This relative small sample size of 30 participants already showed large significant differences. In our statistical analysis we accounted for a small sample size by using the Wilcoxon signed-rank test.

The observers may have been the instructor during the course of some study participants and we cannot exclude that this may have influenced their ratings for some of them. This potential bias was minimized by offering the videos in random order and blinding the observers to the measurement moment. Also, our study focused on the outcomes at group level and not individual outcomes and the inter-observer reliability was generally high.

For peer review only

The measurement moment of T3 varied between 3-4 months after T2. We do not know if this range of one month between T2 and T3 have caused a variation in performance at T3. Some participants had experience with simulation training. These participants might have had a higher score on T1 what might have caused an underestimation of the difference between T1 and T2.

Finally, the participants knew they were a study subject and when the recordings were scheduled. We do not know if they prepared for the study scenarios. Modifying behaviour in response to the awareness of being observed is known as the Hawthorne effect. This may have influenced the study scores at T1 and T3, whereas preparation for T3 might result in an overestimation of the actual course effectiveness at follow-up, preparation at T1 might result in an underestimation of the effectiveness of the course on both T2 and T3.

**Conclusion**

A course with simulation training is an effective educational tool to teach physicians to perform a structured primary assessment using the ABCDE. This competence is largely remained after three to four months. CRM-skills tend to decrease over time, so we recommend organizing refresher courses, simulation team training or another kind of simulation training with a focus on CRM-skills.

**Figure legend**

Figure 1  Assessment form used by the observers.

Figure 2 Line chart for total score on primary assessment for each participant on T1, T2 and T3.

For peer review only

## Contributors

AMDK and JCM have conceived the study and created the assessment form. AMDK, TJO, EKM and JCM collected the data. AMDK is guarantor and did the literature search, managed the data and performed data analysis and drafted the manuscript. JSA advised regarding to the data analysis. All authors contributed to the final manuscript, revision of the manuscript and final approval of the manuscript. The corresponding author attests that all listed authors meet authorship criteria and that no others meeting the criteria have been omitted.

## Competing interests

All authors have completed the ICMJE uniform disclosure form at www.icmje.org/coi_disclosure.pdf and declare: no support from any organisation for the submitted work; no financial relationships with any organisations that might have an interest in the submitted work in the previous three years; no other relationships or activities that could appear to have influenced the submitted work.

## Funding

This study was only funded by the Emergency Department of the University Medical Center Groningen.

## Data sharing statement

Unpublished statistical data will be available on request to the corresponding author.

## Ethics approval

Ethical approval was waived by our medical ethics committee (METc UMC Groningen) as this research is educational research. We have received an independent review board

For peer review only

declaration from our medical ethics committee, which declares that this study fulfills all the requirements for patient anonymity and is in agreement with regulations of our University Hospital.

**Transparency**

We declare that the manuscript is honest, accurate and transparent. No important aspects of the study have been omitted and discrepancies from the study as originally planned have been explained.

**Copyright/license for publication**

The corresponding author has the right to grant on behalf of all authors and does grant on behalf of all authors, a worldwide licence to the Publishers and its licensees in perpetuity, in all forms, formats and media (whether known now or created in the future), to i) publish, reproduce, distribute, display and store the Contribution, ii) translate the Contribution into other languages, create adaptations, reprints, include within collections and create summaries, extracts and/or, abstracts of the Contribution, iii) create any other derivative work(s) based on the Contribution, iv) to exploit all subsidiary rights in the Contribution, v) the inclusion of electronic links from the Contribution to third party material where-ever it may be located; and, vi) licence any third party to do any or all of the above."

**Acknowledgements**

We want to thank Kinge van der Heide for her help with the recordings. We want to thank Mirjam Doff-Holman and Martine Oosterloo for the assessment of all recordings.



For peer review only - http://bmjopen.bmj.com/site/about/guidelines.xhtml

**REFERENCES**

1 Soreide K. Three decades (1978-2008) of Advanced Trauma Life Support (ATLS) practice revised and evidence revisited. *Scand J Trauma Resusc Emerg Med* 2008;16:19,7241-16-19 doi:10.1186/1757-7241-16-19 [doi].

2 Jayaraman S, Sethi D, Chinnock P, et al. Advanced trauma life support training for hospital staff. *Cochrane Database Syst Rev* 2014;(8):CD004173. doi:CD004173 doi:10.1002/14651858.CD004173.pub4 [doi].

3 Abelsson A, Rystedt I, Suserud B, et al. Learning High-Energy Trauma Care Through Simulation. *CLIN SIMULATION NURS* 2018;17:1-6 doi:10.1016/j.ecns.2017.11.009.

4 Thim T, Krarup NHV, Grove EL, et al. Initial assessment and treatment with the Airway, Breathing, Circulation, Disability, Exposure (ABCDE) approach. *Int J Gen Med* 2012;5:117-21 doi:10.2147/IJGM.S28478.

5 Fernández-Méndez F, Otero-Agra M, Abelairas-Gómez C, et al. ABCDE approach to victims by lifeguards: How do they manage a critical patient? A cross sectional simulation study. *PLoS ONE* 2019;14 doi:10.1371/journal.pone.0212080.

6 Ilgen JS, Sherbino J, Cook DA. Technology-enhanced simulation in emergency medicine: a systematic review and meta-analysis. *Acad Emerg Med* 2013;20:117-27 doi:10.1111/acem.12076 [doi].

7 Olgers TJ, Dijkstra RS, Drost-de Klerck AM, et al. The ABCDE primary assessment in the emergency department in medically ill patients: an observational pilot study. *Neth J Med* 2017;75:106-11.

8 Franklin C, Mathew J. Developing strategies to prevent inhospital cardiac arrest: analyzing responses of physicians and nurses in the hours before the event. *Crit Care Med* 1994;22:244-7.

9 McQuillan P, Pilkington S, Allan A, et al. Confidential inquiry into quality of care before admission to intensive care. *BMJ* 1998;316:1853-8.

10 Kumar A, Roberts D, Wood KE, et al. Duration of hypotension before initiation of effective antimicrobial therapy is the critical determinant of survival in human septic shock. *Crit Care Med* 2006;34:1589-96 doi:10.1097/01.CCM.0000217961.75225.E9 [doi].

For peer review only

11 National Institute of Neurological Disorders and Stroke rt-PA Stroke Study Group. Tissue plasminogen activator for acute ischemic stroke. *N Engl J Med* 1995;333:1581-7 doi:10.1056/NEJM199512143332401 [doi].

12 Spoedeisende hulp: vanuit een stevige basis. Rapportage werkgroep levelindeling SEH. 2009 https://www.netwerkacutezorgnoordwest.nl/app/uploads/2017/01/200910-Rapport-SEH-Vanuit-een-stevig-basis.pdf.

13 Ryu WHA, Dharampal N, Mostafa AE, et al. Systematic Review of Patient-Specific Surgical Simulation: Toward Advancing Medical Education. *J Surg Educ* 2017 doi:S1931-7204(16)30269-0 [pii].

14 Price J, Naik V, Boodhwani M, et al. A randomized evaluation of simulation training on performance of vascular anastomosis on a high-fidelity in vivo model: the role of deliberate practice. *J Thorac Cardiovasc Surg* 2011;142:496-503 doi:10.1016/j.jtcvs.2011.05.015 [doi].

15 Kory PD, Eisen LA, Adachi M, et al. Initial airway management skills of senior residents: simulation training compared with traditional training. *Chest* 2007;132:1927-31 doi:S0012-3692(15)52468-9 [pii].

16 Buljac-Samardzic M, Dekker-van Doorn CM, van Wijngaarden JD, et al. Interventions to improve team effectiveness: a systematic review. *Health Policy* 2010;94:183-95 doi:10.1016/j.healthpol.2009.09.015 [doi].

17 Hesselink G, Berben S, Beune T, et al. Improving the governance of patient safety in emergency care: a systematic review of interventions. *BMJ Open* 2016;6:e009837,2015-009837 doi:10.1136/bmjopen-2015-009837 [doi].

18 Shapiro MJ, Morey JC, Small SD, et al. Simulation based teamwork training for emergency department staff: does it improve clinical team performance when added to an existing didactic teamwork curriculum?. *Qual Saf Health Care* 2004;13:417-21 doi:13/6/417 [pii].

19 Willett TG, Kirlew M, Cardinal P, et al. An evaluation of the Acute Critical Events Simulation (ACES) course for family medicine residents. *Can J Rural Med* 2011;16:89-95.

20 Carling J. Are graduate doctors adequately prepared to manage acutely unwell patients?. *Clin Teach* 2010;7:102-5 doi:10.1111/j.1743-498X.2010.00341.x [doi].

For peer review only

21 Pantelidis P, Staikoglou N, Paparoidamis G, et al. Medical students' satisfaction with the Applied Basic Clinical Seminar with Scenarios for Students, a novel simulation-based learning method in Greece. *J Educ Eval Health Prof* 2016;13:13 doi:10.3352/jeehp.2016.13.13 [doi].

22 Frallicciardi A, Fried J, Regan T, et al. A comparison of traditional lecture to simulation based cases for emergency medicine education among fourth year medical students. *Acad Emerg Med* 2012;19:S386 doi:10.1111/j.1553-2712.2012.01332.x.

23 Cook DA, Hatala R, Brydges R, et al. Technology-enhanced simulation for health professions education: a systematic review and meta-analysis. *JAMA* 2011;306:978-88 doi:10.1001/jama.2011.1234 [doi].

24 Grant VJ, Robinson T, Catena H, et al. Difficult debriefing situations: A toolbox for simulation educators. *Med Teach* 2018;40:703-12 doi:10.1080/0142159X.2018.1468558.

For peer review only

# Figure 1 Assessment form used by the observers.

| **Primary assessment** | | |
|---|---|---|
| A | - examines the airway<br>- mentions obstructed airway<br>- applies airway maneuvers<br>- applies oxygen | agree / disagree<br>agree / disagree/ d.n.a.<br>agree / partially agree / partially disagree / disagree / d.n.a.<br>agree / disagree/ d.n.a. |
| B | - examines B completely (color, trachea, resp. rate, excursions, breathing effort, percussion, auscultation, saturation)<br>- gives nurse the right orders<br>- mentions abnormal findings<br>- recognizes life-threatening conditions<br>- orders right additional diagnostic tests<br>- mentions conclusions/interpretation<br>- resuscitates adequately | 1 - 2 - 3 - 4 - 5 - 6 - 7 – 8<br><br>agree / partially agree / partially disagree / disagree / d.n.a.<br>agree / partially agree / partially disagree / disagree / d.n.a.<br>agree / disagree / d.n.a.<br>agree / disagree / d.n.a.<br>agree / partially agree / partially disagree / disagree<br>agree / partially agree / partially disagree / disagree / d.n.a. |
| C | - examines C completely (circulation of extremities, central pulse, heart rate, blood pressure, cap.refill, CVP, heart sounds)<br>- gives nurse the right orders<br>- mentions abnormal findings<br>- recognizes life-threatening conditions<br>- orders right additional diagnostic tests<br>- mentions conclusions/interpretation<br>- resuscitates adequately | 1 - 2 - 3 - 4 - 5 - 6 - 7<br><br>agree / partially agree / partially disagree / disagree / d.n.a.<br>agree / partially agree / partially disagree / disagree / d.n.a.<br>agree / disagree / d.n.a.<br>agree / disagree / d.n.a.<br>agree / partially agree / partially disagree / disagree<br>agree / partially agree / partially disagree / disagree / d.n.a. |
| D | - examines D completely (Glasgow Coma Scale, pupils, neck stiffness, glucose)<br>- applies EMC correctly<br>- mentions abnormal findings<br>- recognizes life-threatening conditions<br>- orders right additional diagnostic tests<br>- mentions conclusions/interpretation<br>- resuscitates adequately | 1 - 2 - 3 – 4<br><br>agree / partially agree / partially disagree / disagree<br>agree / partially agree / partially disagree / disagree/ d.n.a.<br>agree / disagree / d.n.a.<br>agree / disagree / d.n.a.<br>agree / partially agree / partially disagree / disagree<br>agree / partially agree / partially disagree / disagree / d.n.a. |
| E | - examines E completely (temperature, head to toe)<br>- gives nurse the right orders<br>- mentions abnormal findings<br>- orders right additional diagnostic tests<br>- mentions conclusions/interpretation<br>- resuscitates adequately | 1 – 2<br>agree / partially agree / partially disagree / disagree/ d.n.a.<br>agree / partially agree / partially disagree / disagree/ d.n.a.<br>agree / disagree / d.n.a.<br>agree / partially agree / partially disagree / disagree<br>agree / partially agree / partially disagree / disagree / d.n.a. |
| R<br>E<br>M<br>A<br>I<br>N<br>I<br>N<br>G | - asks for help adequately<br>- communicates clearly<br>- summarizes adequately<br>- draws the right conclusions<br>- clinical reasoning is adequate<br>- works structured<br>- stays calm<br>- shows confidence<br>- shows good leadership | agree / partially agree / partially disagree / disagree<br>agree / partially agree / partially disagree / disagree<br>agree / partially agree / partially disagree / disagree<br>agree / partially agree / partially disagree / disagree<br>agree / partially agree / partially disagree / disagree<br>agree / partially agree / partially disagree / disagree<br>agree / partially agree / partially disagree / disagree<br>agree / partially agree / partially disagree / disagree<br>agree / partially agree / partially disagree / disagree |

For peer review only

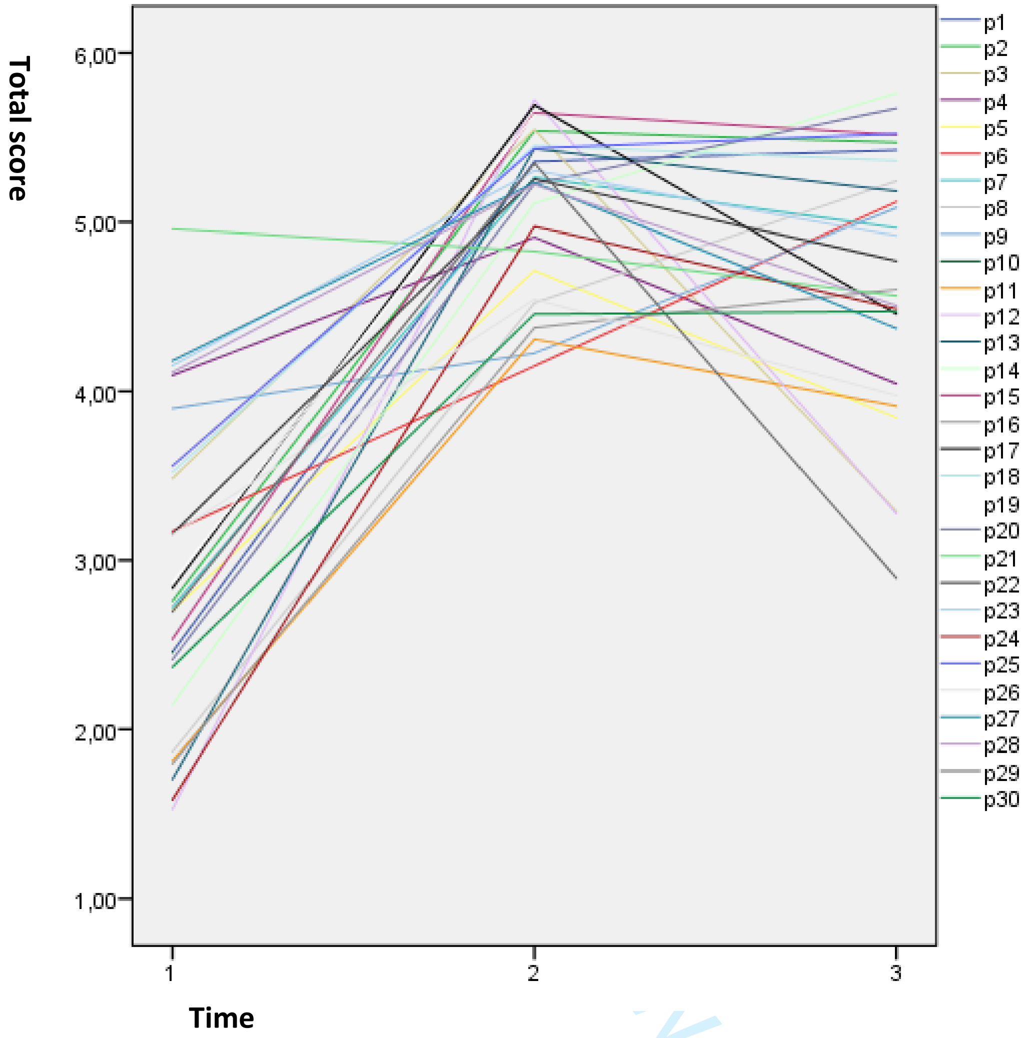


Figure 2: Line chart for total score on primary assessment for each participant on T1, T2 and T3. (p=participant)

STROBE Statement—checklist of items that should be included in reports of observational studies

| | Item No | Recommendation |
|---|---|---|
| **Title and abstract** | 1 | (*a*) Indicate the study's design with a commonly used term in the title or the abstract **Page 1** |
| | | (*b*) Provide in the abstract an informative and balanced summary of what was done and what was found **Page 4** |
| **Introduction** | | |
| Background/rationale | 2 | Explain the scientific background and rationale for the investigation being reported **Page 6 and 7** |
| Objectives | 3 | State specific objectives, including any prespecified hypotheses **Page 7** |
| **Methods** | | |
| Study design | 4 | Present key elements of study design early in the paper **Page 7-9** |
| Setting | 5 | Describe the setting, locations, and relevant dates, including periods of recruitment, exposure, follow-up, and data collection **Page 9-10** |
| Participants | 6 | (*a*) *Cohort study*—Give the eligibility criteria, and the sources and methods of selection of participants. Describe methods of follow-up **Page 7-11**<br>*Case-control study*—Give the eligibility criteria, and the sources and methods of case ascertainment and control selection. Give the rationale for the choice of cases and controls<br>*Cross-sectional study*—Give the eligibility criteria, and the sources and methods of selection of participants |
| | | (*b*) *Cohort study*—For matched studies, give matching criteria and number of exposed and unexposed<br>*Case-control study*—For matched studies, give matching criteria and the number of controls per case |
| Variables | 7 | Clearly define all outcomes, exposures, predictors, potential confounders, and effect modifiers. Give diagnostic criteria, if applicable **Page 7-12** |
| Data sources/ measurement | 8* | For each variable of interest, give sources of data and details of methods of assessment (measurement). Describe comparability of assessment methods if there is more than one group **Page 7-12** |
| Bias | 9 | Describe any efforts to address potential sources of bias **Page 7-12** |
| Study size | 10 | Explain how the study size was arrived at **(no effectsize known)** |
| Quantitative variables | 11 | Explain how quantitative variables were handled in the analyses. If applicable, describe which groupings were chosen and why |
| Statistical methods | 12 | (*a*) Describe all statistical methods, including those used to control for confounding **Page 12-13** |
| | | (*b*) Describe any methods used to examine subgroups and interactions |
| | | (*c*) Explain how missing data were addressed **Page 12** |
| | | (*d*) *Cohort study*—If applicable, explain how loss to follow-up was addressed<br>*Case-control study*—If applicable, explain how matching of cases and controls was addressed |


For peer review only - http://bmjopen.bmj.com/site/about/guidelines.xhtml

*Cross-sectional study*—If applicable, describe analytical methods taking account of sampling strategy

(*e*) Describe any sensitivity analyses **Page 12 (interobserver reliability)**

For peer review only



For peer review only - http://bmjopen.bmj.com/site/about/guidelines.xhtml

| | | |
|---|---|---|
| **Results** | | |
| Participants | 13* | (a) Report numbers of individuals at each stage of study—eg numbers potentially eligible, examined for eligibility, confirmed eligible, included in the study, completing follow-up, and analysed **Page 13** |
| | | (b) Give reasons for non-participation at each stage |
| | | (c) Consider use of a flow diagram |
| Descriptive data | 14* | (a) Give characteristics of study participants (eg demographic, clinical, social) and information on exposures and potential confounders **Page 13** |
| | | (b) Indicate number of participants with missing data for each variable of interest **Page 13 and supplemental file Outcome** |
| | | (c) *Cohort study*—Summarise follow-up time (eg, average and total amount) |
| Outcome data | 15* | *Cohort study*—Report numbers of outcome events or summary measures over time **Page 13-15** |
| | | *Case-control study*—Report numbers in each exposure category, or summary measures of exposure |
| | | *Cross-sectional study*—Report numbers of outcome events or summary measures |
| Main results | 16 | (*a*) Give unadjusted estimates and, if applicable, confounder-adjusted estimates and their precision (eg, 95% confidence interval). Make clear which confounders were adjusted for and why they were included **Page 13-15, Table 1 and 2** |
| | | (*b*) Report category boundaries when continuous variables were categorized |
| | | (*c*) If relevant, consider translating estimates of relative risk into absolute risk for a meaningful time period |
| Other analyses | 17 | Report other analyses done—eg analyses of subgroups and interactions, and sensitivity analyses **Page 12 (interobserver reliability)** |
| **Discussion** | | |
| Key results | 18 | Summarise key results with reference to study objectives **Page 15-16** |
| Limitations | 19 | Discuss limitations of the study, taking into account sources of potential bias or imprecision. Discuss both direction and magnitude of any potential bias **Page 17-18** |
| Interpretation | 20 | Give a cautious overall interpretation of results considering objectives, limitations, multiplicity of analyses, results from similar studies, and other relevant evidence **Page 15-18** |
| Generalisability | 21 | Discuss the generalisability (external validity) of the study results **Page 15-18** |
| **Other information** | | |
| Funding | 22 | Give the source of funding and the role of the funders for the present study and, if applicable, for the original study on which the present article is based **Page 3** |

*Give information separately for cases and controls in case-control studies and, if applicable, for exposed and unexposed groups in cohort and cross-sectional studies.



For peer review only - http://bmjopen.bmj.com/site/about/guidelines.xhtml

**Note:** An Explanation and Elaboration article discusses each checklist item and gives methodological background and published examples of transparent reporting. The STROBE checklist is best used in conjunction with this article (freely available on the Web sites of PLoS Medicine at http://www.plosmedicine.org/, Annals of Internal Medicine at http://www.annals.org/, and Epidemiology at http://www.epidem.com/). Information on the STROBE Initiative is available at www.strobe-statement.org.

For peer review only

BMJ Open

# Use of simulation training to teach the ABCDE primary assessment: an observational study in a Dutch University Hospital with a 3-4 months follow up.

| | |
|---|---|
| Journal: | *BMJ Open* |
| Manuscript ID | bmjopen-2019-032023.R4 |
| Article Type: | Original research |
| Date Submitted by the Author: | 10-May-2020 |
| Complete List of Authors: | Drost- de Klerck, Amanda; Universitair Medisch Centrum Groningen, emergency department<br>Olgers, Tycho; University Medical Center Groningen, Internal medicine, Emergency dept.<br>van de Meeberg, Evelien; Universitair Medisch Centrum Groningen, emergency department<br>Schonrock-Adema, Johanna; Universitair Medisch Centrum Groningen, emergency department<br>ter Maaten, Jan; University Medical Center Groningen, Emergency Department, Department of internal medicine |
| <b>Primary Subject Heading</b>: | Medical education and training |
| Secondary Subject Heading: | Emergency medicine |
| Keywords: | ACCIDENT & EMERGENCY MEDICINE, EDUCATION & TRAINING (see Medical Education & Training), GENERAL MEDICINE (see Internal Medicine), MEDICAL EDUCATION & TRAINING |
| | |

SCHOLARONE™
Manuscripts

*I, the Submitting Author has the right to grant and does grant on behalf of all authors of the Work (as defined in the below author licence), an exclusive licence and/or a non-exclusive licence for contributions from authors who are: i) UK Crown employees; ii) where BMJ has agreed a CC-BY licence shall apply, and/or iii) in accordance with the terms applicable for US Federal Government officers or employees acting as part of their official duties; on a worldwide, perpetual, irrevocable, royalty-free basis to BMJ Publishing Group Ltd ("BMJ") its licensees and where the relevant Journal is co-owned by BMJ to the co-owners of the Journal, to publish the Work in this journal and any other BMJ products and to exploit all rights, as set out in our licence.*

*The Submitting Author accepts and understands that any supply made under these terms is made by BMJ to the Submitting Author unless you are acting as an employee on behalf of your employer or a postgraduate student of an affiliated institution which is paying any applicable article publishing charge ("APC") for Open Access articles. Where the Submitting Author wishes to make the Work available on an Open Access basis (and intends to pay the relevant APC), the terms of reuse of such Open Access shall be governed by a Creative Commons licence – details of these licences and which Creative Commons licence will apply to this Work are set out in our licence referred to above.*

*Other than as permitted in any relevant BMJ Author's Self Archiving Policies, I confirm this Work has not been accepted for publication elsewhere, is not being considered for publication elsewhere and does not duplicate material already published. I confirm all authors consent to publication of this Work and authorise the granting of this licence.*

For peer review only

# Use of simulation training to teach the ABCDE primary assessment: an observational study in a Dutch University Hospital with a 3-4 months follow up.

*Amanda M Drost-de Klerck, Tycho J Olgers, Evelien K van de Meeberg, Johanna Schönrock-Adema, Jan C ter Maaten.*

*Emergency Department, University Medical Center Groningen, Hanzeplein 1, 9713 GZ Groningen, The Netherlands, Amanda M Drost-de Klerck, Consultant Emergency Medicine. Emergency Department, University Medical Center Groningen, Tycho J Olgers, Consultant Acute Internal Medicine. Emergency Department, University Medical Center Groningen, Evelien K van de Meeberg, Consultant Emergency Medicine. University of Groningen and University Medical Center Groningen, Institute for Medical Education, Johanna Schönrock-Adema, senior researcher. Emergency Department, University Medical Center Groningen, Jan C ter Maaten, Full Professor Acute Internal Medicine.*

For peer review only

**Word count:** 3674

**Address for correspondence/request for reprints:**

A.M. Drost-de Klerck

Phone: +31503616161 / Fax: +31503611725

Email: a.drost@umcg.nl

## ABSTRACT

**Objectives** To investigate short- and long-term effectiveness of simulation training to acquire a structured Airway Breathing Circulation Disability Exposure (ABCDE) approach for medical emergencies; and to examine which skills were learned and maintained best.

**Design** an observational study with a three to four months follow-up.

**Setting** Skills centre of the University Medical Center Groningen.

**Participants** Thirty voluntary participants (21 females & 9 males; 27±2.77 years) of a simulation-based course.

**Intervention** A two-day ABCDE-teaching course for residents and non-residents. The course encompasses 24 simulations in which participants perform primary assessments of acute ill patients. Video recordings were taken of each participant performing a primary assessment, before (T1), directly after (T2) and three to four months after the intervention (T3).

**Main outcome measures** Physicians' performance in the ABCDE primary assessment at T1, T2 and T3. Two observers scored the primary assessments, blinded to measurement moment, using an assessment form to evaluate the performance with regard to skills essential for a structured ABCDE approach. The Friedman and Wilcoxon signed-rank test were used to compare physicians' performances on the subsequent measurement moments.

**Results** The mean ranks on the total primary assessment at T1, T2 and T3 were 1.14, 2.62 and 2.24, respectively, and were significantly different, ($p < 0.001$).

The mean ranks on the total primary assessment directly after the course (T2 vs. T1 $p<0.001$) and three to four months after the course (T3 vs. T1 $p<0.001$) were significantly better than before the course. Certain skills deteriorated during the follow-up. Strikingly, most skills that decrease over time are Crew Resources Management (CRM)-skills.


For peer review only

**Conclusion**

A course using simulation training is an effective educational tool to teach physicians the ABCDE primary assessment. Certain CRM-skills decrease over time, so we recommend organizing refresher courses, simulation team training or another kind of simulation training with a focus on CRM-skills.

**Strengths and limitations of this study**

**Strengths**

- This is an observational study to investigate the short- and long-term effect of a simulation course.
- This study used the same environment for course and study.
- The observers were blinded to measurement moment during this study on participants' performance on the primary assessment using the ABCDE approach.

**Limitations**

- The fact that the observers might have been also the instructor of some study participants, may have influenced their ratings for a few participants, but potential bias was minimized by offering the videos in random order and blinded to measurement moment, analysis showed a moderate to high inter-observer reliability and our study focused on the outcomes at group level and not individual outcomes.
- There might be bias in the fact that we do not know if the participants have prepared for the study scenario at T1 and T3 (the Hawthorne effect), whereas preparation for T3 might result in an overestimation of the actual course effectiveness at follow-up, preparation at T1 might result in an underestimation of the effectiveness of the course on both T2 and T3.

For peer review only



For peer review only - http://bmjopen.bmj.com/site/about/guidelines.xhtml

## INTRODUCTION

### Background

In emergency medicine, assessing incoming patients in life-threatening conditions according to a structured approach is considered essential for successful resuscitation. The most widely used structured approach for early recognition and immediate treatment of life-threatening conditions is the “Airway-Breathing-Circulation-Disability-Exposure” (ABCDE) approach. The ABCDE approach is taught in the Advanced Trauma Life Support (ATLS) since 1978 and has been the standard approach in trauma since [1-3].

The use of the ABCDE primary assessment has also increased in other medical emergencies in recent years [4-7].

Using the ABCDE approach likely improves outcomes by helping health care professionals focus on the most life-threatening clinical problems and perform immediate resuscitation. Although solid empirical evidence for the usefulness of the ABCDE approach and its clinical benefits to patients is limited[1, 2], the importance of early treatment has been recognized in several emergencies such as trauma, stroke, sepsis and shock [1-5, 8-11].

### Importance

The Dutch inspection for healthcare requires that physicians treating non-trauma patients in the emergency department are ABCDE qualified [12]. Therefore, completing an ABCDE course is mandatory for physicians who work at the Emergency Department (ED). These courses usually contain lectures and simulation scenario training. Despite the wide use of simulation training for teaching the systematic ABCDE approach, little research has been done to analyse the effectiveness of simulation training in acquiring this structural approach. Simulation training has been proven to be effective for learning technical skills and

For peer review only

maintaining skills that are not frequently used in daily practice, like airway management and surgical skills [13-15]. Simulation training can also improve communication, efficiency and safety during teamwork [16-18]. A few studies based on self-perceptions showed that simulation training improved participants' confidence levels; they felt more competent in applying the ABCDE approach and several other skills [3, 19-21].

To our knowledge, it has not been investigated before whether simulation training actually improves physicians' skills in performing the structured ABCDE approach.

Our study focused on the effectiveness of simulation training to acquire a structured ABCDE approach. Our main goal was to analyse the short- and long-term effectiveness of simulation training to acquire a structured ABCDE approach. We analysed the improvement in physicians' primary assessment scores as a result of the ABCDE simulation training.

We also investigated whether the skills acquired were maintained over a period of three to four months and which skills and competences were learned and maintained best.

## METHODS

### Study design

We conducted an observational study to investigate short- and long-term effectiveness of a two-day simulation-based ABCDE teaching course. The measurements through video recordings were obtained before (T1), directly after (T2) and three to four months after the intervention (T3).

Three simulation scenarios (A, B and C) with different medical emergencies were specifically designed for this study. Scenario A was a case with pneumosepsis and hypoglycaemia, a partially obstructed airway due to low consciousness and shock. Scenario B concerned a case



For peer review only - http://bmjopen.bmj.com/site/about/guidelines.xhtml

with obstructive shock caused by pulmonary embolism and an opioid overdose with altered consciousness. Scenario C was a case with meningococcal sepsis with a partially obstructed airway due to low consciousness, bronchospasm and shock. We have designed three different and realistic scenarios with comparable difficulty by creating a life-threatening condition which needs resuscitation in three of the five main items from the ABCDE.

To prevent bias caused by the type or difficulty of the simulation, we varied the order in which participants had to complete the three simulation scenarios in such a way that the different scenario sequences were equally divided over T1, T2 and T3 (participant 1: T1 scenario A, T2 scenario B, T3 scenario C; participant 2: T1 scenario B, T2 scenario C, T3 scenario A; participant 3: T1 scenario C, T2 scenario A, T3 scenario B, etcetera). We made a schedule in which the order of the scenarios was prescribed for each participant and participants were divided over the schedule in order of inclusion.

We developed an assessment form (Figure 1) to evaluate the participants' performance regarding skills and competences essential to assess medical emergencies. The assessment form was divided in six categories; five concerned the ABCDE structure and the sixth contained remaining items. The remaining items focus on some Crew Resources Management (CRM) skills, like collaboration, communication, acknowledge own boundaries, and leadership. In each category, the skills or competences could be rated on a two- (agree, not agree, does not apply) or four-point scale (agree, partially agree, partially not agree, not agree or does not apply). We have added the option “does not apply”, because some skills were not required in some simulation scenarios. In the categories B, C, D and E also the number of examined items during the physical examination was scored. The following items could be scored; in the B: skin color, trachea position, respiratory rate, thorax excursions, breathing effort, lung percussion, lung auscultation and saturation; in the C: circulation of

For peer review only

extremities, central pulse, heart rate, blood pressure, capillary refill, central venous pressure, heart sounds; in the D: Glasgow Coma Scale (EMV), pupils, neck stiffness, glucose; in the E: temperature, head to toe examination (Figure 1).

**Intervention**

The ABCDE course is a two-day course for non-residents and first year residents which exists for ten years now. For most participants, it was a mandatory course that they need to pass before they were allowed to work in the ED. The course consisted mainly of simulation training and two theoretical lectures about airway management and Advanced Life Support (ALS). Previous to this course, the participants received a book with chapters describing the ABCDE approach and various acute medical emergencies.

The course focused on learning to recognize and treat life-threatening conditions, but also paid attention to some CRM-skills necessary for an efficient ABCDE approach.

This course was given in the skills centre in a room similar to a resuscitation room in the ED. The patient simulator used was a Laerdal Resusci Anne SkillTrainer with an upgrade Vitale Signs Sim Software Complete package. This simulator features heart and lung sounds, chest excursions, pulse and can show all vital signs on a separate monitor. With a separate computer, the sounds and vital signs can be changed during the scenario, to simulate several acute medical conditions.

Each course group consisted of six participants and two instructors. During the simulation rounds the group was split in half and two scenarios were run simultaneously in two separate rooms.

The course encompassed a total of 24 simulations with a patient simulator in which participants perform the primary assessment of acute ill patients. In each scenario, the role

For peer review only

of physician, “non-obstructive nurse” and observer were assigned to the three participants. One of the instructors operated the simulator and led the debriefing afterwards.

In eight scenarios, the participants fulfilled the role of physician; in the other scenarios, they carried out the role of “non-obstructing nurse” or observer.

The participants received a certificate if they passed the theoretical test and if they were, according to the instructors, capable of performing a structured primary assessment of an acute ill patient, with recognition and resuscitation of life-threatening conditions and adequate CRM-skills.

All course instructors have to follow a formalized educational program to become an instructor: First they have to pass the course as participant and have to work in the field of emergency medicine or acute care. Second they need to follow a two-day generic instructor course specifically developed for simulation training. Then they have to act as assistant-trainer for at least two courses and they need to write a report reflecting on their own role as instructor. Finally they are observed by an experienced instructor to become certified. As instructor, they have to teach the course least twice a year to stay competent and they need to follow the course-specific instructors day each year.

**Study setting and population**

This study was conducted in the same skills centre as were the course took place. During the video recordings, the simulator, materials and environment were also the same as during the course.

We approached all participants prior to this two-day course by e-mail and invited them to participate in the study between August 2012 and December 2013

For peer review only

We endeavoured to achieve a save response environment by a statement in the invitation e-mail that declining to participate in the study would not influence their course results. All participants participated voluntarily, they knew all information about the investigation and they could withdraw from the study at any moment, all provided verbal consent.

The three measurement moments were scheduled in consultation with the participants, separate from the course. For each measurement moment, study participants were instructed to act in a simulation scenario as physician and to perform a primary assessment according to the ABCDE approach. One of the researchers participated as “non-obstructive” nurse and one researcher operated the simulator and computer.

**Ethics approval**

Ethical approval was waived by our medical ethics committee (METc UMC Groningen) as this research is educational research. We have received an independent review board declaration from our medical ethics committee, which declares that this study fulfills all the requirements for patient anonymity and is in agreement with regulations of our University Hospital.

**Patient and public involvement**

Participants of this study were not involved in the development of the research question, design or outcome measures. Some participants of the study encouraged others to participate, but they were all voluntarily included. The results of the study will be available for the participants on request.

**Study Protocol**



For peer review only - http://bmjopen.bmj.com/site/about/guidelines.xhtml

The first recording (T1) took place one to two weeks prior to the course. The second recording (T2) took place within one week after the course. The third recording (T3) took place between three to four months after the course.

The research team consisted of five physicians, who were also course instructors. They were all instructed in detail to only facilitate the simulation and not help the participant in any way.

The observers were two emergency physicians, who were also course instructors, but who were not part of the research team and therefore not involved in the recordings. The observers received specific instructions how to score each item on the assessment form. They independently rated the recorded primary assessments in random order and were blinded to the measurement moment.

**Measurements**

Each skill or competence on the assessment form had a lowest score of 0 and a highest score of 1, so the weight of each item was the same, independent of the two- (0 = not agree, 1 = agree, not applicable = missing value) or four-point scale (agree=1, partially agree=0.67, partially not agree=0.33, not agree=0, not applicable = missing value). This was the same for the number of examined items during the physical examination. For example in the B there was a maximum of eight items to examine during physical examination. If one item was examined the score was 1/8 =0.125, if two items were examined the score was 2/8=0.25, if three items were examined, the score was 3/8=0.375, etc. So, the highest possible score on complete examination in the B was 8/8= 1.

Because some skills or competences were marked as not applicable, we calculated mean scores in each category (A, B, C, D, E and remaining items) based on the skills and


For peer review only - http://bmjopen.bmj.com/site/about/guidelines.xhtml

competences which actually were applicable. In each category the maximal score to obtain was 1. Therefore, the maximal total score to obtain on the primary assessment for each scenario was 6 and the minimal score was 0.

**Data analysis**

To perform the statistical analysis, IBM SPPSS version 23.0 was used. In all analyses, a p-value of < 0.05 was regarded as significant.

The inter-observer reliability between the scores given by the two observers for the three different time measurements was calculated using the Spearman's rank correlation test (for T1, T2 and T3 (resp. R 0.81, 0.61, and 0.80). This inter-observer reliability was generally high enough to average the scores of the two observers for use in further analyses, as a correlation coefficient lower than 0.5 is considered as weak correlation, a correlation coefficient between 0.5 and 0.7 is considered as moderate correlation, a correlation coefficient between 0.7 and 0.9 is considered as high correlation and a correlation coefficient between 0.9 and 1 is considered as very high correlation.

We used the Friedman test for three related samples to analyse whether the total primary assessment scores of the entire group of participants differed between the three measurement moments. The Friedman test calculates and compares the mean ranks at T1, T2 and T3. The mean rank is calculated on a scale from 1-3, because three measurements are ranked, 1 is the best rank and 3 the worst. The mean rank is calculated by ranking the score of each participant on T1, T2 and T3 and then calculating the mean rank of the entire group on T1, T2 and T3.

We used the Wilcoxon signed-rank test for two related samples to analyse whether the total primary assessment scores of the entire group of participants differed between two

For peer review only

measurement moments and whether each skill or competence differed between two measurement moments. The Wilcoxon signed-rank test also uses the mean ranks. Finally, we applied the Holm correction to reduce the possibility of getting a statistically significant result (Type I error) when performing multiple tests.

## RESULTS

### Characteristics of study subjects

Between August 2012 and December 2013, 27 courses were given to six participants each. From the total of 162 course participants 30 participants volunteered for this study. 21 were female, nine were male. Their mean age was 27 years (range 24-35, SD 2.77), their mean work experience was 11 months (range 0-48, SD14.4). Most participants did not have any experience with simulation training at all (18 out of 30), some participants had done some training in their own department, like ALS or Basic Life Support (BLS) (7 out of 30), from five participants we do not know whether they had any experience with simulation training. The video recording of T3 of one participant was lost due to technical problems.

### Main results

The median total score on the primary assessment was 2,79 at T1, 5.22 at T2 and 4.70 at T3 (Table 1 and Figure 2).

The mean ranks of the entire group on the total primary assessments at T1, T2 en T3 were 1.14, 2.62 and 2.24, respectively (Table 1), and they were significantly different, ($p < 0.001$). The mean rank on the total primary assessment at T2 (directly after the course) was significantly higher than the mean rank at T1 (before the course, table 1). The mean rank on the total primary assessment at T3 (three to four months after the course) was significantly



For peer review only - http://bmjopen.bmj.com/site/about/guidelines.xhtml

lower than the mean rank at T2, but remained significantly higher than the mean rank at T1 (Table 1).

Table 1: Scores on the total primary assessment of the whole group at T1, T2 and T3

| Time | N | Median | 25 percentile | 75 percentile | Mean rank | Wilcoxon signed-rank test |
|---|---|---|---|---|---|---|
| 1 | 30 | 2.79 | 2.31 | 3.53 | 1.14 | T1 < T2, $p<0.001$ |
| 2 | 30 | 5.22 | 4.57 | 5.43 | 2.62 | T2 > T3, $p<0.05$ |
| 3 | 29 | 4.70 | 4.20 | 5.30 | 2.24 | T3 > T1, $p<0.001$ |

The mean ranks of the separate skills or competences were almost all significantly higher at T2 than at T1 (34 out of 40). With respect to the remaining skills, four could not be included in our analyses as they were scored too often as “does not apply”, which rendered the number of observations for those skills < N=10, which was too low to ascertain differences in a reliable way. For only three skills – “examines the airway”, “orders additional diagnostics in the B” and “resuscitates adequately in the E”– we did not find a significant difference between T1 and T2.

Most of the separate skills did not show significant differences between mean rank at T2 and T3 (30 out of 40). Some skills (7 out of 40) had a significantly lower mean rank at T3 than at T2, but significantly higher than at T1 (Table 2).

Table 2: Outcomes for separate skills and competences that decreased between T2 and T3

| Skill/Competence | Wilcoxon signed-rank test | | |
|---|---|---|---|
| | N= | | |

For peer review only

| | | | |
|---|---|---|---|
| mentions abnormal findings in the C | 29 | T2 > T3<br>T1 < T3 | $p<0.01$<br>$p<0.01$ |
| recognizes live-threatening conditions in the C | 28 | T2 > T3<br>T1 < T3 | $p<0.05$<br>$p<0.01$ |
| mentions conclusions in the C | 28 | T2 > T3<br>T1 < T3 | $p<0.05$<br>$p<0.01$ |
| examines the D completely | 29 | T2 > T3<br>T1 < T3 | $p<0.01$<br>$p<0.001$ |
| communicates clearly | 29 | T2 > T3<br>T1 < T3 | $p<0.05$<br>$p<0.001$ |
| shows confidence | 29 | T2 > T3<br>T1 < T3 | $p<0.05$<br>$p<0.001$ |
| shows good leadership | 29 | T2 > T3<br>T1 < T3 | $p<0.05$<br>$p<0.001$ |

**DISCUSSION**

Our study is the first to show the effectiveness of a course using simulation to teach physicians the ABCDE approach for the assessment of medically ill patients. We found that the positive effect on performing a primary assessment according the ABCDE approach persisted even three to four months after completing the course.

Our findings corroborate outcomes of other studies showing that simulation training in health professions education was consistently associated with large effects on knowledge, skills and behaviour [22, 23]. Our findings are also in line with previous research showing that simulation training establishes, corrects, and confirms knowledge and skills of the ABCDE approach and afterwards participants felt more competent in applying the ABCDE approach [3, 19-21].

In the follow-up we found decline in participant performance on some skills of the primary assessment. Strikingly, most skills that decrease over time are CRM-skills (Table 2). This is illustrated by a decrease in time of “recognition of life-threatening conditions in the C”,

For peer review only

while the scores on the resuscitation skills did not decline. It is possible that this lower score reflects 'not thinking out loud' rather than failing to recognize a life-threatening condition. This decrease in CRM-skills suggests that focusing on CRM-skills by refresher courses or team training, after completing a simulation course, may be an important topic for physicians to maintain their skills. The positive effect of team training for these non-technical skills has already been shown [16-18].

Another skill that does not yield scores as high as most other skills after three to four months, is a complete examination of the Disability. A possible explanation for this finding is that the participants decide on the level of consciousness of the patient, determined by the EMV, whether it is necessary to examine certain components of the Disability. The performance of the EMV does not decrease over time. This finding is in line with previous research from our group on primary assessment completeness showing that during the primary assessment in the emergency department, residents and experienced staff have equal, but not maximum ABCDE completeness scores (83 instead of 100) [7]. Fernandez et al. also showed that professional lifeguards failed to fully perform the ABCDE sequence and spend more time in the Circulation step, because they spent more time in steps considered most important [5].

These outcomes may reflect that a score of 100 on the ABCDE approach is not necessary to exclude potential life-threatening diseases or stabilize the patients.

**Limitations**

It is not possible to define the impact of the book and the lectures that are also part of the course, on the measured improvements in performance on the primary assessment. Outcomes from the regular course evaluation – not part of this study – indicated that the



For peer review only - http://bmjopen.bmj.com/site/about/guidelines.xhtml

simulation training was the most powerful educational tool and accounted for most of the improvements. This feedback is in line with previous research showing that adding simulation training to a curriculum with lectures of medical students is associated with higher oral exam scores and higher overall course grades [22].

This study evaluated a course with instructors who are experts in the field of acute medicine, and experienced and certified course instructors. It is known that simulation-based education is most effective if guided by a safe and efficient debriefing and that debriefing can be challenging [23, 24]. We do not know if simulation training with debriefing by less experienced instructors may have less effect.

During the study we have deliberately chosen for a researcher participating as "non-obstructive nurse" in the measurement to minimize potential bias caused by help form the "non-obstructive nurse". The researcher knew the research questions and was instructed in detail to only follow instructions from the participant and not help in any way.

We did not schedule the researchers and operators with an equal distribution over the measurement moments, but all five researchers rotated between roles of the nurse and operator on own initiative. We therefor think that the bias of the non-obstructive nurse influencing the participant is negligible.

Another limitation of this study is that it was not possible to assess all specific skills in each simulation scenario, because they were often scored as "does not apply". The amount of not applicable rated items was between 0-3 in ten items, between 3-10 in five items, between 10-20 in two items and in four items the not applicable rated items was > 20. This limitation probably did not influence the results because items often scored as "does not apply" do not impact discriminating in quality of performance.

For peer review only

The sample size was chosen without power analysis, because we didn't know the expected effect. This relative small sample size of 30 participants already showed large significant differences. In our statistical analysis we accounted for a small sample size by using the Wilcoxon signed-rank test.

The observers may have been the instructor during the course of some study participants and we cannot exclude that this may have influenced their ratings for some of them. This potential bias was minimized by offering the videos in random order and blinding the observers to the measurement moment. Also, our study focused on the outcomes at group level and not individual outcomes and the inter-observer analysis showed a moderate to high inter-observer reliability.

The measurement moment of T3 varied between three to four months after T2. We do not know if this range of one month between T2 and T3 have caused a variation in performance at T3.

Some participants had experience with simulation training. These participants might have had a higher score on T1 what might have caused an underestimation of the difference between T1 and T2.

Finally, the participants knew they were a study subject and when the recordings were scheduled. We do not know if they prepared for the study scenarios. Modifying behaviour in response to the awareness of being observed is known as the Hawthorne effect. This may have influenced the study scores at T1 and T3, whereas preparation for T3 might result in an overestimation of the actual course effectiveness at follow-up, preparation at T1 might result in an underestimation of the effectiveness of the course on both T2 and T3.

**Conclusion**

For peer review only



For peer review only - http://bmjopen.bmj.com/site/about/guidelines.xhtml

A course with simulation training is an effective educational tool to teach physicians to perform a structured primary assessment using the ABCDE. This competence is largely remained after three to four months. CRM-skills tend to decrease over time, so we recommend organizing refresher courses, simulation team training or another kind of simulation training with a focus on CRM-skills.

**Figure legend**

Figure 1 Assessment form used by the observers.

Figure 2 Boxplot showing median and interquartile range of total score on primary assessment at T1, T2 and T3.

**Contributors**

AMDK and JCM have conceived the study and created the assessment form. AMDK, TJO, EKM and JCM collected the data. AMDK is guarantor and did the literature search, managed the data and performed data analysis and drafted the manuscript. JSA advised regarding to the data analysis. All authors contributed to the final manuscript, revision of the manuscript and final approval of the manuscript. The corresponding author attests that all listed authors meet authorship criteria and that no others meeting the criteria have been omitted.

**Competing interests**

All authors have completed the ICMJE uniform disclosure form at www.icmje.org/coi_disclosure.pdf and declare: no support from any organisation for the

For peer review only

submitted work; no financial relationships with any organisations that might have an interest in the submitted work in the previous three years; no other relationships or activities that could appear to have influenced the submitted work.

**Funding**

This study was only funded by the Emergency Department of the University Medical Center Groningen.

**Data sharing statement**

Unpublished statistical data will be available on request to the corresponding author.

**Transparency**

We declare that the manuscript is honest, accurate and transparent. No important aspects of the study have been omitted and discrepancies from the study as originally planned have been explained.

**Copyright/license for publication**

The corresponding author has the right to grant on behalf of all authors and does grant on behalf of all authors, a worldwide licence to the Publishers and its licensees in perpetuity, in all forms, formats and media (whether known now or created in the future), to i) publish, reproduce, distribute, display and store the Contribution, ii) translate the Contribution into other languages, create adaptations, reprints, include within collections and create summaries, extracts and/or, abstracts of the Contribution, iii) create any other derivative work(s) based on the Contribution, iv) to exploit all subsidiary rights in the Contribution, v)

For peer review only

the inclusion of electronic links from the Contribution to third party material where-ever it may be located; and, vi) licence any third party to do any or all of the above."

**Acknowledgements**

We want to thank Kinge van der Heide for her help with the recordings. We want to thank Mirjam Doff-Holman and Martine Oosterloo for the assessment of all recordings.

**REFERENCES**

1 Soreide K. Three decades (1978-2008) of Advanced Trauma Life Support (ATLS) practice revised and evidence revisited. *Scand J Trauma Resusc Emerg Med* 2008;16:19,7241-16-19 doi:10.1186/1757-7241-16-19 [doi].

2 Jayaraman S, Sethi D, Chinnock P, et al. Advanced trauma life support training for hospital staff. *Cochrane Database Syst Rev* 2014;(8):CD004173. doi:CD004173 doi:10.1002/14651858.CD004173.pub4 [doi].

3 Abelsson A, Rystedt I, Suserud B, et al. Learning High-Energy Trauma Care Through Simulation. *CLIN SIMULATION NURS* 2018;17:1-6 doi:10.1016/j.ecns.2017.11.009.

4 Thim T, Krarup NHV, Grove EL, et al. Initial assessment and treatment with the Airway, Breathing, Circulation, Disability, Exposure (ABCDE) approach. *Int J Gen Med* 2012;5:117-21 doi:10.2147/IJGM.S28478.

5 Fernández-Méndez F, Otero-Agra M, Abelairas-Gómez C, et al. ABCDE approach to victims by lifeguards: How do they manage a critical patient? A cross sectional simulation study. *PLoS ONE* 2019;14 doi:10.1371/journal.pone.0212080.

6 Ilgen JS, Sherbino J, Cook DA. Technology-enhanced simulation in emergency medicine: a systematic review and meta-analysis. *Acad Emerg Med* 2013;20:117-27 doi:10.1111/acem.12076 [doi].

7 Olgers TJ, Dijkstra RS, Drost-de Klerck AM, et al. The ABCDE primary assessment in the emergency department in medically ill patients: an observational pilot study. *Neth J Med* 2017;75:106-11.

For peer review only

8 Franklin C, Mathew J. Developing strategies to prevent inhospital cardiac arrest: analyzing responses of physicians and nurses in the hours before the event. *Crit Care Med* 1994;22:244-7.

9 McQuillan P, Pilkington S, Allan A, et al. Confidential inquiry into quality of care before admission to intensive care. *BMJ* 1998;316:1853-8.

10 Kumar A, Roberts D, Wood KE, et al. Duration of hypotension before initiation of effective antimicrobial therapy is the critical determinant of survival in human septic shock. *Crit Care Med* 2006;34:1589-96 doi:10.1097/01.CCM.0000217961.75225.E9 [doi].

11 National Institute of Neurological Disorders and Stroke rt-PA Stroke Study Group. Tissue plasminogen activator for acute ischemic stroke. *N Engl J Med* 1995;333:1581-7 doi:10.1056/NEJM199512143332401 [doi].

12 Spoedeisende hulp: vanuit een stevige basis. Rapportage werkgroep levelindeling SEH. 2009 https://www.netwerkacutezorgnoordwest.nl/app/uploads/2017/01/200910-Rapport-SEH-Vanuit-een-stevig-basis.pdf.

13 Ryu WHA, Dharampal N, Mostafa AE, et al. Systematic Review of Patient-Specific Surgical Simulation: Toward Advancing Medical Education. *J Surg Educ* 2017 doi:S1931-7204(16)30269-0 [pii].

14 Price J, Naik V, Boodhwani M, et al. A randomized evaluation of simulation training on performance of vascular anastomosis on a high-fidelity in vivo model: the role of deliberate practice. *J Thorac Cardiovasc Surg* 2011;142:496-503 doi:10.1016/j.jtcvs.2011.05.015 [doi].

15 Kory PD, Eisen LA, Adachi M, et al. Initial airway management skills of senior residents: simulation training compared with traditional training. *Chest* 2007;132:1927-31 doi:S0012-3692(15)52468-9 [pii].

16 Buljac-Samardzic M, Dekker-van Doorn CM, van Wijngaarden JD, et al. Interventions to improve team effectiveness: a systematic review. *Health Policy* 2010;94:183-95 doi:10.1016/j.healthpol.2009.09.015 [doi].

17 Hesselink G, Berben S, Beune T, et al. Improving the governance of patient safety in emergency care: a systematic review of interventions. *BMJ Open* 2016;6:e009837,2015-009837 doi:10.1136/bmjopen-2015-009837 [doi].



For peer review only - http://bmjopen.bmj.com/site/about/guidelines.xhtml

18 Shapiro MJ, Morey JC, Small SD, et al. Simulation based teamwork training for emergency department staff: does it improve clinical team performance when added to an existing didactic teamwork curriculum?. *Qual Saf Health Care* 2004;13:417-21 doi:13/6/417 [pii].

19 Willett TG, Kirlew M, Cardinal P, et al. An evaluation of the Acute Critical Events Simulation (ACES) course for family medicine residents. *Can J Rural Med* 2011;16:89-95.

20 Carling J. Are graduate doctors adequately prepared to manage acutely unwell patients?. *Clin Teach* 2010;7:102-5 doi:10.1111/j.1743-498X.2010.00341.x [doi].

21 Pantelidis P, Staikoglou N, Paparoidamis G, et al. Medical students' satisfaction with the Applied Basic Clinical Seminar with Scenarios for Students, a novel simulation-based learning method in Greece. *J Educ Eval Health Prof* 2016;13:13 doi:10.3352/jeehp.2016.13.13 [doi].

22 Fralicciardi A, Fried J, Regan T, et al. A comparison of traditional lecture to simulation based cases for emergency medicine education among fourth year medical students. *Acad Emerg Med* 2012;19:S386 doi:10.1111/j.1553-2712.2012.01332.x.

23 Cook DA, Hatala R, Brydges R, et al. Technology-enhanced simulation for health professions education: a systematic review and meta-analysis. *JAMA* 2011;306:978-88 doi:10.1001/jama.2011.1234 [doi].

24 Grant VJ, Robinson T, Catena H, et al. Difficult debriefing situations: A toolbox for simulation educators. *Med Teach* 2018;40:703-12 doi:10.1080/0142159X.2018.1468558.

For peer review only


For peer review only - http://bmjopen.bmj.com/site/about/guidelines.xhtml

# Figure 1 Assessment form used by the observers.

| Primary assessment | | |
|---|---|---|
| A | - examines the airway | agree / disagree |
| | - mentions obstructed airway | agree / disagree/ d.n.a. |
| | - applies airway maneuvers | agree / partially agree / partially disagree / disagree / d.n.a. |
| | - applies oxygen | agree / disagree/ d.n.a. |
| B | - examines B completely (color, trachea, resp. rate, excursions, breathing effort, percussion, auscultation, saturation) | 1 - 2 - 3 - 4 - 5 - 6 - 7 – 8 |
| | - gives nurse the right orders | agree / partially agree / partially disagree / disagree / d.n.a. |
| | - mentions abnormal findings | agree / partially agree / partially disagree / disagree / d.n.a. |
| | - recognizes life-threatening conditions | agree / disagree / d.n.a. |
| | - orders right additional diagnostic tests | agree / disagree / d.n.a. |
| | - mentions conclusions/interpretation | agree / partially agree / partially disagree / disagree |
| | - resuscitates adequately | agree / partially agree / partially disagree / disagree / d.n.a. |
| C | - examines C completely (circulation of extremities, central pulse, heart rate, blood pressure, cap.refill, CVP, heart sounds) | 1 - 2 - 3 - 4 - 5 - 6 - 7 |
| | - gives nurse the right orders | agree / partially agree / partially disagree / disagree / d.n.a. |
| | - mentions abnormal findings | agree / partially agree / partially disagree / disagree / d.n.a. |
| | - recognizes life-threatening conditions | agree / disagree / d.n.a. |
| | - orders right additional diagnostic tests | agree / disagree / d.n.a. |
| | - mentions conclusions/interpretation | agree / partially agree / partially disagree / disagree |
| | - resuscitates adequately | agree / partially agree / partially disagree / disagree / d.n.a. |
| D | - examines D completely (Glasgow Coma Scale, pupils, neck stiffness, glucose) | 1 - 2 - 3 – 4 |
| | - applies EMC correctly | agree / partially agree / partially disagree / disagree |
| | - mentions abnormal findings | agree / partially agree / partially disagree / disagree/ d.n.a. |
| | - recognizes life-threatening conditions | agree / disagree / d.n.a. |
| | - orders right additional diagnostic tests | agree / disagree / d.n.a. |
| | - mentions conclusions/interpretation | agree / partially agree / partially disagree / disagree |
| | - resuscitates adequately | agree / partially agree / partially disagree / disagree / d.n.a. |
| E | - examines E completely (temperature, head to toe) | 1 – 2 |
| | - gives nurse the right orders | agree / partially agree / partially disagree / disagree/ d.n.a. |
| | - mentions abnormal findings | agree / partially agree / partially disagree / disagree/ d.n.a. |
| | - orders right additional diagnostic tests | agree / disagree / d.n.a. |
| | - mentions conclusions/interpretation | agree / partially agree / partially disagree / disagree |
| | - resuscitates adequately | agree / partially agree / partially disagree / disagree / d.n.a. |
| R | - asks for help adequately | agree / partially agree / partially disagree / disagree |
| E | - communicates clearly | agree / partially agree / partially disagree / disagree |
| M | - summarizes adequately | agree / partially agree / partially disagree / disagree |
| A | - draws the right conclusions | agree / partially agree / partially disagree / disagree |
| I | - clinical reasoning is adequate | agree / partially agree / partially disagree / disagree |
| N | - works structured | agree / partially agree / partially disagree / disagree |
| I | - stays calm | agree / partially agree / partially disagree / disagree |
| N | - shows confidence | agree / partially agree / partially disagree / disagree |
| G | - shows good leadership | agree / partially agree / partially disagree / disagree |

For peer review only - http://bmjopen.bmj.com/site/about/guidelines.xhtml

Figure 2: Boxplot showing median and interquartile range of total score on primary assessment at T1, T2 and T3.

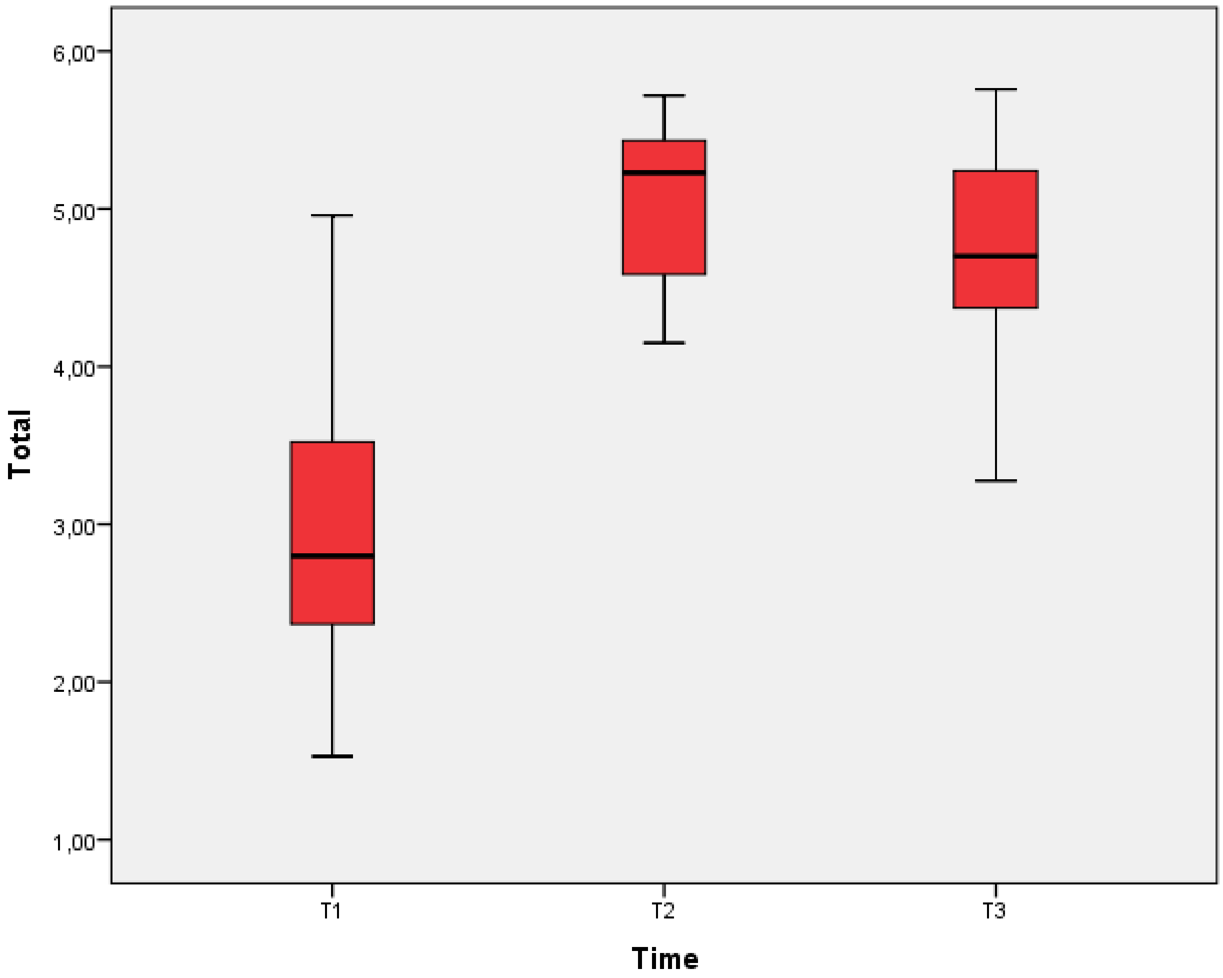

STROBE Statement—checklist of items that should be included in reports of observational studies

| | Item No | Recommendation |
|---|---|---|
| **Title and abstract** | 1 | (*a*) Indicate the study's design with a commonly used term in the title or the abstract **Page 1** |
| | | (*b*) Provide in the abstract an informative and balanced summary of what was done and what was found **Page 4** |
| **Introduction** | | |
| Background/rationale | 2 | Explain the scientific background and rationale for the investigation being reported **Page 6 and 7** |
| Objectives | 3 | State specific objectives, including any prespecified hypotheses **Page 7** |
| **Methods** | | |
| Study design | 4 | Present key elements of study design early in the paper **Page 7-9** |
| Setting | 5 | Describe the setting, locations, and relevant dates, including periods of recruitment, exposure, follow-up, and data collection **Page 9-10** |
| Participants | 6 | (*a*) *Cohort study*—Give the eligibility criteria, and the sources and methods of selection of participants. Describe methods of follow-up **Page 7-11**<br>*Case-control study*—Give the eligibility criteria, and the sources and methods of case ascertainment and control selection. Give the rationale for the choice of cases and controls<br>*Cross-sectional study*—Give the eligibility criteria, and the sources and methods of selection of participants |
| | | (*b*) *Cohort study*—For matched studies, give matching criteria and number of exposed and unexposed<br>*Case-control study*—For matched studies, give matching criteria and the number of controls per case |
| Variables | 7 | Clearly define all outcomes, exposures, predictors, potential confounders, and effect modifiers. Give diagnostic criteria, if applicable **Page 7-12** |
| Data sources/ measurement | 8* | For each variable of interest, give sources of data and details of methods of assessment (measurement). Describe comparability of assessment methods if there is more than one group **Page 7-12** |
| Bias | 9 | Describe any efforts to address potential sources of bias **Page 7-12** |
| Study size | 10 | Explain how the study size was arrived at **(no effectsize known)** |
| Quantitative variables | 11 | Explain how quantitative variables were handled in the analyses. If applicable, describe which groupings were chosen and why |
| Statistical methods | 12 | (*a*) Describe all statistical methods, including those used to control for confounding **Page 12-13** |
| | | (*b*) Describe any methods used to examine subgroups and interactions |
| | | (*c*) Explain how missing data were addressed **Page 12** |
| | | (*d*) *Cohort study*—If applicable, explain how loss to follow-up was addressed<br>*Case-control study*—If applicable, explain how matching of cases and controls was addressed |


For peer review only - http://bmjopen.bmj.com/site/about/guidelines.xhtml

*Cross-sectional study*—If applicable, describe analytical methods taking account of sampling strategy

(*e*) Describe any sensitivity analyses **Page 12 (interobserver reliability)**

For peer review only



For peer review only - http://bmjopen.bmj.com/site/about/guidelines.xhtml

| | | |
|---|---|---|
| **Results** | | |
| Participants | 13* | (a) Report numbers of individuals at each stage of study—eg numbers potentially eligible, examined for eligibility, confirmed eligible, included in the study, completing follow-up, and analysed **Page 13** |
| | | (b) Give reasons for non-participation at each stage |
| | | (c) Consider use of a flow diagram |
| Descriptive data | 14* | (a) Give characteristics of study participants (eg demographic, clinical, social) and information on exposures and potential confounders **Page 13** |
| | | (b) Indicate number of participants with missing data for each variable of interest **Page 13 and supplemental file Outcome** |
| | | (c) *Cohort study*—Summarise follow-up time (eg, average and total amount) |
| Outcome data | 15* | *Cohort study*—Report numbers of outcome events or summary measures over time **Page 13-15** |
| | | *Case-control study*—Report numbers in each exposure category, or summary measures of exposure |
| | | *Cross-sectional study*—Report numbers of outcome events or summary measures |
| Main results | 16 | (*a*) Give unadjusted estimates and, if applicable, confounder-adjusted estimates and their precision (eg, 95% confidence interval). Make clear which confounders were adjusted for and why they were included **Page 13-15, Table 1 and 2** |
| | | (*b*) Report category boundaries when continuous variables were categorized |
| | | (*c*) If relevant, consider translating estimates of relative risk into absolute risk for a meaningful time period |
| Other analyses | 17 | Report other analyses done—eg analyses of subgroups and interactions, and sensitivity analyses **Page 12 (interobserver reliability)** |
| **Discussion** | | |
| Key results | 18 | Summarise key results with reference to study objectives **Page 15-16** |
| Limitations | 19 | Discuss limitations of the study, taking into account sources of potential bias or imprecision. Discuss both direction and magnitude of any potential bias **Page 17-18** |
| Interpretation | 20 | Give a cautious overall interpretation of results considering objectives, limitations, multiplicity of analyses, results from similar studies, and other relevant evidence **Page 15-18** |
| Generalisability | 21 | Discuss the generalisability (external validity) of the study results **Page 15-18** |
| **Other information** | | |
| Funding | 22 | Give the source of funding and the role of the funders for the present study and, if applicable, for the original study on which the present article is based **Page 3** |

*Give information separately for cases and controls in case-control studies and, if applicable, for exposed and unexposed groups in cohort and cross-sectional studies.



For peer review only - http://bmjopen.bmj.com/site/about/guidelines.xhtml

**Note:** An Explanation and Elaboration article discusses each checklist item and gives methodological background and published examples of transparent reporting. The STROBE checklist is best used in conjunction with this article (freely available on the Web sites of PLoS Medicine at http://www.plosmedicine.org/, Annals of Internal Medicine at http://www.annals.org/, and Epidemiology at http://www.epidem.com/). Information on the STROBE Initiative is available at www.strobe-statement.org.

For peer review only


For peer review only - http://bmjopen.bmj.com/site/about/guidelines.xhtml
